# Kineochelins—A New Group of Siderophores From an Antarctic Bacterium

**DOI:** 10.1111/1751-7915.70386

**Published:** 2026-05-28

**Authors:** Stanislava Kralova, Peter Spacek, Johannes Gafriller, Matej Bezdicek, Viktoria Medvedcova, Joana Séneca, Jay Osvatic, Ulrike Grienke, Thomas Rattei, Olga N. Sekurova, Sergey B. Zotchev, Martin Zehl, Alexander Loy

**Affiliations:** ^1^ Division of Microbial Ecology, Centre for Microbiology and Environmental Systems Science University of Vienna Vienna Austria; ^2^ Department of Molecular Pharmacy, Faculty of Pharmacy Masaryk University Brno Czech Republic; ^3^ Department of Pharmaceutical Sciences University of Vienna Vienna Austria; ^4^ Vienna Doctoral School of Pharmaceutical, Nutritional and Sport Sciences University of Vienna Vienna Austria; ^5^ Division of Clinical Microbiology and Immunology, Department of Laboratory Medicine University Hospital Brno Brno Czech Republic; ^6^ Division of Clinical Microbiology and Immunology, Department of Laboratory Medicine, Faculty of Medicine Masaryk University Brno Czech Republic; ^7^ Department of Pharmacology, Faculty of Medicine Pavol Jozef Šafárik University Košice Slovakia; ^8^ Joint Microbiome Facility of the Medical University of Vienna and the University of Vienna, Medical University of Vienna, University of Vienna Vienna Austria; ^9^ Department of Laboratory Medicine Medical University of Vienna Vienna Austria; ^10^ Division of Computational Systems Biology, Centre for Microbiology and Environmental Systems Science University of Vienna Vienna Austria; ^11^ Department of Analytical Chemistry, Faculty of Chemistry University of Vienna Vienna Austria; ^12^ Institute of Science and Technology Austria (ISTA) Klosterneuburg Austria; ^13^ Austrian Polar Research Institute Vienna Austria

**Keywords:** *Actinokineospora*, Antarctica, antimicrobial discovery, biosynthetic gene cluster, genome mining, microbial competition, nonribosomal peptide synthetase, siderophores

## Abstract

The global rise of antimicrobial resistance has intensified the search for new microbial metabolites from underexplored environments and taxonomic groups. Extreme and geographically isolated habitats such as Antarctic terrestrial ecosystems represent promising reservoirs of biosynthetic diversity, particularly among rare and difficult‐to‐cultivate actinomycetes that may produce chemically diverse metabolites with potential biotechnological applications. Here, we report the characterization of kineochelins, a previously undescribed group of siderophores produced by the Antarctic isolate *Actinokineospora* sp. UV203, representing a difficult‐to‐cultivate actinomycete lineage. Structural elucidation revealed a set of closely related congeners with a mixed‐ligand architecture consistent with metal‐chelating activity. Genome mining combined with transcriptomic analysis identified a dedicated nonribosomal peptide synthetase‐encoding biosynthetic gene cluster responsible for kineochelin production. Comparative genomic analyses indicated that, although kineochelin biosynthetic genes share limited similarity with known mixed‐ligand siderophores, their gene content and organization differ substantially, suggesting a distinct biosynthetic lineage. Functional characterization of the culture supernatant and an enriched pre‐purified kineochelin fraction demonstrated strong and selective iron chelation, with high affinity for ferric and ferrous iron. Crude culture extracts inhibited the growth of bacterial strains isolated from the same Antarctic environment, indicating that kineochelins may contribute to iron‐mediated microbial competition. In addition, kineochelin‐enriched pre‐purified fractions showed moderate selective inhibitory activity against the opportunistic yeast pathogen *Nakaseomyces glabratus* and a clinical isolate of *Saccharomyces cerevisiae* associated with invasive infection. These findings expand the chemical and biosynthetic diversity known within the genus *Actinokineospora* and demonstrate that Antarctic rare actinomycetes represent valuable sources of previously unexplored natural products. The discovery of kineochelins highlights the potential of genome‐guided exploration of polar microorganisms for identifying bioactive metabolites with relevance for antimicrobial discovery and biotechnology.

## Graphical abstract

Genome‐guided analysis of an Antarctic *Actinokineospora* strain led to the discovery of kineochelins, a new group of siderophores involved in iron sequestration and microbial competition. Kineochelin production inhibits co‐occurring bacteria and clinically relevant yeasts, highlighting Antarctic microorganisms as promising sources of bioactive natural products.

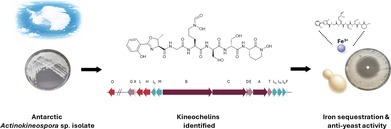

## 1 Introduction

Antimicrobial resistance is an escalating global crisis that threatens to erode decades of medical progress, already causing millions of deaths annually (Murray et al. 2022). The alarming spread of multidrug‐resistant microorganisms, ‘superbugs’, has created an urgent need for new classes of antimicrobials with novel modes of action (Cardona et al. 2025; World Health Organization 2023). Yet, the antibiotic discovery pipeline has been in steady decline, with most approved drugs representing derivatives of existing scaffolds rather than structurally innovative compounds (Butler et al. 2023). Strikingly, of the 13 new antimicrobial drugs approved between 2017 and 2023, only two met the World Health Organization's innovation criteria (Cardona et al. 2025; World Health Organization 2023). To overcome this stagnation, efforts have also explored non–natural product strategies such as bacteriophages, nanoparticles and monoclonal antibodies (Kirienko et al. 2019; El‐Sayed et al. 2026). Nevertheless, the search for structurally novel natural products (NPs) remains central, with increasing focus on underexplored habitats that may harbour organisms with distinctive biosynthetic repertoires and provide access to untapped chemical space (Masschelein et al. 2017; Hegemann et al. 2022; Barry 2025).

Extreme and geographically isolated environments have emerged as important reservoirs of microbial diversity with a high potential for the discovery of novel NPs (Quinn and Dyson 2024). In such habitats, strong selective pressures, including temperature extremes, nutrient limitation, desiccation and radiation, favour microorganisms with specialized metabolic and biosynthetic capabilities, often resulting in chemically distinctive specialized (secondary) metabolites (SMs), commonly referred to as NPs (Bej et al. 2010). Antarctic terrestrial ecosystems represent one of the most extreme examples of these conditions, combining chronic low temperatures with recurrent freeze–thaw events, seasonal fluctuations in light and nutrients and long‐term ecological isolation (Medeiros et al. 2025; Ramasamy et al. 2023; Pearce 2012). These constraints have shaped highly adapted microbial communities in which SMs play central roles in resource acquisition, stress tolerance and interspecies interactions (Medeiros et al. 2025; Sayed et al. 2020; Tytgat et al. 2023; Vyverman et al. 2010). Antarctic microbiota have attracted increasing attention as a source of structurally and functionally diverse NPs with biomedical and biotechnological relevance (Waschulin et al. 2022; Medeiros et al. 2024; Benaud et al. 2020; Silva et al. 2020). However, despite extensive cultivation‐independent surveys revealing a rich diversity of biosynthetic gene clusters (BGCs) in Antarctic microbes, relatively few of these predicted pathways have been linked to purified metabolites or experimentally validated biosynthetic systems (Medeiros et al. 2025). This disconnection between genomic potential and chemical characterization highlights the need for integrated approaches that couple cultivation, metabolite isolation and genomic analysis to link biosynthetic capacity with expressed chemistry and microbial interactions in Antarctic soil communities.

Among the metabolite classes likely to play key ecological roles in such nutrient‐limited environments, siderophores are of particular interest (Kramer et al. 2020; Li et al. 2025; Boiteau et al. 2016). Siderophores are specialized low‐molecular‐weight metabolites within the broader family of metallophores that enable microorganisms to acquire iron under conditions where ferric iron is poorly soluble and therefore scarcely bioavailable (Hider and Kong 2010). Siderophore production represents one of the most widespread microbial strategies for overcoming iron limitation in diverse environments, including host‐associated niches (Kramer et al. 2020; Guerinot 1994; Miethke and Marahiel 2007). Bacterial siderophores display remarkable structural diversity and are commonly classified into four major groups according to the functional groups primarily involved in ferric iron coordination: catecholate, hydroxamate, carboxylate and mixed‐ligand families (Hider and Kong 2010; LeBlanc and Wuest 2024; Gomes et al. 2024; Ahmed and Holmström 2014). Additional subclasses include phenolate/salicylate and heterocyclic oxazoline‐ or thiazoline‐containing siderophores (LeBlanc and Wuest 2024). These metabolites adopt diverse linear, cyclic and mixed architectures, and the majority of known examples are biosynthesized through nonribosomal peptide synthetase (NRPS)‐dependent or NRPS‐independent pathways (Reitz and Medema 2022). Siderophore production can strongly influence fitness, persistence and colonization success (Figueiredo et al. 2022; Chandrangsu et al. 2017). In addition to simply facilitating iron acquisition for the producing organism, siderophores serve as key ecological mediators. They influence microbial competition, cooperation, host interactions and community assembly through mechanisms such as differential access to iron and siderophore piracy (Kramer et al. 2020; Ahmed and Holmström 2014; Schalk 2025; Gu et al. 2025).

Siderophores have also gained increasing attention in the context of antimicrobial resistance (AMR) (Rayner et al. 2023). Owing to their specialized biosynthetic pathways and highly selective uptake systems, they have emerged as attractive targets for antimicrobial intervention and molecular delivery strategies (Reitz and Medema 2022; Ribeiro and Simões 2019; Frei et al. 2020; Wei et al. 2025). In particular, siderophore‐mediated transport can be exploited in a ‘Trojan Horse’ approach, in which antibiotics are covalently linked to siderophore‐like moieties to form the so‐called sideromycins. The siderophore‐like moiety facilitates the active uptake of the antibiotic moiety even into resistant bacterial cells resistant to such antibiotic due to membrane impermeability (Gräff and Barry 2024). This strategy is exemplified by the naturally occurring sideromycins Albomycin and Salmycin B (Rayner et al. 2023), and has been successfully translated into the clinic with the siderophore cephalosporin conjugate Cefiderocol (Parsels et al. 2021). In parallel, siderophores and related metallophores may be used to restrict pathogen growth by sequestering essential metals or disrupting metal homeostasis (Golden et al. 2024; Mayegowda and Gadilingappa 2025). Beyond clinical applications, microbial metallophores are increasingly recognized as versatile tools in biotechnology, with emerging roles in bioremediation, metal recovery, biosensing and the modulation of microbial communities (Kramer et al. 2020; Saha et al. 2016; Gräff and Barry 2024).

Against this background, Antarctic microorganisms remain an underexplored source of structurally distinctive siderophores and other specialized metabolites. In this study, we established a bacterial culture collection from Antarctic soils sampled at the James Ross Island (2007–2023) that consists of strains of diverse phylogenetic origin and with broad genomic potential for SM production. Using a selected strain that represents a potentially novel *Actinokineospora* species, we show how this resource can be exploited for the discovery of previously unknown secondary metabolites. The BGC repertoire and liquid chromatography–mass spectrometry (LC–MS)‐based SM analysis of *Actinokineospora* sp. UV203 guided the isolation and structure elucidation of a new group of mixed‐ligand siderophores with antimicrobial properties, which we named kineochelins.

## 2 Results

### 2.1 Isolation, Taxonomic Placement and Biosynthetic Gene Clusters of *Actinokineospora* sp. UV203

**FIGURE 1:**
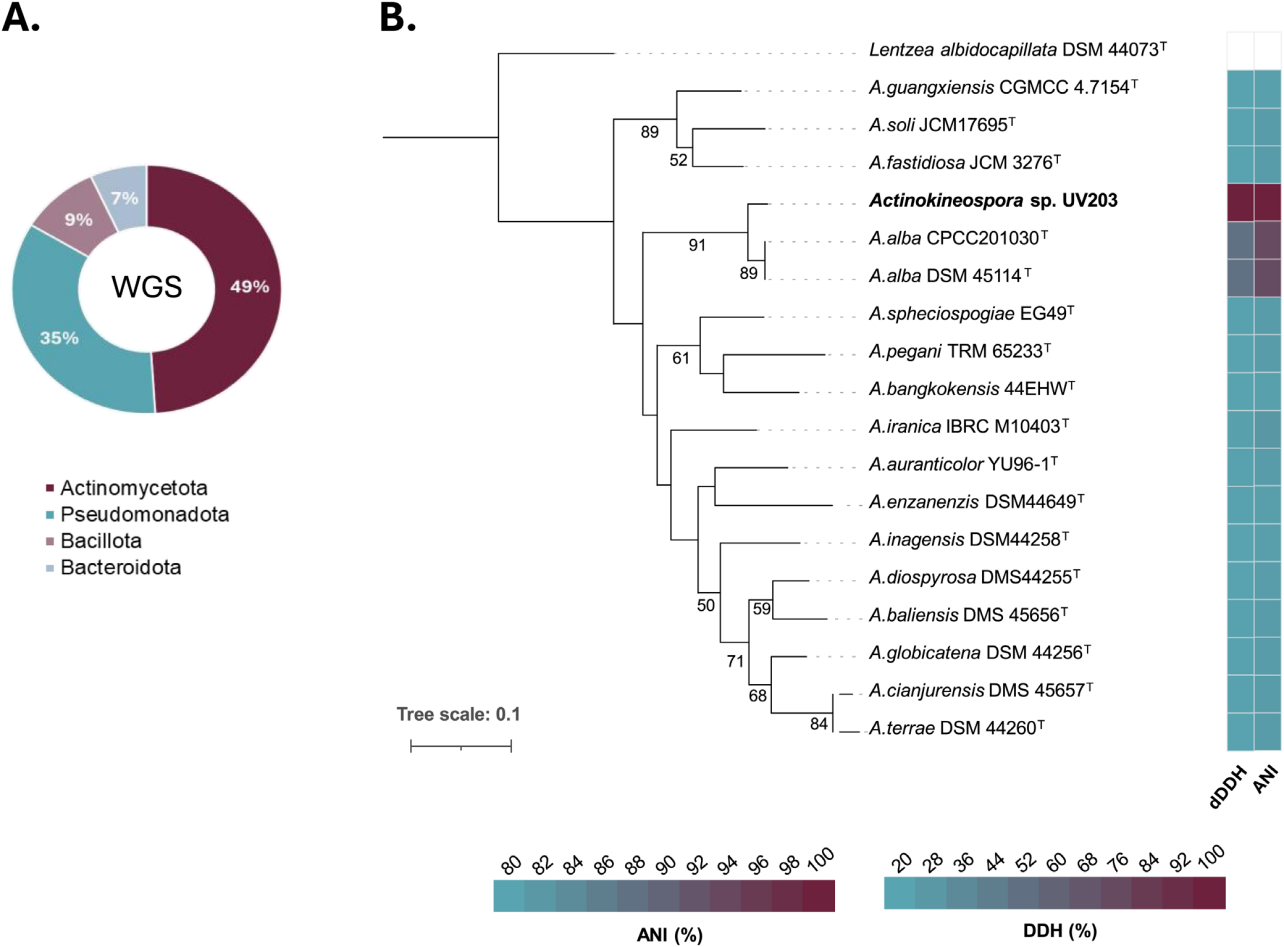
Phylogeny of strain UV203 suggests it represents a yet undescribed *Actinokineospora* species in the Actinomycetota. (A) Phylum‐level composition of the isolates selected for whole‐genome sequencing (*n* = 145), based on genome‐derived taxonomic assignments. (B) Genome‐scale up‐to‐date bacterial core gene (UBCG) phylogeny placed strain UV203 as a distinct lineage within *Actinokineospora*; scale bar indicates 0.1 substitutions per site. The numbers at the nodes indicate the gene support index (maximum value, 91) (Kim et al. 2021). Pairwise genomic relatedness between UV203 and type strains of validly published *Actinokineospora* species with available genomes is shown as heatmaps of average nucleotide identity (ANI, %; FastANI) and digital DNA–DNA hybridization (dDDH, %; GGDC) (Meier‐Kolthoff et al. 2021; Jain et al. 2018). Colour scales shown below.

As part of a long‐term biodiversity campaign on James Ross Island, Antarctica (since 2007), we established a collection of 972 morphologically and phylogenetically diverse bacterial strains that were isolated from the active layer of soils above permafrost. Preliminary classification based on 16S rRNA gene sequencing and phylogenetic analysis assigned most of these isolates to four phyla: Actinomycetota, Bacillota, Bacteroidota and Pseudomonadota (Figure 1A). A subset of phylogenetically divergent strains (*n* = 145), prioritized based on 16S rRNA gene sequence dissimilarity and unique phylogenetic branching patterns, was selected for whole‐genome sequencing. Most genome‐sequenced strains, including strain UV203, represent previously undescribed species or higher taxa. Strain UV203 was isolated as a colony on starch‐casein agar from a pretreated active layer soil sample collected during the austral summer of 2022 at Abernethy Flats. Phylogenomic analysis suggested that strain UV203 may represent a new species within the *Actinokineospora* genus. This classification was supported by genome distance metrics, including average nucleotide identity (ANI: 93.6%) and digital DNA–DNA hybridization (dDDH: 51.8%) values to the closest related species *Actinokineospora alba* DSM 45114^T^, both falling below the accepted thresholds for species delineation (Konstantinidis 2023). Core genes within the up‐to‐date bacterial core gene (UBCG) pipeline (Kim et al. 2021) further supported the placement of UV203 as a distinct species‐level lineage (Figure 1B). Although genome sequences are not yet available for three validly described *Actinokineospora* species ( *Actinokineospora riparia* , *Actinokineospora cibodasensis* and *Actinokineospora acnipugnans*), 16S rRNA gene phylogeny indicated that strain UV203 does not cluster with these taxa either (Figure S1).

To further explore the biosynthetic potential of *Actinokineospora* sp. UV203, we combined genome‐based prediction of BGCs and metabolomic analysis using an untargeted LC–MS workflow. antiSMASH predicted 21 BGCs, many without close homologues in public databases (Table 1). Manual inspection of the antiSMASH output indicated that this number is likely an underestimate, as closely spaced or partially overlapping BGCs are not always fully resolved by automated prediction algorithms, a known limitation of automated BGC boundary prediction (Blin et al. 2021). The production of > 28 SMs was detected under various tested culturing conditions, whereby many of them could be grouped together as congeners either by tentative identification or because the MS/MS spectra strongly indicated shared substructures (Table S1). In NP research, the term congeners usually refers to a group of compounds produced by the same BGC, thus sharing the same core scaffold but differing in size, specific substituents or functional groups (IUPAC 1997). Next to several SMs that were tentatively identified, such as gaburedins A–D and F (Sidda et al. 2013), a large number of them yielded MS and MS/MS spectra that could not be matched to any known NP present in GNPS (Wang et al. 2016), NPAtlas (Poynton et al. 2025) or the CAS database. We thus attempted the isolation and characterization of selected metabolites that could not be matched to known NPs in the databases.

**TABLE 1:** Tabulated view of all detected BGCs in the UV203 genome with antiSMASH v7.1.

Region^a^	BGC type	Position on contig	Closest match in MIBiG database^b^	Similarity (%)^c^
2.1	NRPS, lanthipeptide‐class‐I and class‐II	258,185–326,154	Microtermolide A	13
2.2	Terpene, azole‐containing‐RiPP, T1PKS	467,192–566,193	Kedarcidin	6
2.3	NRPS	957,733–1,015,948	Friulimicins	12
2.4	NRPS, T1PKS, NRPS‐like, arylpolyene	1,105,806–1,213,951	Cinnapeptin	75
2.5	NRPS	1,217,308–1,284,184	Madurastatins	4
2.6	Hydrogen‐cyanide	1,365,883–1,378,661	Aborycin	14
2.7	hglE‐KS	1,455,103–1,502,323	Hexacosalactone A	11
2.8	Terpene, RiPP‐like	1,615,723–1,646,526	Pyrroloformamides	12
2.9	Oligosaccharide	1,839,250–1,863,060	Kinamycin	8
2.10	Terpene	2,253,547–2,278,659	Isorenieratene	37
2.11	Lanthipeptide‐class‐III, NRP‐metallophore, NRPS	2,666,494–2,744,358	Gobichelin A/B	38
2.12	NRPS‐like	2,756,202–2,797,764	Mannopeptimycin	7
2.13	NRPS‐like, betalactone	2,914,781–2,956,598	Lasalocid	9
2.14	Ectoine	3,685,227–3,695,628	Ectoine	100
2.15	NAPAA	4,591,137–4,625,024	Desertomycins	5
2.16	NRPS‐like, ectoine, T1PKS	5,370,866–5,435,668	Sporolide A/B	44
2.17	NI‐siderophore	5,515,984–5,547,414	Peucechelin	15
2.18	Betalactone, NRPS‐like, NRPS	5,606,360–5,676,892	Coelibactin	45
2.19	Azole‐containing‐RiPP	5,967,859–5,996,139		
2.20	RiPP‐like	6,036,301–6,047,107		

Abbreviations: hglE‐KS, heterocyst glycolipid synthase‐like PKS; NAPAA, non‐alpha poly‐amino acids like ε‐polylysin; NI‐siderophore, NRPS‐independent IucA/IucC‐like siderophore; NRP‐metallophore, non‐ribosomal peptide metallophore; NRPS, non‐ribosomal peptide synthetase; RiPP, ribosomally synthesized and post‐translationally modified peptide; T1PKS, type I polyketide synthase.

^a^ All BGCs were detected on contig 2 (6,462,755 bp).

^b^ Minimum information about a biosynthetic gene cluster.

^c^ As determined by antiSMASH v7.1.

**FIGURE 2:**
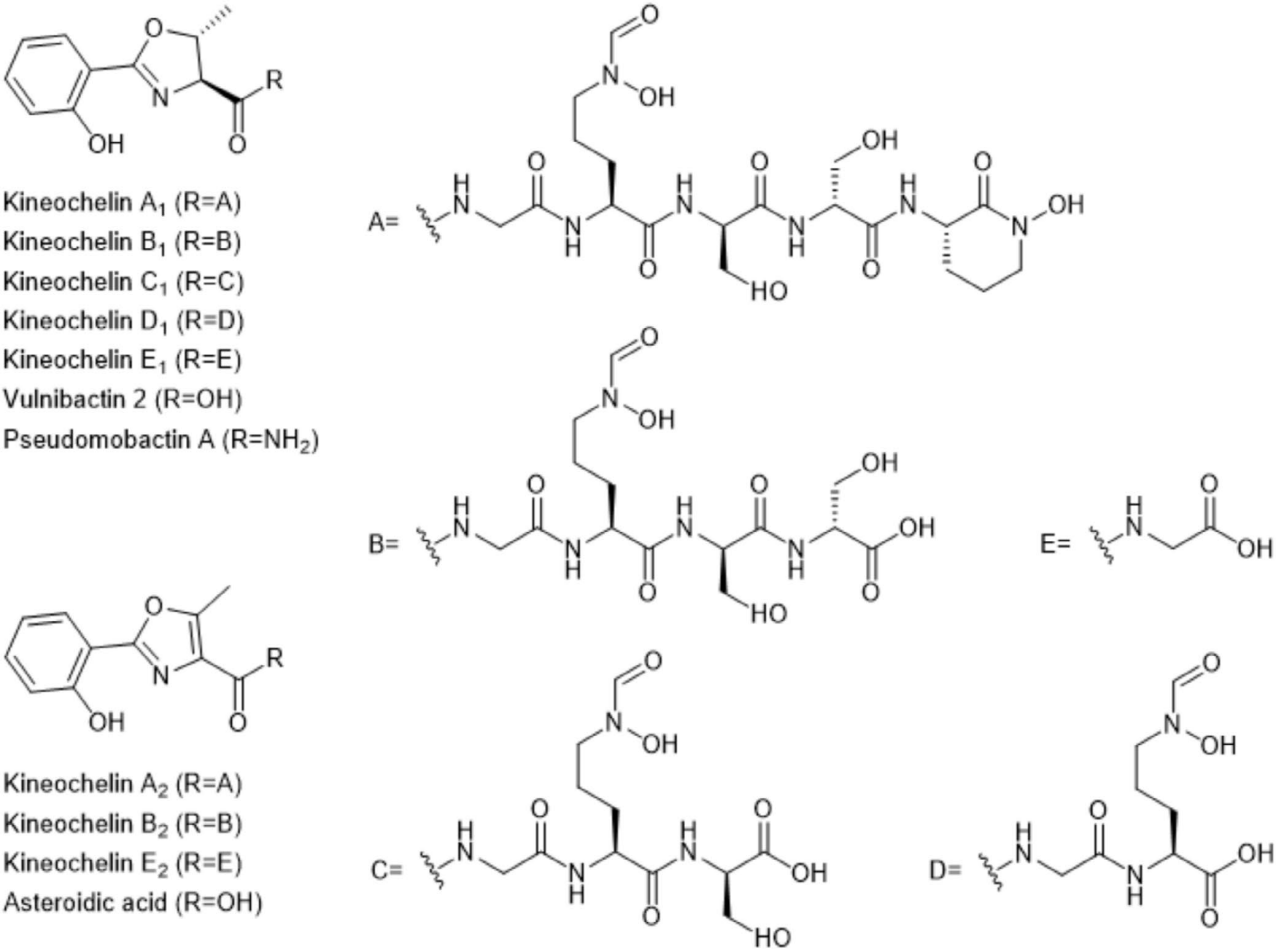
Proposed naming and structures of the kineochelin group and the known shunt products. Shown are kineochelin congeners and known shunt products vulnibactin 2, pseudomobactin A and asteroidic acid. Only the structures of selected congeners, kineochelin A_1_ and E_1_, were elucidated by comprehensive NMR and Marfey's analysis in this study, while for all other congeners they remain tentative.

Initial prioritization of low‐abundance chlorinated metabolites likely associated with the halogenase‐containing BGC 2.18 did not yield sufficient material for characterization despite targeted activation efforts (Supplementary Note). However, one of the resulting genetically engineered strains unexpectedly showed a complete loss of antimicrobial activity that was observed for the wild‐type strain (Figure S2). This prompted comparative metabolomic analysis, which revealed the absence of a large group of structurally related congeners in the extract from the non‐active mutant. These secondary metabolites covered a wide range of monoisotopic masses from 219 to 722 Da (Figure S2 and Table S1). The three smallest congeners produced by the wild type were tentatively identified as asteroidic acid (Steinmetz et al. 2022), pseudomobactin A (Oluwabusola et al. 2021) and vulnibactin 2 (Okujo et al. 1994). These compounds are small siderophores biosynthesized from salicylic acid and threonine. The congeners with higher masses shared with them either the 2‐(2‐hydroxyphenyl)‐5‐methyl‐4,5‐dihydrooxazole‐4‐carbonyl or 2‐(2‐hydroxyphenyl)‐5‐methyl‐4,5‐oxazole‐4‐carbonyl subunit, but differed in mass by increments matching to amino acids such as glycine, serine and *N*‐hydroxy‐ornithine derivatives, the latter typically found in hydroxamate siderophores (Figure 2). The tentative structures derived from the LC–MS data did not match any known compounds in available databases. We provisionally designated this previously unrecognized metabolite group as kineochelins and assigned names to the individual putative congeners based on the tentative structures derived from the MS/MS spectra, with the letters A‐E referring to the number of amino acids attached to the vulnibactin 2 core from 5 to 1, respectively, and the subscript index 1 or 2 referring to the oxidation sate of the oxazole‐ring (Figure 2). In conclusion, our ur integrative activity‐guided, metabolomic and genome‐mining approach revealed a structurally distinct group of metabolites.

### 2.2 Structural Analysis of Representative Kineochelin Congeners

The kineochelins were enriched from the SM17 medium supernatant by adsorption to HP‐20 resin, from which they were extracted with *n*‐butanol. The dried extract was then fractionated by flash chromatography (FC) and pooled into 21 fractions according to results from analysis with ultra‐high‐performance liquid chromatography coupled to photodiode‐array and evaporative light‐scattering detection (UHPLC‐PDA‐ELSD).

**FIGURE 3:**
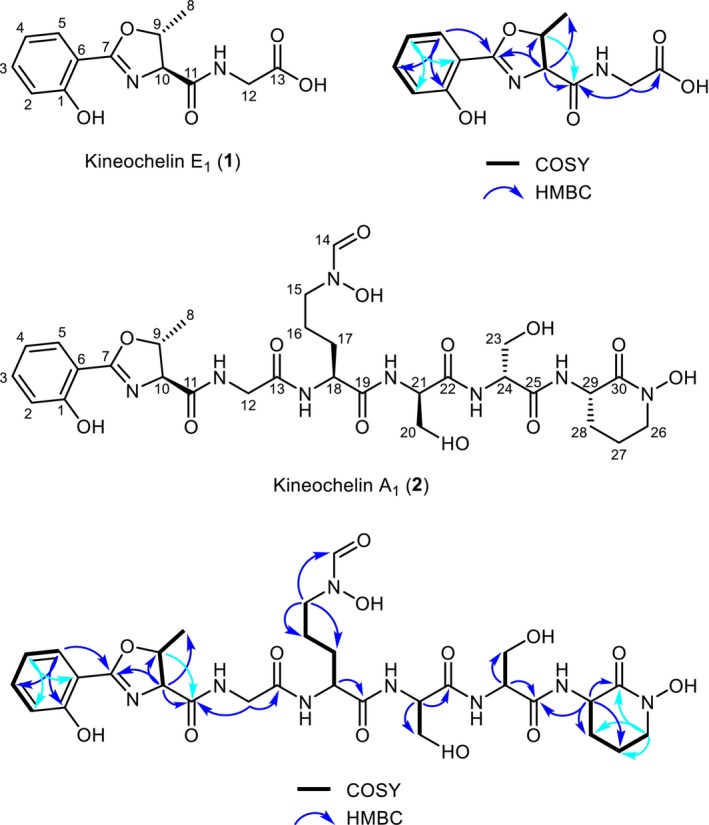
Structure of kineochelin E_1_ (**1**) and kineochelin A1 (**2**). Atom numbering and ^1^H‐^1^H COSY and key ^1^H‐^13^C HMBC correlations are shown.

Analysis by UHPLC‐ELSD as well as by UHPLC–MS demonstrated fraction 17 (2.0 mg) to contain kineochelin E_1_ (**1**) of high purity (Figure S3). This compound, which was assigned the molecular formula C_13_H_14_N_2_O_5_ (Table S1), was subsequently analysed by 1D and 2D NMR spectroscopy. Signals of 13 carbon and 11 non‐exchanging hydrogen atoms were detected, while three fast‐exchanging hydrogens could not be observed in MeOH‐d_4_ (Table 2). The ^1^H signals belonged to three spin systems, one made up of four aromatic methines (H‐2 to H‐5), the second consisting of two aliphatic methines (H‐9 and H‐10) and a methyl group (H‐8), and finally an isolated methylene group (H‐12a and H‐12b). A thorough interpretation of the 1D and 2D NMR spectra (Figure 3 and Figures S4–S8) confirmed that the former two spin systems are indeed characteristic for the 2‐(2‐hydroxyphenyl)‐5‐methyl‐4,5‐dihydrooxazole‐4‐carbonyl substructure that was expected from the LC–MS data. The observed chemical shifts of C‐1 to C‐11 and H‐2 to H‐10 as well as the measured coupling constants of the latter match very well to the previously reported data of pseudomobactin A (Oluwabusola et al. 2021) (Table 2), amychelin C (Lee et al. 2022) and the catenulobactins A and B (Hoshino et al. 2018). The heteronuclear multiple‐bond correlations (HMBC) of the methylene hydrogens (H‐12a and H‐12b) to the carbonyl C‐11 prove that a glycine is attached to the C‐terminus of the oxazoline moiety via an amide bond.

**TABLE 2:** ^1^H (600 MHz) and ^13^C NMR data (151 MHz) of kineochelin E_1_ (**1**) and kineochelin A_1_ (**2**) in CD_3_OD in comparison with literature data for pseudomobactin A (*δ* in ppm).

Position	Kineochelin E_1_ (**1**)		Kineochelin A_1_ (**2**)		Pseudomobactin A^a^	
	δ_H_ (J in Hz)	δ_C_, type	δ_H_ (J in Hz)	δ_C_, type	δ_H_ (J in Hz)	δ_C_, type
1	—	161.3, C	—	161.2, C	—	161.1, C
2	6.96, m, ov	117.9, CH	6.95, m, br, ov	117.9, CH	6.96, d (8.3)	117.7, CH
3	7.41, m	135.2, CH	7.41, t (7.8), br	135.2, CH	7.41, td (8.3, 1.7)	135.0, CH
4	6.90, m, ov	120.1, CH	6.90, m, br, ov	120.1, CH	6.90, td (7.4, 1.7)	119.9, CH
5	7.69, dd (7.9, 1.7)	129.7, CH	7.68, m, br	129.6, CH	7.68, dd (7.4, 1.7)	129.5, CH
6	—	111.8, C	—	111.8, C	—	111.6, C
7	—	168.3, C	—	168.0, C	—	167.8, C
8	1.57, d (6.3)	21.6, CH_3_	1.56, m, ov	21.6, CH_3_	1.57, d (6.3)	21.4, CH_3_
9	4.91, dq (7.5, 6.3)	80.8, CH	4.92, m, br	80.5, CH	4.90, qd (7.3, 6.3)	80.6, CH
10	4.51, d (7.5)	75.9, CH	4.55, d (7.5), br, ov	75.9, CH	4.46, d (7.3)	75.5, CH
11	—	173.3, C	—	173.9, C	—	175.6, C
12a	3.93, d (17.0), ov	43.0, CH_2_	3.83–4.16, m, ov	43.4, CH_2_	—	—
12b	3.87, d (17.5)				—	—
13	—	173.8^b^, C	—	173.0, C	—	—
14	—	—	8.28, s, br	159.6, CH	—	—
15	—	—	3.53, m, ov	50.9, CH_2_	—	—
16	—	—	1.71^c^, m, ov	24.1, CH_2_	—	—
17a	—	—	1.80^c^, m, ov	28.8, CH_2_, ov	—	—
17b	—	—	1.71^c^, m, ov		—	—
18	—	—	4.34, m, ov	55.2, CH	—	—
19	—	—	—	175.7, C	—	—
20a	—	—	3.92^c^, m, ov	62.3, CH_2_	—	—
20b	—	—	3.81–3.89^c^, m, ov		—	—
21	—	—	4.27, m	58.6, CH	—	—
22	—	—	—	172.4, C	—	—
23a	—	—	4.00, m, ov	62.8, CH_2_	—	—
23b	—	—	3.81–3.89^c^, m, ov		—	—
24	—	—	4.41, m	58.1, CH	—	—
25	—	—	—	172.1, C	—	—
26a	—	—	3.63, m, ov	52.6, CH_2_	—	—
26b	—	—	3.59, m, ov		—	—
27a	—	—	2.04, m, ov	21.5, CH_2_	—	—
27b	—	—	1.94, m, ov		—	—
28a	—	—	2.02^c^, m, ov	28.8, CH_2_, ov	—	—
28b	—	—	1.80^c^, m, ov		—	—
29	—	—	4.59, dd (9.8, 5.2)	51.5, CH	—	—
30	—	—	—	167.0, C	—	—

^a^ Values obtained from Oluwabusola et al. (2021).

^b^ Value determined from HMBC spectrum.

^c^ Value determined from HSQC spectrum.

Although the excellent agreement of the NMR data with those of pseudomobactin A (Table 2) suggests that kineochelin E_1_ is also derived from L‐Thr, the configuration around the two chiral centres C‐9 and C‐10 could not be established with certainty from the NMR data. Furthermore, a second set of signals was observed in the NMR data in a ratio of approximately 3:5 to the one described above (Table S2). This set of signals was hypothesized to belong to a stereoisomer of **1** obtained by configurational inversion at one of the chiral centres. The absolute configuration was determined by Marfey's analysis after hydrolysis of 410 μg of kineochelin E_1_ (Oluwabusola et al. 2021). Comparison with all four possible stereoisomers L‐Thr, D‐Thr, D‐allo‐Thr and L‐allo‐Thr by LC–MS proved that kineochelin E_1_ is indeed derived from L‐Thr and that there is no other stereoisomer present in the sample (Figure S9). Kineochelin E_1_ is thus characterized as the new compound {[(4*S*,5*R*)‐2‐(2‐hydroxyphenyl)‐5‐methyl‐4,5‐dihydro‐1,3‐oxazole‐4‐carbonyl]amino}acetic acid (Figure 3).

Fraction 11 (5.8 mg) contained kineochelin A_1_ (**2**), the largest and most abundant detected kineochelin congener, with an estimated purity of ~80% according to the ELSD data (Figure S10). Kineochelin A_1_ has a sum formula of C_30_H_42_N_8_O_13_ (Table S1) and was hypothesized to contain kineochelin E_1_ (**1**) as N‐terminal substructure. This was confirmed by comparison of the NMR data of fraction 11 (Figures S11–S15) with those of fraction 17. The signals of the 2‐(2‐hydroxyphenyl)‐5‐methyl‐4,5‐dihydro‐1,3‐oxazole‐4‐carbonyl substructure of **2** were nearly identical to those in **1** (Table 2). The signals of the adjacent glycine were slightly more affected by the chain extension, but also easily assigned, despite considerable overlap of various signals in the much more complex ^1^H NMR spectrum of **2**. Based on the thorough interpretation of the MS/MS spectra of all detected putative kineochelin congeners (Figures S16–S26), the following units were sequentially attached to kineochelin E_1_ scaffold, yielding the higher‐order congeners kineochelin D_1_ to A_1_, respectively: C_6_H_10_N_2_O_3_, C_3_H_5_NO_2_, C_3_H_5_NO_2_ and C_5_H_8_N_2_O. The third amino acid after L‐Thr and Gly was found to be *N* ^5^‐hydroxy‐*N* ^5^‐formyl‐ornithine, a non‐proteinogenic amino acid typical for hydroxamate siderophores. The identification of this amino acid was supported by the characteristic signals of the formyl‐group (H‐14 and C‐14) in the 1D and 2D NMR spectra. The next two units were found to be Ser residues, followed by a cyclized *N* ^5^‐hydroxy‐ornithine as final C‐terminal amino acid residue. The latter could either be piperazic acid, as in the cahuitamycins (Park et al. 2016), or result from amide bond formation between the C‐terminus and the side‐chain hydroxylamine group, as in the amychelins and gobichelins (Seyedsayamdost et al. 2011; Chen et al. 2012). An HMBC cross signal of H‐26 to the carbonyl C‐30 clearly proves the cyclic amide (Figure 3), which leads to the complete planar structure. To establish the stereochemistry, 750 μg of **2** were hydrolysed for Marfey's analysis and the reaction products compared with commercial standards. Only L‐Thr, L‐Orn and D‐Ser were detected, thus fully establishing the absolute configuration of kineochelin A_1_ (Figure S27).

Along with the kineochelins, two 2,5‐diketopiperazines were isolated from fractions 2 and 3 (4.6 mg) and fraction 5 (1.2 mg). They were identified as cyclo(Hyp‐Leu) and cyclo(Hyp‐Phe), respectively, by interpretation of the HRESIMS and NMR data and by comparison to literature values (Figures S28–S36 and Tables S1 and S3) (Xiang et al. 2020; Harish and Periasamy 2017). However, they were not further studied regarding stereochemistry or biological activity.

### 2.3 Biosynthetic Gene Cluster for Kineochelin Production

**FIGURE 4:**
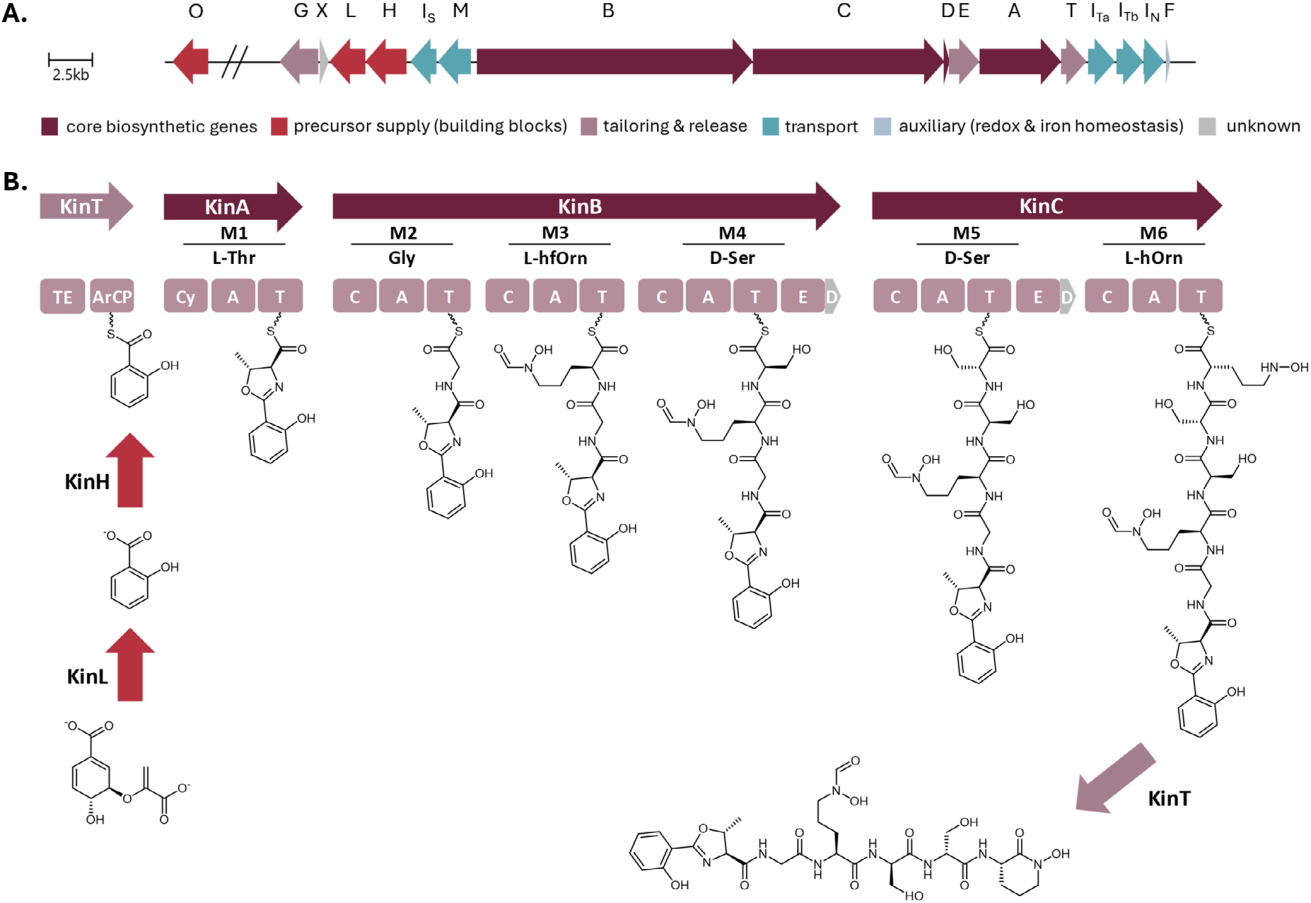
Gene cluster and proposed model for kineochelin biosynthesis. (A.) Kineochelin biosynthetic genes cluster (*kin*) with genes *kinA*‐*kinX*. Gene colour‐coding is indicated in the legend. (B) Proposed biosynthetic model for the kineochelins. Domain abbreviations: C, condensation; A, adenylation; T, thiolation domain (peptidyl carrier protein); E, epimerization; TE, thioesterase (product‐release by macrocyclization or hydrolysis); D, docking domain; ArCP, aryl carrier protein.

While several BGCs in the UV203 genome contain NRPS modules, only BGC 2.11 could be matched to the proposed biosynthesis of the kineochelins (Table 1). It harbours three core genes, *kinA, kinB* and *kinC*, encoding a total of six NRP modules, two of which contain epimerization‐domains (Figure 4A,B). Furthermore, it encodes a salicylate synthase (*kinL*) and displays ~38% homology to the *gob* BGC that is responsible for the biosynthesis of the structurally related mixed‐ligand siderophores gobichelin A and B (Chen et al. 2012). The cluster is hereafter referred to as the *kin* cluster.

In the proposed biosynthesis, the aromatic starter unit is derived from chorismate, which is converted into salicylate via a standalone salicylate synthase KinL, homologous to Ccb3 from the celestinticine biosynthetic pathway (59% identity) (Janata et al. 2015) and AmcL from *Streptomyces* sp. AA4 (49.4% identity) in the amychelin BGC (Seyedsayamdost et al. 2011) (Table S4). The resulting salicylate is activated by the salicylate‐AMP ligase KinH, which shares 58.5% identity with AmcH from the amychelin cluster. In contrast to GobJ, AmcG and CahA, the initiating NRPSs in the biosyntheses of the gobichelins, amychelins and cahuitamycins, respectively, no aryl carrier protein (ArCP) domain was detected in the corresponding NRPS KinA. We assume that KinH instead loads the activated salicylate to the ArCP domain encoded in the didomain protein KinT. KinA activates and loads L‐Thr onto its peptidyl carrier protein (PCP) domain. Its N‐terminal cyclization (Cy) domain accepts KinH as the salicylate donor and catalyses the cyclodehydration with L‐Thr to give the initiating 2‐(2‐hydroxyphenyl)‐5‐methyl‐4,5‐dihydrooxazole‐4‐carbonyl unit. This intermediate is then passed to the downstream three‐module NRPS KinB (Figure 4B), which extends the chain by a Gly, an *N* ^5^‐hydroxy‐*N* ^5^‐formyl‐L‐ornithine and a D‐Ser residue, the latter via epimerization of L‐Ser. The growing chain is then passed to the two‐module NRPS KinC, which adds another D‐Ser and an *N* ^5^‐hydroxy‐L‐ornithine (*N*‐OH‐L‐Orn) residue. Like in the amychelin biosynthetic pathway, the final NRPS module in the kineochelin assembly lacks an associated *C*‐terminal thioesterase domain (Seyedsayamdost et al. 2011). Instead, a standalone ArCP–TE didomain protein (KinT) is encoded within the cluster, which we hypothesize to be responsible for the release of kineochelin A_1_ via cyclization of the C‐terminal *N*‐OH‐L‐Orn.

**FIGURE 5:**
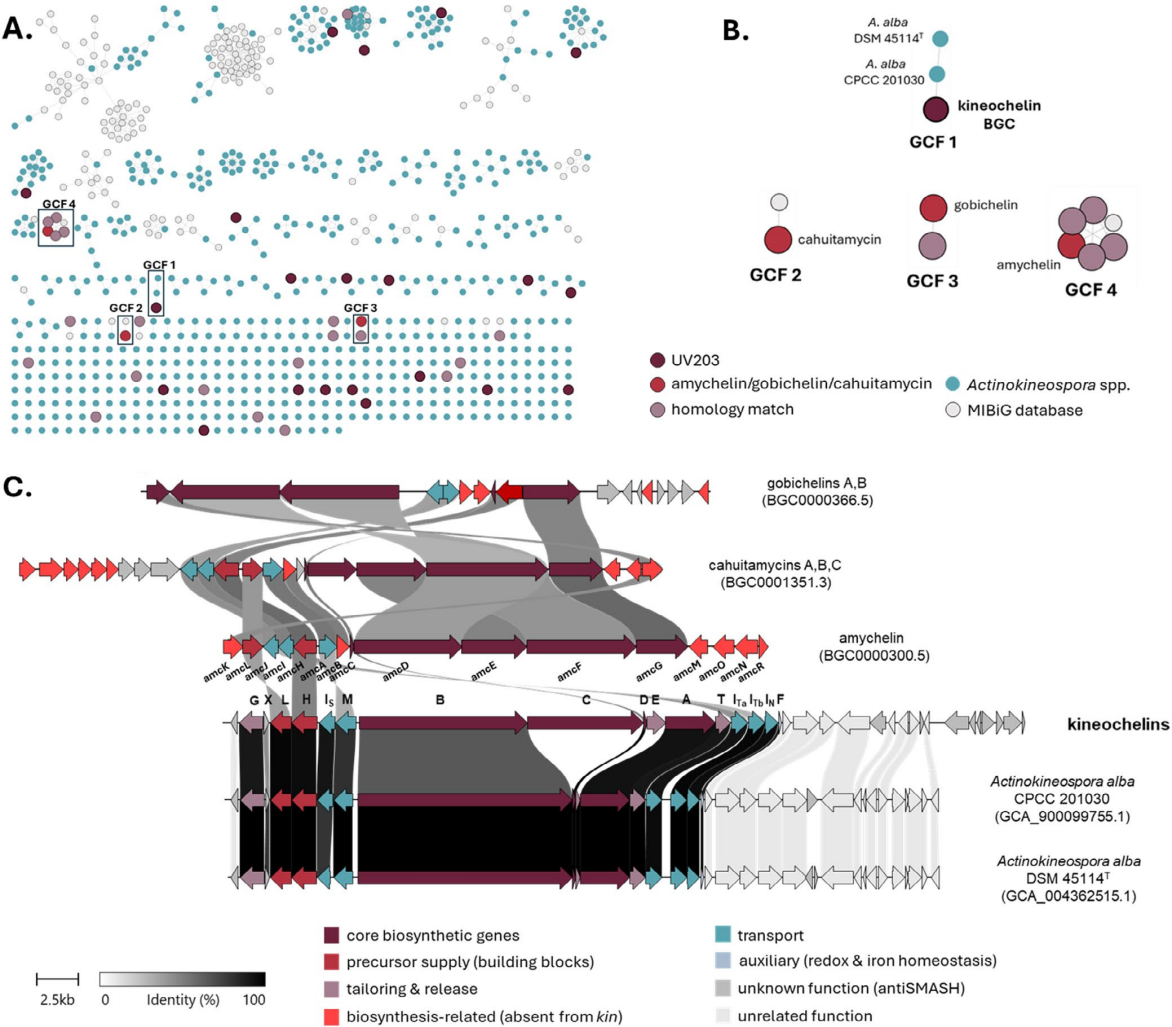
Kineochelin BGCs in *Actinokineospora alba* and strain UV203 represent a new gene cluster family. (A) BiG‐SCAPE (Navarro‐Muñoz et al. 2020) sequence similarity network (SSN, cutoff 0.4) constructed from BGCs predicted in strain UV203 and all available *Actinokineospora* spp., together with the MIBiG database and three biosynthetically related siderophores. Groups of BGCs are colour‐coded as indicated in the legend. (B) Enlarged view of the gene cluster families (GCFs) containing the kineochelin, amychelin, cahuitamycin and gobichelin BGCs. (C) Clinker (Gilchrist and Chooi 2021) comparison within kineochelin cluster (member of GCF 1), with the amychelin, gobichelin and cahuitamycin BGCs. Links between homologous genes are displayed according to percentage identity (see identity scale bar), with only links representing ≥ 40% amino acid identity shown. Gene arrows are drawn to scale, oriented by transcriptional direction, and coloured by functional category.

Comparative network analysis with BiG‐SCAPE placed the *kin* cluster of strain UV203 in a separate biosynthetic gene cluster family (GCF) with two uncharacterized BGCs from *Actinokineospora alba* strains (Figure 5A,B). To further contextualize the *kin* cluster, we manually included additional BGCs whose core biosynthetic genes showed the highest similarity to *kin* genes, as identified by NCBI protein BLAST searches and antiSMASH analysis (Tables S5 and S6). Clinker‐based synteny comparisons revealed that full‐length (global) alignments of core biosynthetic proteins between the *kin* cluster and related clusters did not exceed 40% amino acid identity (Gilchrist and Chooi 2021) (Figure 5C). In contrast, local BLAST‐based alignments identified > 40% identity for several homologous proteins (Altschul et al. 1990), indicating detectable gene‐level similarity restricted to specific domains or regions (Mount 2004) (Table S4).

### 2.4 Iron‐Dependent Expression Links the Kin BGC to Kineochelin Production

To confirm the role of the *kin* cluster in kineochelin biosynthesis, we initially attempted targeted gene inactivation using a single‐gene knockout strategy. For both *kinA* and *kinB*, two knockout constructs containing 600 and 1000 bp intragenic gene regions, respectively, failed to produce exconjugants despite repeated conjugation attempts, whereas the control plasmid pSET152 integrated successfully, confirming the genetic tractability of *Actinokineospora* sp. UV203. Given these limitations, we shifted to a transcriptomics‐based approach to assess the expression of the *kin* biosynthetic genes under iron‐modulated conditions.

**FIGURE 6:**
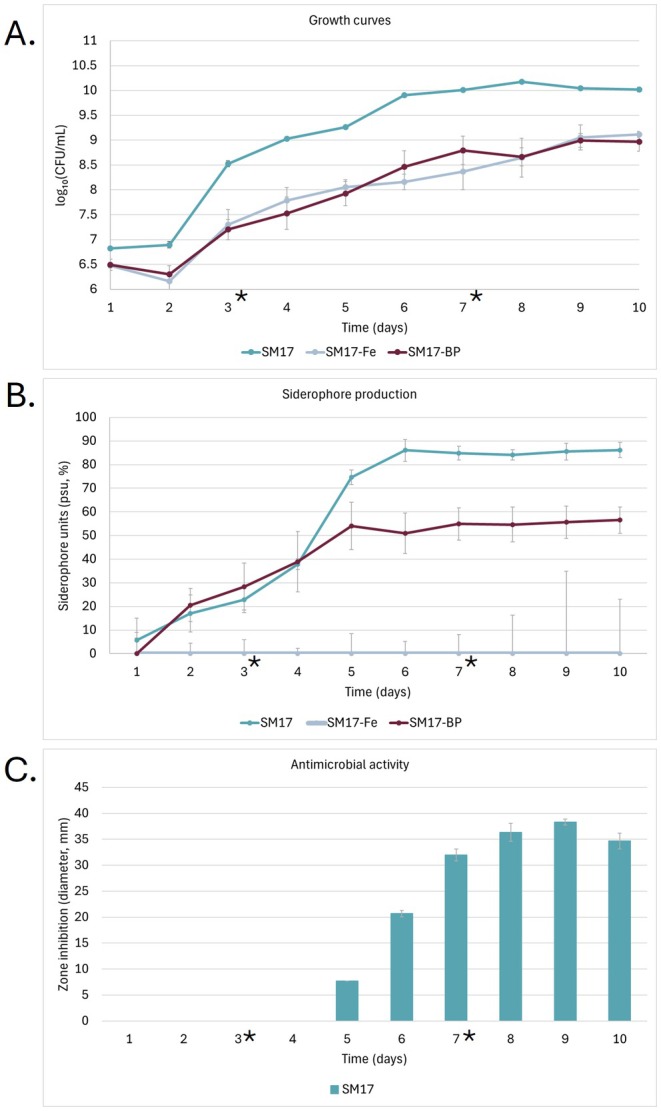
Growth, siderophore production and antimicrobial activity of *Actinokineospora* sp. UV203 under three iron regimes. (A) Growth curves (log_10_ CFU/mL) in unmodified SM17 medium (SM17), supplemented with FeCl₃ (SM17‐Fe, iron‐rich) or with the iron‐chelator 2,2′‐bipyridine (SM17‐BP, iron‐limited). Quantification by area under the growth curve (AUC) confirmed this pattern: SM17 = 91.95; SM17‐Fe = 80.47; SM17‐BP = 80.38. A one‐way ANOVA detected significant differences among treatments (*F*_2,6_ = 18.85, *p* = 0.0026). Tukey's HSD (HSD = 6.65) indicated significant reductions in growth in both SM17‐Fe (Δ = 11.48) and SM17‐BP (Δ = 11.57) relative to unamended SM17, while growth in SM17‐Fe and SM17‐BP did not differ (Δ = 0.09). (B) Overall siderophore production in culture supernatants measured as percent siderophore units (PSU) by the Chrome Azurol S (CAS)‐shuttle assay. SM17‐Fe showed no detectable siderophores (CAS values ≤ 0 plotted as 0). (C) Antimicrobial activity of crude extracts is shown as bars (left y‐axis), representing inhibition zone diameters (mm) against *Micrococcus luteus* CCM 169^T^. Antimicrobial activity was detected only for the SM17 medium; no activity was observed for SM17‐Fe or SM17‐BP. Error bars indicate standard deviations (SD) from three biological replicates. Asterisks indicate sampling days for transcriptome analyses.

To select suitable conditions for comparative transcriptome analysis, we initially monitored growth, siderophore production and antimicrobial activity of the wild‐type strain during 10‐day cultivation in three variants of SM17 medium: unmodified (SM17), supplemented with FeCl_3_ (SM17‐Fe, iron‐rich) or with the iron‐chelator 2,2′‐bipyridine (SM17‐BP, iron‐limited). In preliminary tests, both 100 and 200 μM FeCl_3_ suppressed siderophore production, with stronger inhibition at 200 μM; this concentration was therefore used as the iron‐rich condition. Growth differed significantly, depending on the media used (Figure 6A). Growth in SM17 showed the highest yield, whereas both FeCl_3_ and bipyridine supplementation reduced the growth.

Bipyridine supplementation did not increase siderophore production relative to SM17, indicating that the kineochelin pathway is already derepressed under the baseline medium conditions (Figure 6B). Across the 10‐day time course for SM17, siderophore titre rose sharply between Days 4 and 6 (Figure 6B), coinciding with the first detectable antimicrobial activity (Figure 6C). Siderophore levels peaked at Day 6, and antimicrobial activity at Day 8, after which both remained stable.

Based on these dynamics, transcriptome sequencing was performed at two time points during growth: Day 3, capturing the onset of SM production and Day 7, corresponding to peak activity. We performed comparative transcriptome analysis of strain UV203 grown in unamended and FeCl_3_‐supplemented SM17, as iron‐rich conditions showed almost complete repression of siderophore activity.

**FIGURE 7:**
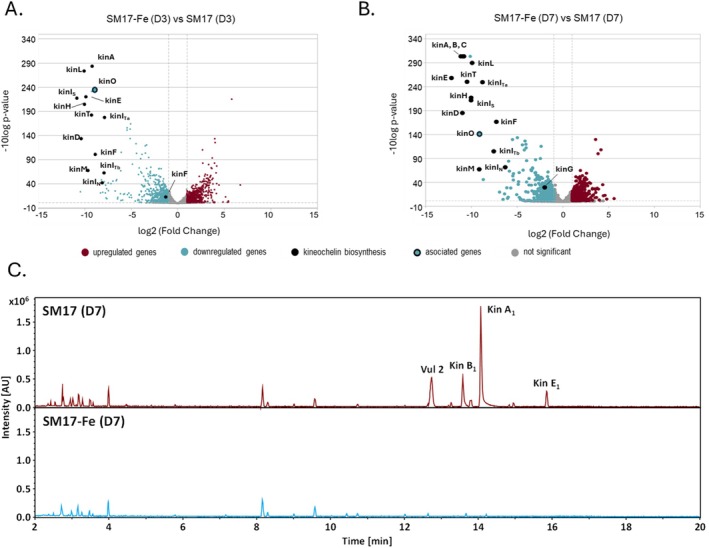
Comparative transcriptomics and metabolomics of *Actinokineospora* sp. UV203 under siderophore‐producing and siderophore‐depleted growth conditions. Volcano plots for the indicated pairwise comparisons. (A) SM17 supplemented with 200 μM FeCl_3_ versus SM17 (control) at Day 3. (B) SM17 supplemented with 200 μM FeCl₃ versus SM17 (control) at Day 7. Each point represents a gene (x‐axis: Log_2_ fold change; y‐axis: −log_10_ [FDR]); Vertical dashed lines indicate the log_2_ fold‐change threshold (|log_2_FC| ≥ 1), and the horizontal dashed line marks the FDR significance cutoff (FDR < 0.05; −log_10_FDR = 1.3). PCA and volcano plots were generated based on normalized counts from DESeq2 (Love et al. 2014). (C) Representative base peak chromatograms of the culture extracts of *Actinokineospora* sp. UV203 grown in SM17 supplemented with 200 μM FeCl_3_ versus SM17 (control) at Day 7.

Principal component analysis (PCA) of the transcriptome data demonstrated clear separation of samples by both the conditions and time points, with tight clustering of biological replicates (Figure S37). Comparative transcriptomics showed that genes in the *kin* cluster were co‐transcribed and significantly upregulated under siderophore‐producing conditions (SM17 control) compared to iron‐replete conditions (SM17 with 200 μM FeCl_3_) at both time points (Figure 7A,B). Differential transcription of *kin* genes was consistent with selective production of kineochelin congeners only under iron‐limited conditions (Figure 7C, Figure S38). We identified an additional co‐regulated gene outside the *kin* cluster, annotated as a lysine/ornithine N‐oxygenase, and designated it *kinO* (Figure 7A,B).

### 2.5 Kineochelin‐Enriched Fractions Exhibit Metal‐Binding Activity and Iron‐Dependent Growth‐Inhibitory Effects

**FIGURE 8:**
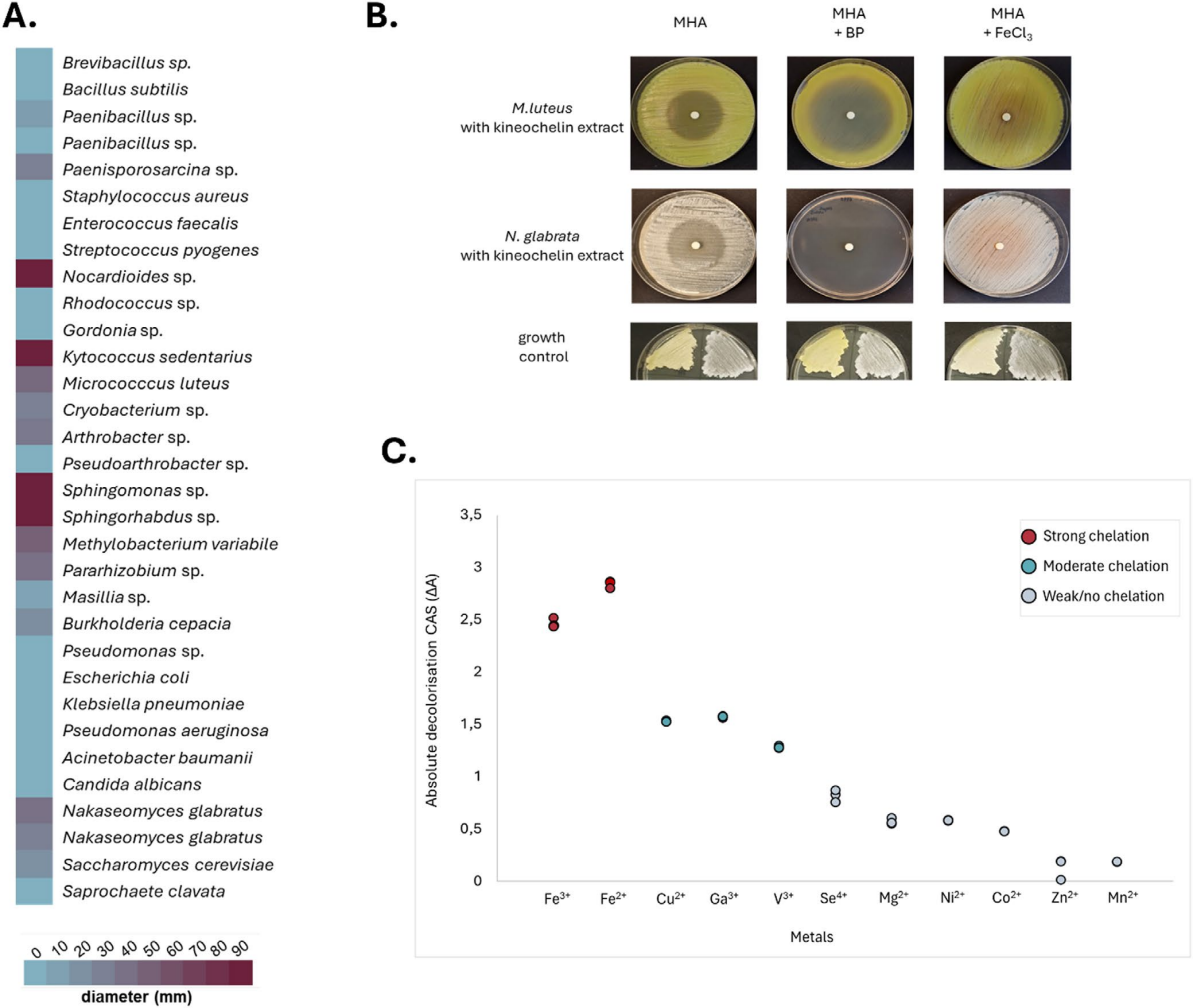
Iron‐dependent growth‐inhibitory effects and metal‐binding properties of UV203‐derived extracts. (A) Growth‐inhibitory effects of crude extracts from *Actinokineospora* sp. UV203 against Antarctic environmental bacteria, the indicator strain *M. luteus* CCM 169ᵀ and clinically relevant microbial strains. (B) Iron‐dependent modulation of growth inhibition by a pre‐purified, kineochelin‐enriched extract (Figure S39). Assays were performed on Mueller–Hinton agar (MHA), MHA supplemented with 100 μM 2,2′‐bipyridine (MHA + BP) or MHA supplemented with 200 μM FeCl_3_ (MHA + FeCl_3_). For *N. glabratus* , media were additionally supplemented with 2% glucose. (C) Metal‐binding activity of a pre‐purified, kineochelin‐enriched fraction measured using the Chrome Azurol S (CAS) shuttle assay. Absolute CAS decolorization (ΔA) values for individual metals are shown. Points represent technical replicates (*n* = 3); colours indicate relative binding strength normalized to Fe^3+^.

Preliminary bioactivity screening of crude extracts from *Actinokineospora* sp. UV203 against clinically relevant bacteria, yeasts and other isolates from Antarctic soils revealed growth‐inhibitory effects, with the most pronounced inhibition observed against the strains from the same environment (Figure 8A, Table S7). In addition, inhibitory activity against yeasts was detected, particularly *Nakaseomyces glabratus* and *Saccharomyces cerevisiae* isolates as well as against the bacterial indicator strain *Micrococcus luteus* CCM 169^T^ (Figure 8A, Table S7). Follow‐up testing with a pre‐purified kineochelin‐enriched fraction, containing predominantly kineochelin A1, B1 and E1 with minor contributions from the co‐produced siderophore vulnibactin 2 and trace‐level co‐occurring metabolites, including pseudomobactin A and asteroic acid (Figure S39), confirmed growth inhibition of both *M. luteus* CCM 169^T^ and yeasts in agar diffusion assays (Table S8). However, due to limited extract availability and the greater clinical relevance of the yeast targets, subsequent minimum inhibitory concentration (MIC) and minimum fungicidal concentration (MFC) determinations were restricted to antifungal assays. Inhibition was observed only at relatively high concentrations, with MIC values of 0.5 mg/mL for *Nakaseomyces* strains and 2.5 mg/mL for *S. cerevisiae* , and corresponding MFC values of 2.5 and 5 mg/mL, respectively (Table S8), indicating modest antifungal potency under the conditions tested. Because these concentrations exceeded the amounts obtainable for individual purified kineochelin congeners, antifungal testing was performed using pre‐purified kineochelin‐enriched fractions.

To assess the influence of iron availability on growth inhibition, sensitive yeasts and the bacterial indicator strain *M. luteus* CCM 169ᵀ were assayed on media supplemented either with excess iron or with the iron chelator 2,2′‐bipyridine (Figure 8B). Iron supplementation markedly reduced growth inhibition, whereas iron depletion in the presence of pre‐purified kineochelin‐enriched fractions resulted in a complete growth arrest of sensitive yeasts. A comparable iron‐dependent response was observed for *M. luteus* CCM 169^T^ (Figure 8B).

To assess the metal‐binding properties associated with kineochelin production, we applied a modified Chrome Azurol S (CAS) assay to both whole culture supernatants of *Actinokineospora* sp. UV203 and a pre‐purified kineochelin‐enriched fraction (Figure 8C; Figure S40). Both sample types showed a consistent hierarchy of metal‐binding activity, with strongest responses toward ferric and ferrous iron, intermediate activity for Cu^2+^, Ga^3+^ and V^3+^, and weak or near‐baseline responses for other tested metals. The comparable profiles observed for the pre‐purified kineochelin‐enriched fraction and culture supernatant support iron‐selective chelation as a major activity associated with kineochelins, although contributions from co‐occurring metabolites in non‐purified samples cannot be excluded.

We further assessed the antiproliferative activity of the pre‐purified kineochelin fraction, tested as two independently handled aliquots (E1 and E2), against a panel of seven human cancer cell lines representing distinct tissue origins (A549, U‐87, PaTu 8902, Jurkat, HCT116, A2058 and MDA‐MB‐231) using the resazurin assay. The calculated IC_50_ values are presented in Table S9. Within the tested concentration range of 10–750 μg/mL, aliquot E1 exhibited inhibitory effect on the metabolic activity of A549 (545 ± 32 μg/mL) and A2058 cells (639 ± 32 μg/mL), whereas aliquot E2 demonstrated inhibitory activity against A549 (715 ± 16 μg/mL), U‐87 (742 ± 27 μg/mL) and HCT116 (494 ± 48 μg/mL). The partially overlapping activity profiles observed for E1 and E2 are consistent with expected variability between independently handled aliquots of the same pre‐purified fraction.

## 3 Discussion

In this study, we investigated the biosynthetic, chemical and functional basis of siderophore production in *Actinokineospora* sp. UV203, a phylogenetically distinct actinomycete isolated from Antarctic soils. Although UV203 exhibited relatively narrow‐spectrum antimicrobial activity in preliminary testing, predominantly against environmental strains, we prioritized it for untargeted secondary metabolome profiling based on the apparent novelty of its genomic biosynthetic potential and its distinct taxonomic placement. In the context of nutrient‐ and iron‐limited Antarctic soil ecosystems, such activity may be consistent with localized microbial competitive interactions rather than broad‐spectrum antagonism (Cary et al. 2010). This strategy proved effective, leading to the identification of kineochelins as the dominant SMs produced under iron‐limited conditions.

Although other unexplored metabolite features were detected in UV203, including low‐abundance chlorinated compounds, their production remained insufficient for characterization and they warrant future investigation. In contrast, subsequent comparative metabolomic analyses revealed a distinct group of metabolites whose production was unexpectedly lost in an engineered regulatory mutant, and whose disappearance coincided with the loss of antimicrobial activity. The tentative structures derived from the LC–MS data did not match any known compounds in public databases. The absence of database matches, together with the presence of shared mixed‐ligand substructures, suggested that kineochelins represent a previously unrecognized group of siderophores, potentially related to other mixed‐ligand metallophores such as amychelins (Seyedsayamdost et al. 2011) and gobichelins (Chen et al. 2012). Since LC–MS analyses indicated that kineochelins are major metabolites of *Actinokineospora* sp. UV203 under siderophore‐producing conditions, and their loss correlated with the disappearance of antimicrobial activity, we proceeded to isolate and structurally characterize representative congeners of this siderophore group.

Genome mining and comparative biosynthetic analyses identified the *kin* biosynthetic gene cluster as the most plausible candidate responsible for kineochelin production. Although the *kin* cluster shares biosynthetic logic with pathways directing the biosynthesis of amychelins, gobichelins and cahuitamycins (Park et al. 2016; Seyedsayamdost et al. 2011; Chen et al. 2012), it did not cluster with these pathways in BiG‐SCAPE analyses, even under permissive similarity thresholds. This separation, together with clinker‐based synteny comparisons, indicates that the *kin* cluster and related clusters in *A. alba* strains represent a distinct architectural lineage. Notably, the *kin* BGC in strain UV203 differs in gene content and organization from its closest homologues, most prominently by the presence of the *kinC* NRPS module, which is absent from the *A. alba* clusters. These differences are consistent with variation in peptide length and composition and likely contribute to the structural diversity of the resulting siderophores. More distant homology to the *gob*, *amc* and *cah* BGCs, which direct the biosynthesis of gobichelins, amychelins and cahuitamycins (Park et al. 2016; Seyedsayamdost et al. 2011; Chen et al. 2012), respectively, indicates that kineochelin biosynthesis shares selected features with other mixed‐ligand siderophore pathways, despite substantial divergence in gene content and organization.

Attempts to genetically validate the role of the *kin* cluster through targeted gene disruption were unsuccessful, likely reflecting low recombination efficiency associated with single‐crossover insertion or locus‐specific constraints on chromosomal integration in strain UV203 (Kieser 2000). As an alternative, transcriptomic analyses under iron‐modulated conditions provided strong regulatory evidence linking the *kin* cluster to kineochelin production. Expression of *kin* genes was strongly upregulated under siderophore‐producing conditions and repressed under iron‐replete conditions, consistent with canonical siderophore regulation (Miethke and Marahiel 2007; Andrews et al. 2003). Co‐expression of the *kin* genes, together with the identification of the co‐regulated N‐oxygenase gene *kinO*, supports a coordinated biosynthesis. The predicted function of KinO in supplying *N*‐OH‐L‐Orn provides a mechanistic explanation for the incorporation of hydroxamate moieties into most kineochelins and further substantiates the assignment of the *kin* locus as the biosynthetic source of these compounds (Ge and Seah 2006). The production of *N*‐OH‐L‐Orn via external gene is consistent with structurally related mixed‐ligand siderophores, including amychelins and cahuitamycins (Park et al. 2016; Seyedsayamdost et al. 2011). In addition, the absence of a cis‐acting thioesterase domain within the NRPS assembly line, together with the presence of a standalone ArCP–TE didomain protein (KinT) and the observed cyclic amide at the C‐terminus of selected kineochelin congeners, suggests that product release occurs via a trans‐acting thioesterase. We therefore infer that off‐loading proceeds through intramolecular cyclization mediated by KinT, rather than by canonical hydrolytic release (Koglin and Walsh 2009).

Iron availability had a pronounced impact on growth, siderophore production and bioactivity in UV203. Both iron excess and iron limitation impaired growth, likely through distinct physiological mechanisms. Growth inhibition under iron‐rich conditions is consistent with iron‐associated stress and suppression of iron uptake systems reported for other actinomycetes (Cheng et al. 2018; Abanoz‐Seçgin et al. 2023), whereas iron chelation by bipyridine slows the growth by restricting iron‐dependent metabolic processes (Santos et al. 2018). Notably, iron supplementation strongly reduced the growth‐inhibitory activity of kineochelin‐containing fractions, while iron depletion enhanced inhibition, resulting in complete growth arrest of sensitive bacterial indicator strain *M. luteus*. Lower siderophore levels observed at later time points under iron‐chelated conditions likely reflect reduced biomass accumulation due to growth inhibition. Collectively, these observations are compatible with extracellular iron sequestration as the primary mechanism underlying the growth‐inhibitory effects of kineochelin‐enriched fractions and support a potential role for kineochelins in iron competition under iron‐limited conditions such as those characteristic of Antarctic soils (Kramer et al. 2020; Vlček et al. 2018).

The observed selective sensitivity of *N. glabratus* and *S. cerevisiae* , but not *C. albicans* , to kineochelin‐enriched fraction likely reflects fundamental differences in their iron acquisition strategies. While all three species may activate reductive iron assimilation under low‐iron conditions, *S. cerevisiae* relies predominantly on classical reductive iron uptake via surface ferric reduction followed by Fe^2+^ import through the Ftr1/Fet3 system (Ramos‐Alonso et al. 2020; Dancis et al. 1990), whereas *N. glabratus* has a more restricted iron acquisition repertoire, lacking several ferric reductases and depending mainly on ferrous iron and ferritin‐derived sources (Gerwien et al. 2017; Kumar et al. 2019). Both species are therefore limited in their ability to exploit complex or protein‐bound iron sources. In contrast, *C. albicans* possesses a highly versatile and redundant iron uptake network, including reductive assimilation, heme and haemoglobin utilization, transferrin and ferritin extraction, and siderophore uptake systems (Knight et al. 2005; Almeida et al. 2008; Roy and Kornitzer 2019; Kuznets et al. 2014; Heymann et al. 2002). This flexibility enables *C. albicans* to switch efficiently between iron acquisition mechanisms when extracellular iron becomes limiting, thereby buffering intracellular iron homeostasis against siderophore‐mediated depletion (Chen et al. 2011). Collectively, the more constrained iron acquisition strategies of *N. glabratus* and *S. cerevisiae* likely render them more vulnerable to iron sequestration by kineochelins, explaining their selective sensitivity (Ramos‐Alonso et al. 2020; Dancis et al. 1990; Gerwien et al. 2017; Kumar et al. 2019; Knight et al. 2005; Almeida et al. 2008; Roy and Kornitzer 2019; Kuznets et al. 2014; Heymann et al. 2002; Chen et al. 2011; Kosman 2003; Roy et al. 2022).

Consistent with this model, CAS assays of both culture supernatants and the pre‐purified kineochelin‐enriched fraction indicated that the major metal‐chelating activity of UV203 cultures largely mirrored the activity of kineochelin‐enriched fractions and was dominated by iron binding, with limited but reproducible secondary affinities for select non‐iron metals. Such secondary metal‐binding capabilities may become relevant under competitive or metal‐limited environmental conditions but are unlikely to represent the primary biological function of kineochelins (Hider and Kong 2010; Miethke and Marahiel 2007).

Finally, kineochelin‐enriched fractions exhibited only weak antiproliferative activity against human cancer cell lines, with IC_50_ values outside the range typically considered indicative of cytotoxic activity (Boik 2001). The higher IC_50_ values observed for non‐malignant fibroblasts compared to cancer cell lines suggest a degree of selectivity but overall indicate that kineochelins are unlikely to act as potent cytotoxins. Together, these findings reinforce the interpretation that kineochelins likely function as ecological mediators of iron acquisition and competition rather than as broad‐spectrum antimicrobial or cytotoxic agents.

## 4 Conclusion and Perspectives

Here, we report the discovery of a new group of mixed‐ligand siderophores, the kineochelins, from strain UV203, which was isolated from Antarctic soil and represents a potentially novel *Actinokineospora* species. Bioactivity assays using pre‐purified kineochelin‐enriched fractions revealed selective inhibition of opportunistic yeasts (*N. glabratus, S. cerevisiae*), whereas *C. albicans* showed resistance, an observation consistent with the divergent iron acquisition strategies of these yeasts. The activity of kineochelin‐enriched fractions was strongly dependent on iron availability, supporting a mechanism based on siderophore‐mediated iron sequestration. Although antimicrobial activity was observed only at high concentrations, kineochelins may provide a structurally tractable scaffold for future structure–activity studies aimed at understanding how variation in siderophore architecture influences iron acquisition, competitive interactions and microbial community dynamics in iron‐limited extreme environments.

The pronounced iron selectivity and mixed‐ligand architecture of kineochelins highlight broader biotechnological potential. Metallophores with defined metal preferences have attracted interest as tools for manipulating metal availability in engineered microbial systems, metal mobilization and recovery and the study of metal‐dependent microbial interactions. While such applications were not addressed here, the structural features and biosynthetic accessibility of kineochelins may provide a useful basis for future investigations. More broadly, the discovery of kineochelins in an Antarctic bacterium reinforces the value of polar microbiomes as reservoirs of structurally diverse and previously unexplored NPs and highlights the value of integrating ecological and genomic perspectives in natural product discovery.

## 5 Methods

### 5.1 Biological Resources: Isolation, Culture Conditions, Site Description

James Ross Island is part of the James Ross Island group, which comprises four larger islands and several smaller landmasses located near the northernmost tip of the northeastern Antarctic Peninsula (Trinity Peninsula) (Roman et al. 2024). Active layers from James Ross Island are regularly sampled as part of microbiological biodiversity surveys conducted under the Czech Antarctic Research Programme, which has maintained a long‐term biomonitoring initiative in the area since 2007. *Actinokineospora* sp. UV203 was isolated from an active layer sample at a depth of 10 cm below the surface at Abernathy Flats (63°50′47.5″ S, 57°53′23.9″ W) collected in the austral summer of 2022 on James Ross Island.

To selectively cultivate sporulating bacteria, 1 g of the sediment sample was pre‐treated by air‐drying for 48 h, then suspended in 9 mL of sterile saline (0.9%) solution by vortexing. The suspension was incubated in a water bath at 50°C for 5 min (Rego et al. 2019), followed by serial 10‐fold dilutions up to 10^−5^. Aliquots of 100 μL of each dilution were spread onto a range of actinomycete‐selective media, including starch‐casein, starch‐nitrate, International Streptomyces Project (ISP) 2, humic acid–vitamin and Actinomyces agar (Merck, DE) plates (Hayakawa and Nonomura 1987; Shirling and Gottlieb 1966; Waksman 1959; Sapkota et al. 2020). Each medium was supplemented with filter‐sterilized cycloheximide (50 mg/L) and nalidixic acid (20 mg/L) to suppress the growth of fungi and fast‐growing Gram‐negative bacteria (Roy and Kornitzer 2019). The inoculated plates were incubated at 14°C for up to 3 months, during which colony development was regularly monitored, and morphologically distinct isolates were selected for classification and preserved at −80°C in glycerol stocks.

### 5.2 16S rRNA Gene‐Based Identification of Isolates

DNA was isolated using a rapid Chelex‐based extraction protocol (Chelex 100 Resin, Bio‐Rad), and the nearly full‐length 16S rRNA gene was amplified using the 616V and 1492R primer pair (Loy et al. 2002). Initial identification of *Actinokineospora* sp. UV203 was based on the 16S rRNA gene sequence similarity using EZBioCloud (Chalita et al. 2024) and the preliminary taxonomic position was confirmed through phylogenetic analysis using MEGA X (Kumar et al. 2018). Homologous sequences were aligned using MUSCLE (Edgar 2004), and the phylogenetic tree was reconstructed using the maximum likelihood method. Tree robustness was assessed by bootstrap analysis with 1000 replicates.

### 5.3 Whole Genome Sequencing and Genome Assembly

DNA extraction and whole genome sequencing were performed by the Joint Microbiome Facility of the Medical University of Vienna and the University of Vienna under the project number JMF‐2209‐13. High molecular weight DNA was extracted using the Monarch gDNA purification kit (NEB), with the specific protocol for Gram positive bacteria. Pellets were initially treated with an enzymatic cocktail (innuPREP Bacteria Lysis Booster, Innuscreen GmbH) to facilitate chemical cell wall lysis. Samples were diluted and equimolarly barcoded using the SQK‐RBK114.96 (Oxford Nanopore Technologies) with the following protocol modifications: we increased the sample input to 230 ng/sample, +0.5 μL of rapid adaptor to the barcoded library. About 80 fmol of a > 28 Kb library was loaded on a R10.4.1 flowcell (FLO‐PRO114, Oxford Nanopore Technologies) and sequenced for 20 h on a Promethion P2 solo (Oxford Nanopore Technologies, ONT, UK) using Minknow (v. 23.11.7, ONT, UK). Flowcell light shields were used. Reads were basecalled using Dorado server basecaller v7.8.2 (ONT, UK) using super accuracy mode. The nanopore reads were assembled using flye v. 2.9.3 (Kolmogorov et al. 2019), with ‘–nano‐hq’ and polished once with medaka (v. 1.11.3, github.com/nanoporetech/medaka). The model used was ‘r1041_e82_400bps_sup_v4.2.0’. Contigs < 1000 bp were removed and assemblies were quality checked with QUAST v5.2.0 (Gurevich et al. 2013), and CheckM v1.2.2 (Parks et al. 2015). Details on assembled genome are provided in Table S10.

### 5.4 Genomic Analyses

Initial genome‐based taxonomic classification was performed using the TYGS server and Genome‐to‐Genome distance calculator v3.0 (GGDC) using formula 2 (TYGS formula d_4_) digital DNA–DNA hybridization (dDDH) to assess relatedness between UV203 and the type strains of all validly described *Actinokineospora* species (Meier‐Kolthoff et al. 2021; Meier‐Kolthoff and Göker 2019). The ANI values were calculated using FastANI to further evaluate genome‐level similarity (Jain et al. 2018). The dDDH was calculated using formula d_4_ of GGDC. A maximum likelihood phylogenomic tree was constructed based on 92 single‐copy marker proteins identified by the Up‐to‐date bacterial core gene set (UBCG v2) pipeline (Kim et al. 2021). Genome annotation was performed using Bakta v1.11.3 (Schwengers et al. 2021) and the resulting GenBank (.gbk) files were used to predict BGCs via the standalone version of antiSMASH v7.1 (Blin et al. 2023). Functional annotations for genes within each cluster, as well as flanking regions where possible, were refined using BLAST and InterPro, in addition to initial predictions (Sayers et al. 2022; Blum et al. 2025). For sequence similarity network (SSN) analysis, multiple *Actinokineospora* genomes and the closest homologues from the MIBiG and antiSMASH databases were retrieved from NCBI. These were included in BiG‐SCAPE v2.0.0 analysis using a similarity cut‐off of 0.4 (Zdouc et al. 2025; Draisma et al. 2026). The resulting networks were visualized in Cytoscape v3.10.3 (Shannon et al. 2003). Selected BGCs were compared using Clinker, applying a minimum sequence alignment identity threshold of 40% (Gilchrist and Chooi 2021).

### 5.5 Cultivation of *Actinokineospora* sp. UV203 for Production of Specialized Metabolites and Small‐Scale Extraction

A seed culture of *Actinokineospora* sp. UV203 was prepared by inoculating 25 mL of tryptone soy broth (TSB; Oxoid, USA) with 100 μL of heat‐activated spores from a glycerol stock (40°C for 10 min). The culture was incubated at 21°C with shaking at 200 rpm for 2 days. Subsequently, 5% (v/v) of the seed culture was used to inoculate 50 mL of liquid bioproduction media in 250 mL non‐baffled Erlenmeyer flasks. The tested media included: TSB, ISP2 (Shirling and Gottlieb 1966), SM17 (Zettler et al. 2014), 5254 (g/L: glucose 15.0, soy meal 15.0, corn steep liquor 5.0, CaCO_3_ 2.0, NaCl 5.0; pH 7.0), 5304 (g/L: glucose 1.0, soluble starch 24.0, tryptone 5.0, meat extract 3.0 and CaCO_3_ 4.0, NaCl 5.0; pH 7.0) and 5288 (g/L: glycerol 15.0, soy meal 10.0, NaCl 5.0, CaCO_3_ 1.0, CoCl_2_x7H_2_O 0.001; pH 6.8). Two parallel cultures were prepared for each medium and incubated at 21°C and 200 rpm for 5 and 10 days, respectively. Following incubation, the fermented broths were harvested, frozen at −80°C and freeze‐dried for 72 h until completely dry. The dried material was extracted with methanol (1:1, v/v) by shaking at room temperature for 2 h at 150 rpm. The extracts were centrifuged at 10,000 rpm for 10 min to remove undissolved particles. The resulting organic supernatant was collected and evaporated to dryness using a rotary evaporator at 40°C and 280 mbar, yielding concentrated crude extracts. These were dissolved in 2 mL of methanol, and 250 μL of each extract was filtered through 0.45 μm Whatman Mini‐UniPrep G2 syringeless filters (Cytiva, USA) prior to mass spectrometry analysis. Remaining extracts were stored at −20°C until used for bioactivity tests.

### 5.6 Mass Spectrometry

LC–MS analyses were performed on a Vanquish Horizon UHPLC system (Thermo Fisher Scientific) equipped with an Acquity Premier HSS T3 column, 2.1 × 150 mm, 1.8 μm (Waters) coupled to the ESI source of a timsTOF fleX mass spectrometer (Bruker Daltonics) as described previously (Vignolle et al. 2025). Compass DataAnalysis 5.3 (Bruker Daltonics), GNPS, The Natural Products Atlas, and CAS SciFinder (American Chemical Society) were used for data analysis (Wang et al. 2016; van Santen et al. 2022).

^1^H and ^13^C (DEPTq) 1D as well as COSY, HSQC and HMBC 2D NMR spectra of the isolated compounds **1–4** in CD_3_OD at 298 K were recorded on an Avance NEO 600 NMR spectrometer (Bruker BioSpin) equipped with a N_2_ cryo probe Prodigy BBFO with *z*‐gradient (600.18 MHz for ^1^H, 150.92 MHz for ^13^C). Chemical shifts were calibrated using the ^1^H residual solvent signal at δ = 3.31 and the ^13^C solvent signal at δ = 49.15.

### 5.7 Isolation and Structural Elucidation of Kineochelins

The upscaled culture broth of *Actinokineospora* sp. UV203, harvested on Day 10 of cultivation at 21°C and 200 rpm, was used for the isolation of kineochelins. The total volume of 300 mL was obtained by pooling six parallel 50 mL subcultures grown under identical conditions. The combined culture broth was centrifuged at 10,000 rpm for 10 min, and the resulting supernatant was mixed with 5% (v/v) of methanol‐activated and sterilized Diaion HP20 resin (Supelco, Sigma‐Aldrich, USA) (Bogdanov et al. 2024). The mixture was shaken at 21°C and 200 rpm for 2 h to facilitate adsorption of SMs. After incubation, the aqueous phase was discarded and the resin was extracted with *n*‐butanol under the same shaking conditions (200 rpm, 2 h). The butanol extract was then transferred to a clean glass flask and evaporated to dryness using a rotary evaporator at 40°C. The resulting crude extract was dissolved in 3 mL of methanol and fractionated using FC, which was performed on a PuriFlash 4250 from Interchim equipped with both a photodiode array detector (PDA) and an evaporative light scattering detector (ELSD). The run was performed in reversed phase mode using a PuriFlash 15 C18 HQ 35G column (35.0 g, 22 bar). The mobile phase consisted of water +0.1% formic acid (FA) (A) and acetonitrile: water (9:1) +0.1% FA (B). The following gradient was applied: 5%–20% B in 10 min, 20%–75% B in 60 min, 75%–98% B in 5 min and 98% B for 10 min. The flow rate was set to 15 mL/min. The collected tubes were analysed by UHPLC using a Waters Acquity UPLC H‐Class system (Waters, USA), equipped with a sample manager, a quaternary solvent manager and a column manager. For detection the system was coupled with an ELSD and a PDA detector. The UPLC H‐class system was controlled using Empower 3 software. For analysis, an Acquity TSS H3 (2.1 × 100 mm; 1.8 μm) (Waters, USA) was used. The chromatographic conditions were set as follows: flow rate 0.3 mL/min, water +0.1% FA (A) and acetonitrile: water (9:1) + 0.1% FA (B), gradient: 5%–20% B in 10 min, 20%–75% B in 15 min and 75% B isocratic for 3 min. The tubes were pooled according to UHPLC‐PDA‐ELSD traces into 21 fractions. Following LC–MS analysis, fractions 11 (5.83 mg) and 17 (1.96 mg), containing mainly kineochelin A_1_ and E_1_, respectively, were subjected to NMR analysis.

Marfey's derivatization was employed to determine the absolute configurations of amino acids in kineochelin E_1_ (fraction 16) and A_1_ (fraction 10). Samples of kineochelin E_1_ (410 μg) and A_1_ (750 μg) were dissolved in 2 M HCl, subjected to acid hydrolysis, dried and redissolved in 1 M NaHCO, followed by derivatization with 1% Marfey's reagent (1‐fluoro‐2,4‐dinitrophenyl‐5‐L‐alanineamide (L‐FDAA)) in acetone. Subsequently, the mixtures were heated at 40°C for 1 h. Thereafter, neutralization with 1 M HCl was carried out, followed by dilution with acetonitrile for LC–MS analysis. For comparison, standard amino acids, L/D‐Orn, L/D‐Thr, L/D‐allo‐Thr and L/D‐Ser, were similarly derivatized with L‐FDAA.

### 5.8 Growth and Kineochelin Production Dynamics of *Actinokineospora* sp. UV203

To assess the growth kinetics and dynamics of kineochelin production under varying iron conditions, we used a 72‐h seed culture and subsequent culture prepared as described above. Cultivation was carried out in 50 mL volumes of the following media: SM17 (control), SM17 supplemented with 100 and 200 μM FeCl_3_ (iron‐rich condition) and SM17 supplemented with 100 μM 2,2′‐bipyridine (iron‐limited condition). Both supplements were filter‐sterilized and added to the media prior to inoculation. Each condition was tested in three biological replicates. Cultures were sampled at 12‐h intervals for two purposes: (i) colony‐forming unit (CFU) determination using 100 μL aliquots and (ii) analysis of kineochelin production and total siderophore activity using 2 mL samples. CFU determination was performed using the plate count method, with serial dilutions in 0.9% NaCl and plating onto SFM agar (van Dissel and van Wezel 2018). CFU values were log₁₀‐transformed prior to visualization. Growth curves were plotted from the mean of three biological replicates, with error bars representing standard deviation (SD). The area under each growth curve (AUC) was calculated using the linear trapezoidal rule (Worth and Espina 2022). Differences in AUC among conditions were tested by one‐way ANOVA, followed by Tukey's HSD post‐hoc test (Agbangba et al. 2024).

To quantify iron‐chelating activity during growth, we used a universal CAS assay in 96‐well microplate format (Schwyn and Neilands 1987; Arora and Verma 2017). Briefly, 100 μL of culture supernatant or extract solution was mixed with an equal volume of CAS reagent containing Fe^3+^, incubated in the dark for 20 min and absorbance was measured at 630 nm (Arora and Verma 2017). Siderophore production was quantified as percent siderophore units (PSU), calculated using the formula published by Payne (1993). All measurements were performed in three independent biological replicates. CAS absorbance values were background‐corrected using CAS reagent mixed with uninoculated medium containing the corresponding supplements. Negative values, which can arise from iron carryover in iron‐supplemented cultures, were interpreted as baseline (no detectable siderophore activity) and truncated to zero for visualization.

To assess metal‐binding specificity beyond ferric iron, a modified CAS assay was performed using CAS complexes prepared with alternative metal ions. CAS reagents were freshly prepared using chloride salts of Fe^2+^, Mg^2+^, Mn^2+^, V^3+^, Zn^2+^, Se^4+^, Co^2+^, Cu^2+^, Ni^2+^ and gallium bromide (Ga^3+^), following published protocols (Kuzyk et al. 2021; Patel et al. 2018). To determine whether kineochelins alone are sufficient to account for the CAS activity observed in culture supernatants, a pre‐purified kineochelin‐enriched fraction was analysed. All measurements were performed using technical replicates (*n* = 3). Because culture media and extract solutions exhibited intrinsic absorbance at 630 nm, all CAS measurements were corrected for sample‐specific background signals. Metal‐binding activity was quantified as absolute CAS decolorization (ΔA), calculated as the difference in absorbance between the corresponding metal–CAS control and sample after subtraction of relevant background signals.

### 5.9 Bioactivity Assays

Antimicrobial bioactivity was assessed using the disc diffusion method with 6‐mm Whatman paper discs. Discs were impregnated with either 50 μL of crude extract for bacterial assays or 100 μL for yeast assays. For tests involving pre‐purified kineochelins, extracts were first dried, weighed and dissolved to a known concentration (mg/mL). Inhibition zones were recorded after 18–48 h of incubation at appropriate temperatures. Mueller–Hinton agar (MHA) was used for clinically relevant bacterial strains, while yeasts were tested on MHA supplemented with 2% glucose and 0.5 μg/mL methylene blue dye, as recommended by The Clinical & Laboratory Standards Institute (CLSI) guidelines (CLSI 2015; Procop 2022). For environmental isolates, media were selected based on organism‐specific growth requirements; MHA was used where appropriate (Table S1). The sensitivity profiles of the clinical yeast isolates used in this study were determined by the University Hospital in Brno, as listed in Table S11.

To determine the minimum inhibitory concentrations (MICs) against sensitive yeast strains, we followed the 96‐well microtiter plate protocol according to CLSI guidelines (CLSI 2015; Berkow et al. 2020). RPMI 1640 medium, supplemented with glucose and buffered with MOPS to pH 7.0, was used for testing *N. glabratus* strains. For *S. cerevisiae* , which failed to grow in both RPMI and MHA with glucose, YPD medium (DSMZ medium 393) was used instead. Kineochelin‐enriched pre‐purified fractions containing multiple congeners were tested starting from a concentration of 20 mg/mL using two‐fold serial dilutions. Amphotericin B served as a positive control, starting at 125 μg/mL.

Human cancer cell lines for the initial screening of potential antiproliferative activity of kineochelins were purchased from ATCC (American Type Culture Collection; Manassas, VA, USA) or ECACC (European Collection of Authenticated Cell Cultures, Salisbury, United Kingdom). The Jurkat (human leukaemic T cell lymphoma) and HCT116 (human colorectal carcinoma) were cultured in RPMI 1640 medium (Biosera, Kansas City, MO, United States), while U‐87 MG (human glioblastoma), A2058 (human metastatic melanoma) and MDA‐MB‐231 (human mammary gland adenocarcinoma) cells were maintained in growth medium consisting of high‐glucose Dulbecco's Modified Eagle Medium (DMEM) supplemented with sodium pyruvate (Biosera, Kansas City, MO, United States). Specific medium requirements were necessary for PaTu 8902 (human pancreatic adenocarcinoma) cells, which were maintained in high‐glucose DMEM supplemented with sodium pyruvate (Biosera, Kansas City, MO, United States) and 25 mM HEPES (Sigma, Steinheim, Germany). The healthy, non‐tumorigenic cell line CCD‐18Co (non‐malignant intestinal fibroblasts) was cultured in DMEM (Biosera, Kansas City, MO, USA). All culture media were supplemented with 10% FBS or 15% in the case of the A2058 cell line (fetal bovine serum; Gibco, Thermo Scientific, Rockford, IL, USA) and an antibiotic–antimycotic solution (Merck, Darmstadt, Germany). Throughout the experiment, cell lines were cultured at 37°C in a humidified atmosphere containing 5% CO_2_. The effects of a pre‐purified kineochelin‐enriched extract, tested as two independently handled aliquots (designated E1 and E2), were determined using a resazurin reduction‐based assay. A549, U‐87 MG, PaTu 8902, Jurkat, HCT116, A2058, MDA‐MB‐231 and CCD‐18Co (5 × 10^3^/well) cell lines were seeded and cultured in 96‐well culture plates. After 24 h, cells were treated with E1, E2 pre‐purified kineochelin extracts (concentration range 10–750 μg/mL) and incubated for 72 h. After 72 h incubation, 10 μL of resazurin dye was added to each well, followed by incubation for a minimum of 1.5 h. The fluorescent output was measured using the automated Cytation 3 Cell Imaging Multi‐Mode Reader (Biotek, Winooski, VT, USA).

### 5.10 Knock‐Out Mutagenesis

All standard molecular biology techniques, including DNA manipulations, cloning procedures and plasmid transformation into *Escherichia coli* strains, were performed as previously described (Sambrook et al. 1989). PCR amplifications were carried out using Q5 High‐Fidelity DNA Polymerase (New England Biolabs, Ipswich, MA, USA) with oligonucleotides listed in Table S12. A complete list of plasmids and bacterial strains used or constructed in this study is provided in Table S13. Luria‐Bertani (LB) medium was used for routine cultivation of *E. coli* , supplemented with chloramphenicol (30 μg/mL), kanamycin (30 μg/mL), and when necessary, apramycin (Am, 100 μg/mL). *E.coli* DH5α was employed for general cloning, while *E. coli* ET12567 (pUZ8002) was used to mediate intergenic conjugative plasmid transfer into *Actinokineospora* sp. UV203. To assess the roles of candidate biosynthetic genes in kineochelin production, two *kin* cluster genes, kin_2475 (*kinB*) and kin_2479 (*kinA*), were selected for inactivation. Each gene was PCR‐amplified using primers incorporating EcoRI and HindIII restriction sites, and cloned into the 3.1 kb EcoRI/HindIII fragment of the pSOK201 vector containing ColE1, *oriT* and Am^R^ (Zotchev et al. 2000). The resulting plasmids, pKO_2475 and pKO_2479, were confirmed by sequencing and subsequently conjugated into the wild‐type *Actinokineospora* sp. UV203 strain. Apramycin (50 μg/mL) was used for selection of recombinant *Actinokineospora* strains and nalidixic acid (30 μg/mL) as counterselection against *E. coli* . No exconjugants were recovered for either construct under the conditions tested. As a conjugation control, an empty pSET152 vector (Flett et al. 1997) was introduced into the wild‐type strain using the same conjugation procedure.

### 5.11 Transcriptomics

RNA extraction and sequencing was performed by the Joint Microbiome Facility of the Medical University of Vienna and the University of Vienna (project JMF‐2412‐11). RNA was extracted using the Monarch Total RNA Miniprep Kit (New England Biolabs) according to the manufacturer's instructions but including two rounds of DNA digestion with ezDNAse (Thermo Fischer Scientific). Sequencing libraries were prepared from rRNA depleted (Ribo‐Zero Plus rRNA Depletion Kit, Illumina) RNA samples using the NEBNext Ultra II Directional RNA Library Prep Kit for Illumina, New England Biolabs according to the manufacturer's instructions and sequenced in 2× 100 bp paired‐end mode (Illumina NextSeq 6000 SP 1/2 flowcell), yielding 76 million raw reads per sample. Individual read libraries were quality checked using fastQC v0.12.1 (Andrews 2010) and quality statistics were merged using multiQC v1.21 (Ewels et al. 2016). Original bam files were converted to fastq format using samtools (Li et al. 2009) v1.12's bam2fq function. Fastq files were trimmed and filtered using BBDuk, in BBMap v39.10. PhiX and adapter sequences were removed and the chastityfilter option was set to ‘true’. Reads were also trimmed at a q‐score of 28, with an average q‐score of 15, keeping a minimum length of 30 nucleotides and the following other options: ‘ktrim=*r* k=23 mink=11 hammingdistance=1 qtrim=*r*’. Reads were mapped to the reference genome UV203 using BBMap with a minimum percent identity of 98% and ambiguous reads were mapped to all locations. The resulting sam file was converted to a bam file using samtools. FeatureCounts, part of SubRead v2.1.1, was used to generate a counts table for reversely‐stranded, paired‐end reads with a PGAP‐generated .gff file as a reference. DESeq2 release v1.48 (Love et al. 2014) was used to assess differentially‐expressed genes and calculated FPKMs for each. Normalized counts from DESeq2 were used to generate principal component analysis (PCA) plots and volcano plots (log_2_ fold change, log_2_FC, vs. −log_10_ false discovery rate, FDR), applying significance thresholds of |log_2_FC| ≥ 1 and FDR < 0.05. antiSMASH BGC predictions were overlaid on PGAP annotations based on genome coordinates using in‐house parsing script (https://github.com/kralovaresearch/Kineochelin).

## Author Contributions

**Johannes Gafriller:** investigation, writing – review and editing. **Peter Spacek:** investigation, writing – review and editing. **Matej Bezdicek:** investigation, funding acquisition, writing – review and editing. **Joana Séneca:** writing – review and editing, software, formal analysis, data curation. **Thomas Rattei:** funding acquisition, writing – review and editing, validation. **Alexander Loy:** conceptualization, funding acquisition, writing – original draft, writing – review and editing, supervision, resources, project administration, methodology. **Olga N. Sekurova:** supervision, writing – review and editing. **Viktoria Medvedcova:** investigation, writing – review and editing. **Martin Zehl:** funding acquisition, writing – review and editing, conceptualization, methodology, visualization, software, supervision, resources, formal analysis, data curation, writing – original draft, investigation, validation. **Ulrike Grienke:** investigation, writing – original draft, writing – review and editing, methodology, formal analysis, validation. **Stanislava Kralova:** conceptualization, investigation, funding acquisition, writing – original draft, methodology, visualization, writing – review and editing, formal analysis, data curation, validation, software, resources, supervision, project administration. **Sergey B. Zotchev:** conceptualization, supervision, resources, writing – review and editing, writing – original draft, funding acquisition, methodology. **Jay Osvatic:** formal analysis, data curation, software, writing – review and editing.

## Funding

This work was supported by Horizon 2020 Framework Programme, 101020356; Universität Wien, MetaBac; Ministry of Education, Youth and Sports, VAN 2025; Masarykova Univerzita, MUNI/SC/1946/2024; Austrian Science Fund, 10.55776/COE7; Ministerstvo Zdravotnictví České Republiky, FNBr, 65269705; Vedecká grantová agentúra Ministerstva školstva, výskumu, vývoja a mládeže Slovenskej republiky a Slovenskej akadémie vied, VEGA 1/0498/23.

## Ethics Statement

The authors have nothing to report.

## Consent

The authors have nothing to report.

## Conflicts of Interest

The authors declare no conflicts of interest.

## Supporting information


**Figure S1:** 16S rRNA gene phylogeny showing the placement of *Actinokineospora* sp. UV203 within the genus; scale bar indicates 0.01 substitutions per site. Numbers at nodes denote the bootstrap values (> 50%) for branch points based on 1000 replications.
**Figure S2:** Base peak chromatograms showing retention time regions with differential metabolite production between UV203 mutants. Inhibition assays on medium with *Micrococcus luteus* as the indicator strain. Zones: (1) negative control; (2) UV203 wild‐type; (3) UV203_T3_9 mutant (ermE*p with LuxR promoter); (4) UV203_TC10 mutant (empty vector). Asterisks indicate kineochelin congeners.
**Figure S3:** Purity of isolated kineochelin E_1_. UHPLC‐ELSD chromatogram (A) and UHPLC–MS base peak chromatogram (B) of fraction 17 (2.0 mg) containing kineochelin E_1_ (**1**) as main compound.
**Figure S4:** ^1^H NMR spectrum of kineochelin E_1_ (**1**) in CD_3_OH at 600 MHz.
**Figure S5:** ^13^C (DEPTq) NMR spectrum of kineochelin E_1_ (**1**) in CD_3_OH at 151 MHz.
**Figure S6:** COSY spectrum of kineochelin E_1_ (**1**) in CD_3_OH at 600 MHz.
**Figure S7:** HSQC spectrum of kineochelin E_1_ (**1**) in CD_3_OH at 600 MHz.
**Figure S8:** HMBC spectrum of kineochelin E_1_ (**1**) in CD_3_OH at 600 MHz.
**Figure S9:** Marfey's analysis of kineochelin E_1_ and kineochelin A_1_. Extracted ion chromatograms (*m/z* 372.1150 ± 0.0050) showing the signals for L‐FDAA‐derivatised free amino acids L‐Thr, D‐Thr, D‐*allo*‐Thr and L‐*allo*‐Thr, as well as of L‐Thr in the hydrolysed fraction F16, containing mainly kineochelin E_1_ and hydrolysed fraction F10, containing mainly kineochelin A_1_. The bottom two EICs show the standard addition experiments confirming presence of pure L‐Thr in kineochelin E_1_ and kineochelin A_1_.
**Figure S10:** Purity of isolated kineochelin A_1_. UHPLC‐ELSD chromatogram of fraction 11 (5.8 mg) containing kineochelin A_1_ (**2**) as main compound.
**Figure S11:** ^1^H NMR spectrum of kineochelin A_1_ (**2**) in CD_3_OH at 600 MHz.
**Figure S12:** ^13^C (DEPTq) NMR spectrum of kineochelin A_1_ (**2**) in CD_3_OH at 151 MHz.
**Figure S13:** COSY spectrum of kineochelin A_1_ (**2**) in CD_3_OH at 600 MHz.
**Figure S14:** HSQC spectrum of kineochelin A_1_ (**2**) in CD_3_OH at 600 MHz.
**Figure S15:** HMBC spectrum of kineochelin A_1_ (**2**) in CD_3_OH at 600 MHz.
**Figure S16:** High resolution ESI‐Qq‐TOF mass spectrum of kineochelin A_1_ (A) and high resolution MS/MS spectrum of its [M + H]^+^ ion (B).
**Figure S17:** High resolution ESI‐Qq‐TOF mass spectrum of kineochelin A_2_ (A) and high resolution MS/MS spectrum of its [M + H]^+^ ion (B).
**Figure S18:** High resolution ESI‐Qq‐TOF mass spectrum of kineochelin B_1_ (A) and high resolution MS/MS spectrum of its [M + H]^+^ ion (B).
**Figure S19:** High resolution ESI‐Qq‐TOF mass spectrum of kineochelin B_2_ (A) and high resolution MS/MS spectrum of its [M + H]^+^ ion (B).
**Figure S20:** High resolution ESI‐Qq‐TOF mass spectrum of kineochelin C_1_ (A) and high resolution MS/MS spectrum of its [M + H]^+^ ion (B).
**Figure S21:** High resolution ESI‐Qq‐TOF mass spectrum of kineochelin D_1_ (A) and high resolution MS/MS spectrum of its [M + H]^+^ ion (B).
**Figure S22:** High resolution ESI‐Qq‐TOF mass spectrum of kineochelin E_1_ (A) and high resolution MS/MS spectrum of its [M + H]^+^ ion (B).
**Figure S23:** High resolution ESI‐Qq‐TOF mass spectrum of kineochelin E_2_ (A) and high resolution MS/MS spectrum of its [M + H]^+^ ion (B).
**Figure S24:** High resolution ESI‐Qq‐TOF mass spectrum of vulnibactin 2 (A) and high resolution MS/MS spectrum of its [M + H]^+^ ion (B).
**Figure S25:** High resolution ESI‐Qq‐TOF mass spectrum of pseudomobactin A (A) and high resolution MS/MS spectrum of its [M + H]^+^ ion (B).
**Figure S26:** High resolution ESI‐Qq‐TOF mass spectrum of asteroidic acid (A) and high resolution MS/MS spectrum of its [M + H]^+^ ion (B).
**Figure S27:** Marfey's analysis of kineochelin A_1_. Extracted ion chromatograms (*m/z* 358.0993 ± 0.0050, red; *m/z* 385.1466 ± 0.0050, purple; *m/z* 637.1961 ± 0.0050, blue) showing the signals for L‐FDAA‐derivatised free amino acids L‐Ser, D‐Ser, L‐Orn and D‐Orn, as well as of D‐Ser and L‐Orn in the hydrolysed fraction F10, containing mainly kineochelin A_1_.
**Figure S28:** Planar structures of cyclo(Hyp‐Leu) (**3**) and cyclo(Hyp‐Phe) (**4**) with atom numbering and ^1^H‐^1^H COSY and key ^1^H‐^13^C HMBC correlations.
**Figure S29:** ^1^H NMR spectrum of cyclo(Hyp‐Leu) (**3**) in CD_3_OH at 600 MHz.
**Figure S30:** COSY spectrum of cyclo(Hyp‐Leu) (**3**) in CD_3_OH at 600 MHz.
**Figure S31:** HSQC spectrum of cyclo(Hyp‐Leu) (**3**) in CD_3_OH at 600 MHz.
**Figure S32:** HMBC spectrum of cyclo(Hyp‐Leu) (**3**) in CD_3_OH at 600 MHz.
**Figure S33:** ^1^H NMR spectrum of cyclo(Hyp‐Phe) (**4**) in CD_3_OH at 600 MHz.
**Figure S34:** COSY spectrum of cyclo(Hyp‐Phe) (**4**) in CD_3_OH at 600 MHz.
**Figure S35:** HSQC spectrum of cyclo(Hyp‐Phe) (**4**) in CD_3_OH at 600 MHz.
**Figure S36:** HMBC spectrum of cyclo(Hyp‐Phe) (**4**) in CD_3_OH at 600 MHz.
**Figure S37:** Principal component analysis (PCA) of RNA‐seq samples. PCA of normalized transcript counts from four conditions (SM17 vs. SM17 + 200 μM FeCl₃ at Days 3 and 7). Samples separate cleanly (PC1 = 34.9%, PC2 = 21.3%); PC1 distinguishes medium and PC2 captures the time shift. Biological replicates cluster tightly within each group.
**Figure S38:** Comparative transcriptomics of *Actinokineospora* sp. UV203 under siderophore‐producing and siderophore‐depleted growth conditions. Volcano plots for the indicated pairwise contrasts. Each point is a gene (*x*‐axis: log_2_ fold change; *y*‐axis: −log_10_ [FDR]); dashed lines mark significance thresholds (|log_2_FC| ≥ 1 and FDR < 0.05). PCA and volcano plots were generated based on normalized counts from DESeq.
**Figure S39:** LC–MS base peak chromatogram of the pre‐purified butanol‐phase extract showing the kineochelin mixture and co‐purified known shunt products.
**Figure S40:** Metal‐binding activity of UV203 SM17 culture supernatant measured by Chrome Azurol S (CAS)‐shuttle assay. Absolute CAS decolorization (ΔA) values are shown for individual metal–CAS complexes. Points represent technical replicates (*n* = 3), and colours indicate relative binding strength normalized to the Fe^3+^ response.
**Table S1:**. Results from the untargeted LC–MS‐based secondary metabolomics analysis of *Actinokineospora* sp. UV203 grown in different media. Groups of secondary metabolites known or presumed to be biosynthetically related are highlighted by the same colour, whereby usually only the most abundant congeners are reported. The listed isoflavone derivatives were only found in cultures from soy‐containing media, but not in the media controls. They are thus assumed to be biotransformation products from the soy‐isoflavones present in these media.
**Table S2:** ^1^H (600 MHz) and ^13^C NMR data (151 MHz) of kineochelin E_1_ (**1**) in CD_3_OD in comparison with literature data for pseudomobactin A (*δ* in ppm). A second set of signals (**1′**) was observed in a ratio of approximately 3:5 to the one shown in Table 2. This set of signals was initially hypothesized to belong to a stereoisomer of **1**, but after Marfey's analysis is now assumed to belong to a stable conformer or metal ion complex of kineochelin E_1_.
**Table S3:** ^1^H (600 MHz, CD_3_OD) data of cyclo(Hyp‐Leu) (**3**) and cyclo(Hyp‐Phe) (**4**) in comparison with literature data (*δ* in ppm).
**Table S4:** Local gene identities between *kin* cluster and related amychelin, cahuitamycin and gobichelin clusters.
**Table S5:** Closest NCBI Blast and antiSMASH database matches to the core biosynthetic enzymes KinA, KinB and KinC in the *kin* cluster.
**Table S6:** Closest homologues of coding sequences (CDSs) within the *kin* biosynthetic gene cluster of *Actinokineospora* sp. UV203.
**Table S7:** Microbial strains used for preliminary antimicrobial susceptibility testing of the kineochelins.
**Table S8:**. Minimum inhibitory concentration (MIC) and Minimum fungicidal concentration (MFC) values recorded for tested yeasts.
**Table S9:**. Results of screening antiproliferative assays with pre‐purified kineochelin‐enriched fraction.
**Table S10:** Information on genome assembly from *Actinokineospora* sp. UV203.
**Table S11:** Clinical yeast strains used in this study and their antifungal susceptibility determined by minimum inhibitory concentrations according to EUCAST breakpoints.
**Table S12:** List of all primers used in this study.
**Table S13:** List of all strains and vectors used in cloning part of this study.

## Data Availability

The genome sequence and transcriptomic data of strain *Actinokineospora* sp. UV203 are available on NCBI (BioProject accession number PRJNA1331526). The nearly full‐length 16S rRNA gene (1395 bp) of strain *Actinokineospora* sp. UV203 is available on NCBI (accession number PX090945). The NMR data of kineochelin E_1_ and A_1_ are deposited in the Natural Products Magnetic Resonance Database (NP‐MRD) under accession numbers NP0352113 and NP0352114, respectively.
