# Supplementary material for: Kineochelins—A New Group of Siderophores From an Antarctic Bacterium
Source: Microb Biotechnol. 2026 May 28;19(6):e70386. doi: 10.1111/1751-7915.70386 (PMC13238742; doi:10.1111/1751-7915.70386)
Supplement: Supplementary file 1 — Figure S1: 16S rRNA gene phylogeny showing the placement of Actinokineospora sp. UV203 within the genus; scale bar indicates 0.01 substitutions per site. Numbers at nodes denote the bootstrap values (> 50%) for branch points based on 1000 replications. Figure S2: Base peak chromatograms showing retention time regions with differential metabolite production between UV203 mutants. Inhibition assays on medium with Micrococcus luteus as the indicator strain. Zones: (1) negative control; (2) UV203 wild‐type; (3) UV203_T3_9 mutant (ermE*p with LuxR promoter); (4) UV203_TC10 mutant (empty vector). Asterisks indicate kineochelin congeners. Figure S3: Purity of isolated kineochelin E1. UHPLC‐ELSD chromatogram (A) and UHPLC–MS base peak chromatogram (B) of fraction 17 (2.0 mg) containing kineochelin E1 (1) as main compound. Figure S4: 1H NMR spectrum of kineochelin E1 (1) in CD3OH at 600 MHz. Figure S5: 13C (DEPTq) NMR spectrum of kineochelin E1 (1) in CD3OH at 151 MHz. Figure S6: COSY spectrum of kineochelin E1 (1) in CD3OH at 600 MHz. Figure S7: HSQC spectrum of kineochelin E1 (1) in CD3OH at 600 MHz. Figure S8: HMBC spectrum of kineochelin E1 (1) in CD3OH at 600 MHz. Figure S9: Marfey's analysis of kineochelin E1 and kineochelin A1. Extracted ion chromatograms (m/z 372.1150 ± 0.0050) showing the signals for L‐FDAA‐derivatised free amino acids L‐Thr, D‐Thr, D‐allo‐Thr and L‐allo‐Thr, as well as of L‐Thr in the hydrolysed fraction F16, containing mainly kineochelin E1 and hydrolysed fraction F10, containing mainly kineochelin A1. The bottom two EICs show the standard addition experiments confirming presence of pure L‐Thr in kineochelin E1 and kineochelin A1. Figure S10: Purity of isolated kineochelin A1. UHPLC‐ELSD chromatogram of fraction 11 (5.8 mg) containing kineochelin A1 (2) as main compound. Figure S11: 1H NMR spectrum of kineochelin A1 (2) in CD3OH at 600 MHz. Figure S12: 13C (DEPTq) NMR spectrum of kineochelin A1 (2) in CD3OH at 151 MHz. Figure S13: COSY spectrum of [file MBT2-19-e70386-s001.docx]

**Supplementary Material:**

**Kineochelins - a new group of siderophores from an Antarctic bacterium**

Stanislava Kralova🖂^1,2^, Peter Spacek^1,2^, Johannes Gafriller ^3,4^, Matej Bezdicek^5,6^, Viktoria Medvedcova^7^, Joana Séneca^1,8^, Jay Osvatic^9^, Ulrike Grienke^3^, Thomas Rattei^10^, Olga N. Sekurova^3^, Sergey B. Zotchev^3^*, Martin Zehl🖂^11,12^*, and Alexander Loy^1,8,13^*

^1^ Division of Microbial Ecology, Centre for Microbiology and Environmental Systems Science, University of Vienna, Vienna, Austria

^2^ Department of Molecular Pharmacy, Faculty of Pharmacy, Masaryk University, Brno, Czech Republic

^3^ Department of Pharmaceutical Sciences, University of Vienna, Vienna, Austria

^4^ Vienna Doctoral School of Pharmaceutical, Nutritional and Sport Sciences, University of Vienna, Vienna, Austria

^5^ Division of Clinical Microbiology and Immunology, Department of Laboratory Medicine, University Hospital Brno, Brno, Czech Republic

^6^ Division of Clinical Microbiology and Immunology, Department of Laboratory Medicine, Faculty of Medicine, Masaryk university, Brno, Czech Republic

^7^ Department of Pharmacology, Faculty of Medicine, Pavol Jozef Šafárik University, Košice, Slovakia

^8^ Joint Microbiome Facility of the Medical University of Vienna and the University of Vienna, Medical University of Vienna, University of Vienna, Vienna, Austria

^9^ Department of Laboratory Medicine, Medical University of Vienna,
Vienna, Austria

^10^ Division of Computational Systems Biology, Centre for Microbiology and Environmental Systems Science, University of Vienna, Vienna, Austria

^11^ Department of Analytical Chemistry, Faculty of Chemistry, University of Vienna, Vienna, Austria

^12^ Institute of Science and Technology Austria (ISTA), Am Campus 1, Klosterneuburg, Austria

^13^ Austrian Polar Research Institute, Vienna, Austria

*These authors contributed equally

Correspondence:

Stanislava Kralova, [bezdicekkralovas@pharm.muni.cz](mailto:bezdicekkralovas@pharm.muni.cz)

Martin Zehl, [Martin.Zehl@ist.ac.at](mailto:Martin.Zehl@ist.ac.at)

**Supplementary Note**

**Initial prioritization and activation attempts targeting BGC 2.18**

During early metabolomic analyses of *Actinokineospora* sp. UV203, we prioritized a pair of low-abundance isomeric metabolites detected as [M+H]^+^ ions at *m/z* 575.0587±0.0029, corresponding to the proposed sum formula of C_22_H_24_Cl_2_N_4_O_6_S_2_. They stood out by the presence of two chlorine atoms as inferred from the isotopic pattern and two sulphur atoms (Supplementary Table S1). BGC 2.18 represented the most plausible biosynthetic pathways, as it is the only cluster in the UV203 genome encoding a halogenase specifically a tryptophane-halogenase. In addition, it contains at least two non-ribosomal peptide synthetase (NRPS)-modules both predicted to incorporate a cysteine, the sulphur-containing amino acid. Despite partial similarity to the coelibactin BGC detected by antiSMASH, the detected chlorinated metabolites were inconsistent with coelibactin[1], and no other known product could be confidently assigned to BGC 2.18.

Since the wild-type strain produced these metabolites only in trace amounts under all tested cultivation conditions, we attempted targeted overexpression of BGC2.18. We individually cloned the pathway specific LuxR- and HxlR-like regulatory genes encoded within the cluster into pSET152-based integrative expression vectors under the control of either the constitutive *ermE*p* promoter[2]. While particularly the overexpression of LuxR seemed to have a positive effect, the production was still far too low to attempt isolation, likely due to host-specific promoter inefficiency[3,4]. As a second strategy, we replaced the *ermE**p promoter in the vectors with the promoter from the UV203 *rrn* operon, which is considered highly active based on its strong transcriptional output in bacteria[5,6]. However, while again showing positive effects compared to the empty vector control, this modification also failed to sufficiently raise the production of the target metabolites for purification. This indicates that additional regulatory or physiological constraints are in place in the native host. Following these unsuccessful activation attempts, we redirected subsequent genetic engineering efforts toward the *kin* BGC.

**Supplementary Figures and Tables**

**
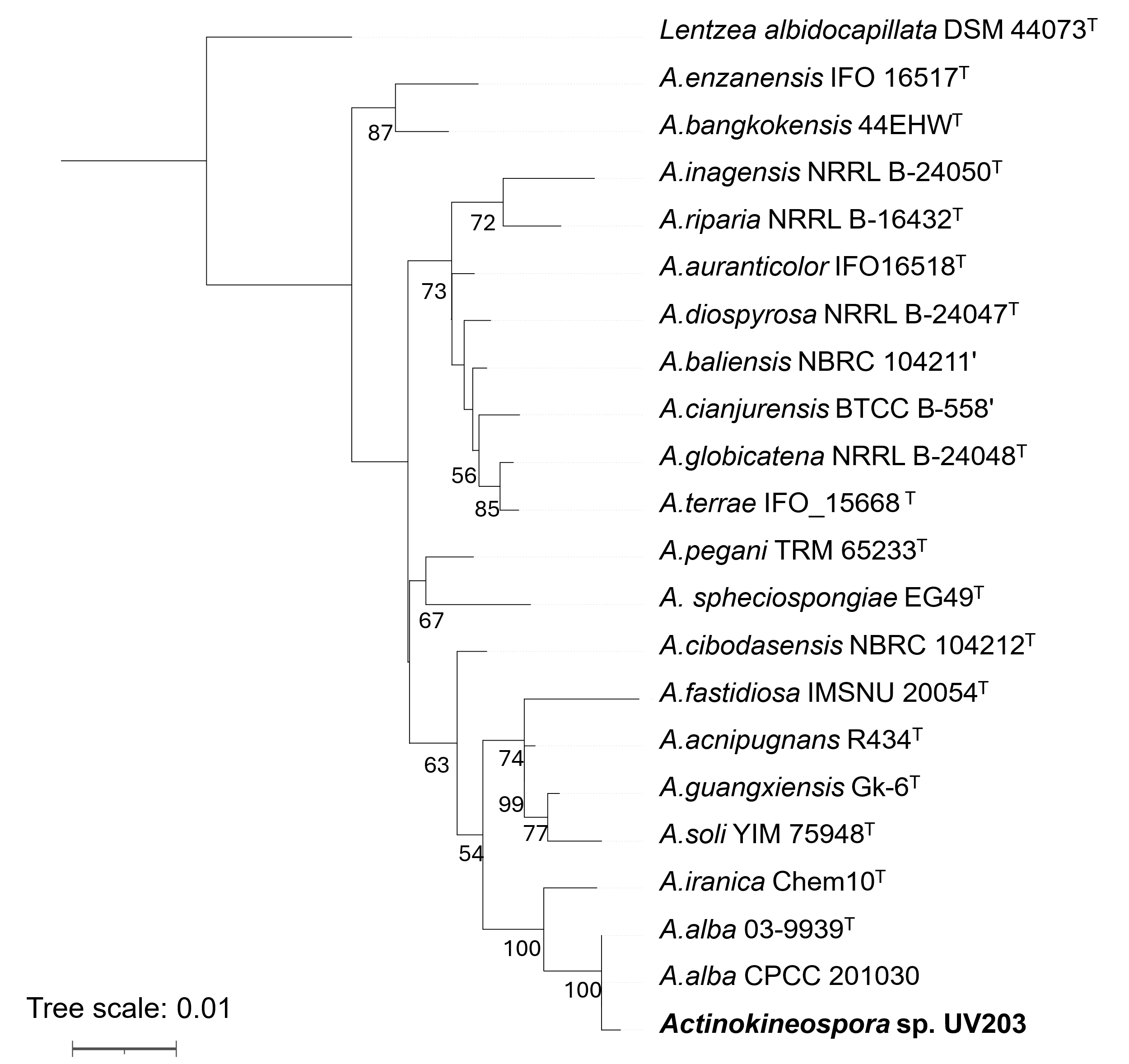
**

**Supplementary Figure S1.** 16S rRNA gene phylogeny showing the placement of *Actinokineospora* sp. UV203 within the genus; scale bar indicates 0.01 substitutions per site. Numbers at nodes denote the bootstrap values (>50%) for branch points based on 1,000 replications.


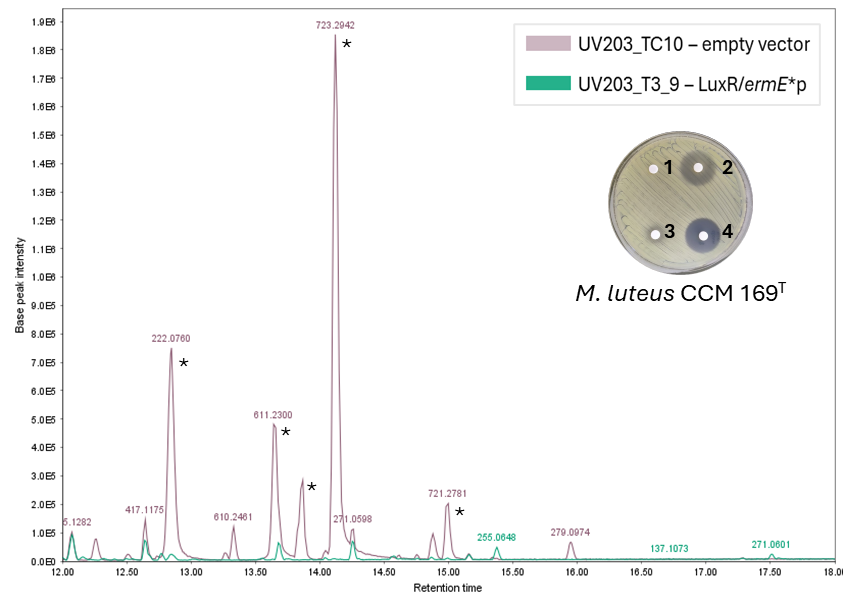


**Supplementary Figure S2.** Base peak chromatograms showing retention time regions with differential metabolite production between UV203 mutants. Inhibition assays on medium with *Micrococcus luteus* as the indicator strain. Zones: (1) negative control; (2) UV203 wild-type; (3) UV203_T3_9 mutant (*ermE**p with LuxR promoter); (4) UV203_TC10 mutant (empty vector). Asterisks indicate kineochelin congeners.

__

**Supplementary Figure S3.** Purity of isolated kineochelin E_1_. UHPLC-ELSD chromatogram (A) and UHPLC-MS base peak chromatogram (B) of fraction 17 (2.0 mg) containing kineochelin E_1_ (**1**) as main compound.


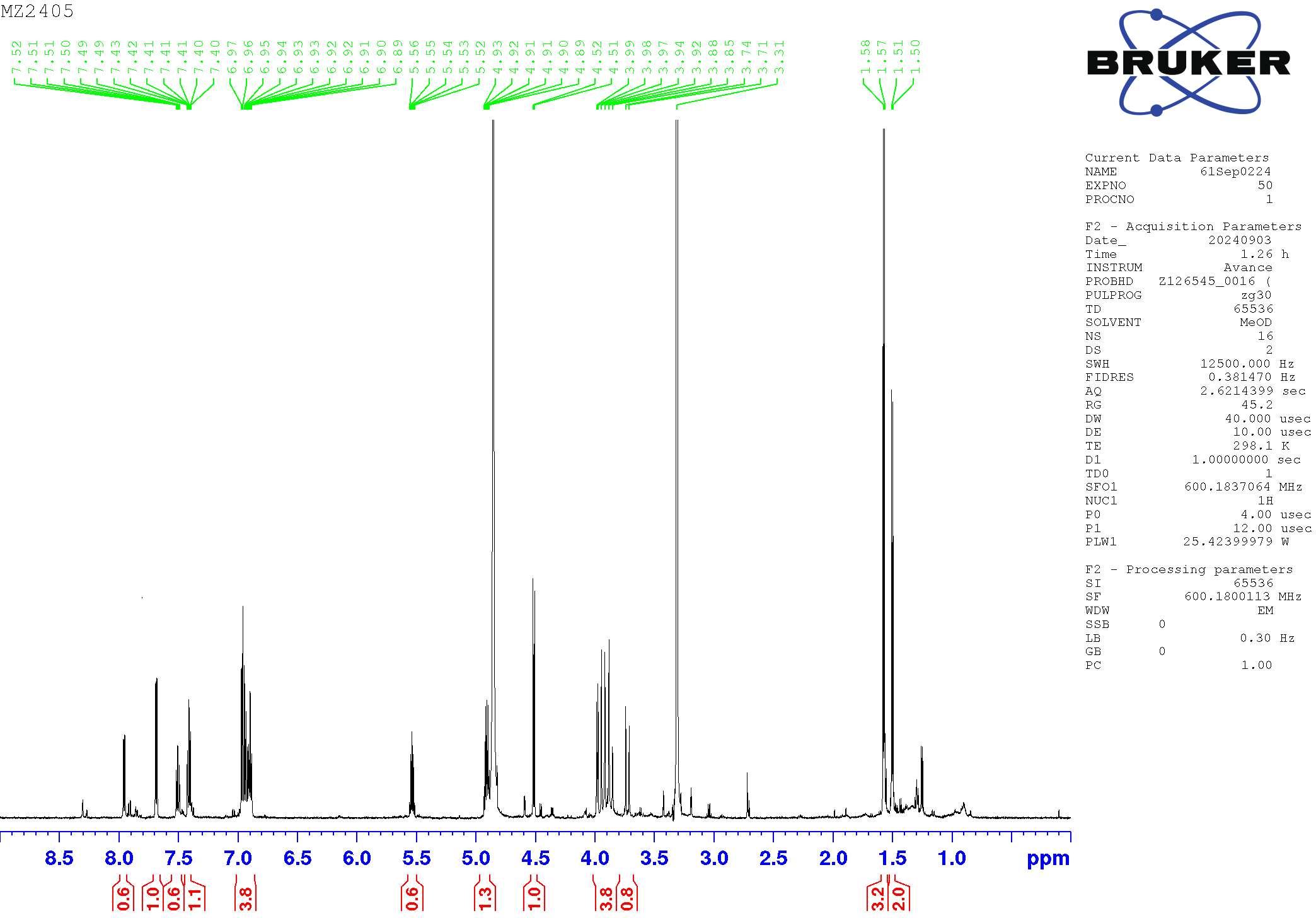


**Supplementary Figure S4.** ^1^H NMR spectrum of kineochelin E_1_ (**1**) in CD_3_OH at 600 MHz.


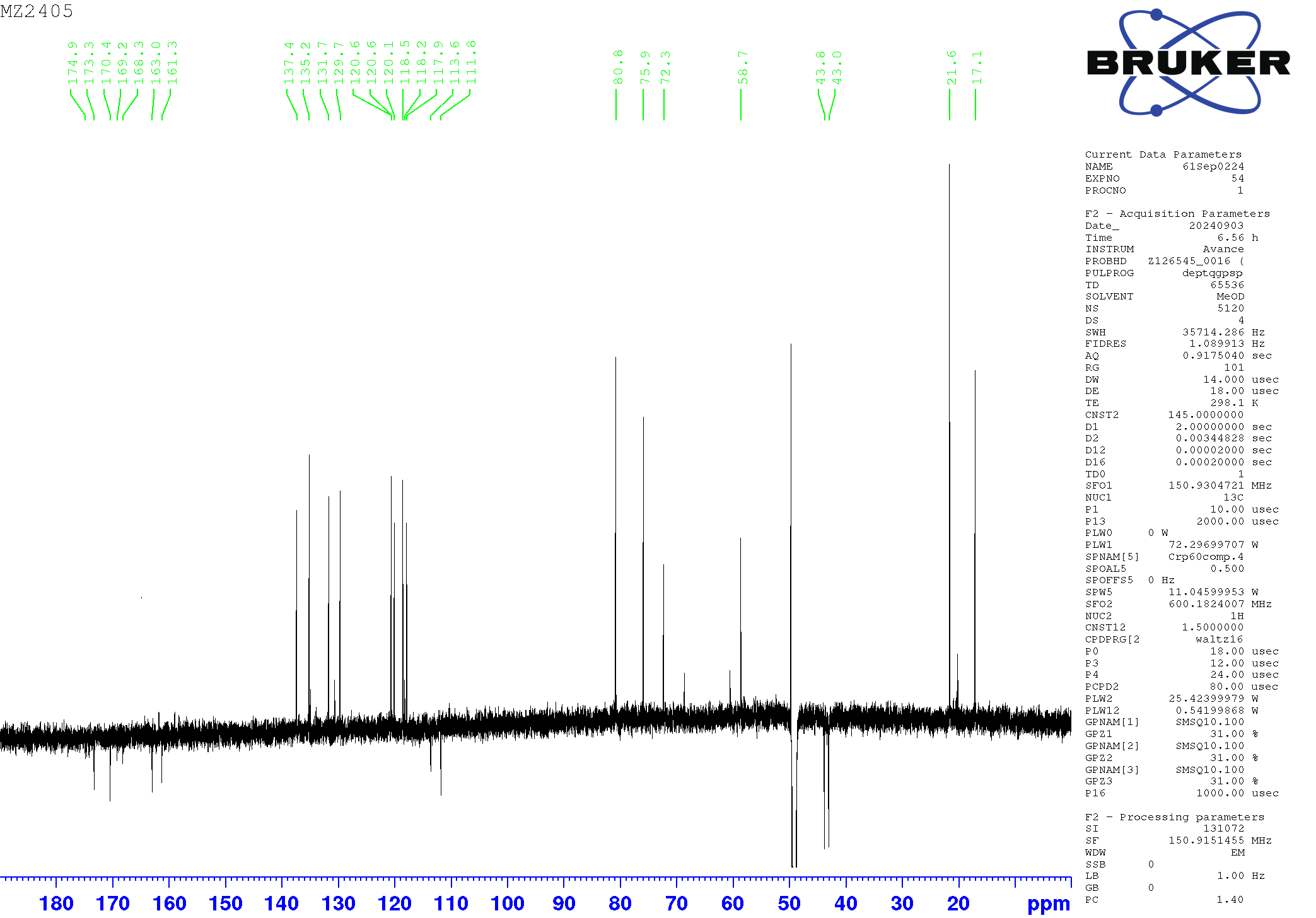


**Supplementary Figure S5.** ^13^C (DEPTq) NMR spectrum of kineochelin E_1_ (**1**) in CD_3_OH at 151 MHz.


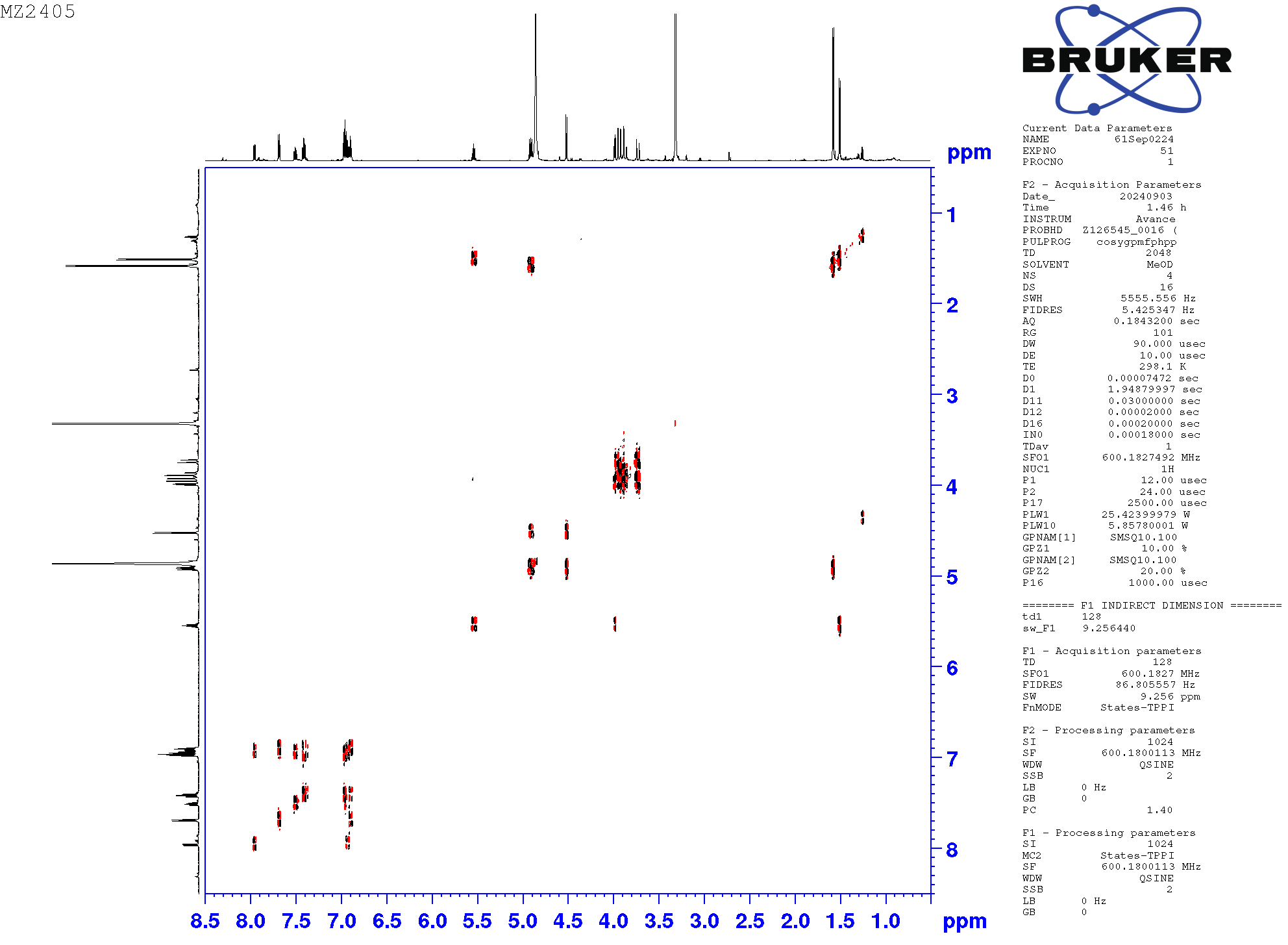


**Supplementary Figure S6.** COSY spectrum of kineochelin E_1_ (**1**) in CD_3_OH at 600 MHz.


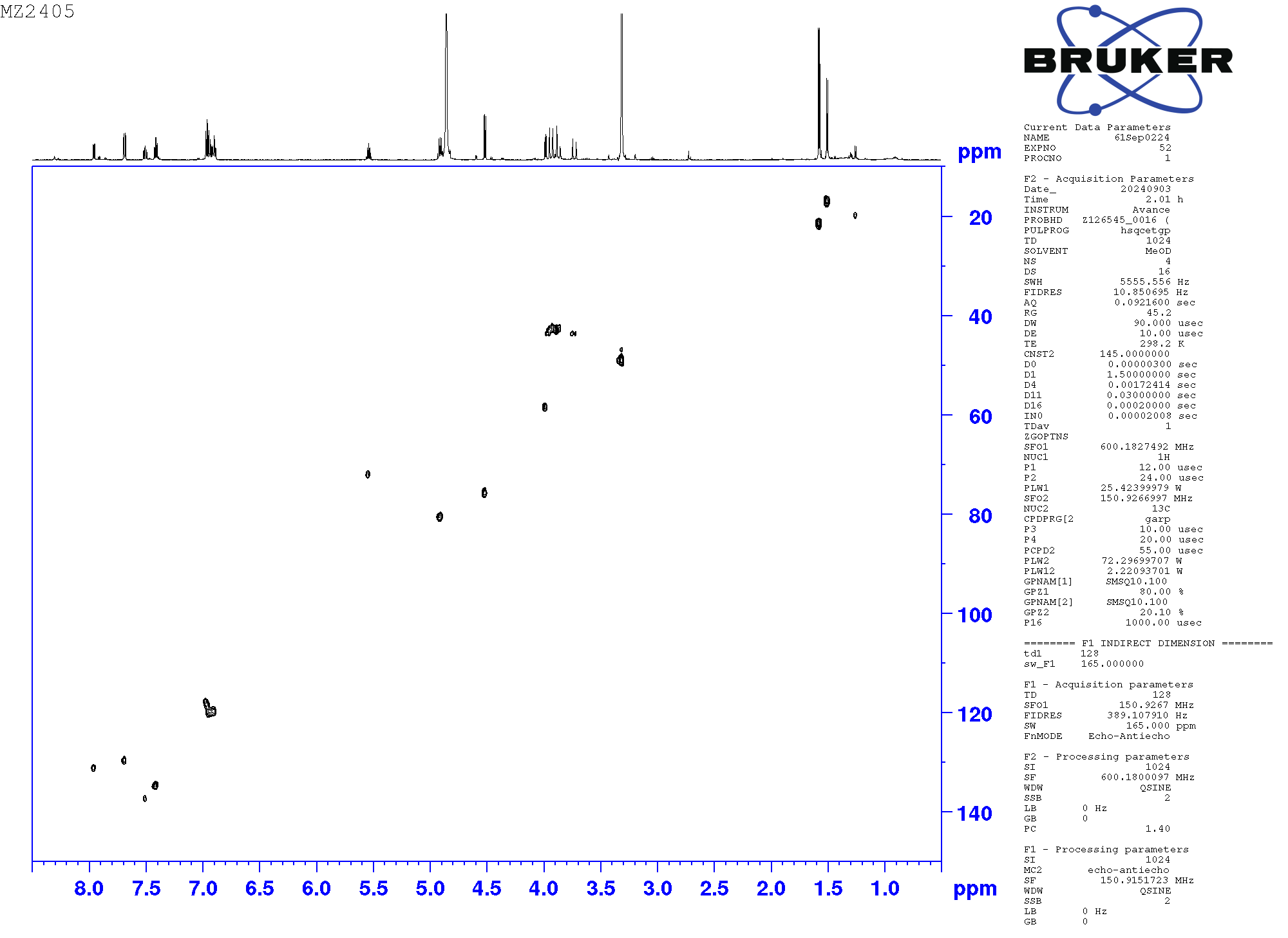


**Supplementary Figure S7.** HSQC spectrum of kineochelin E_1_ (**1**) in CD_3_OH at 600 MHz.


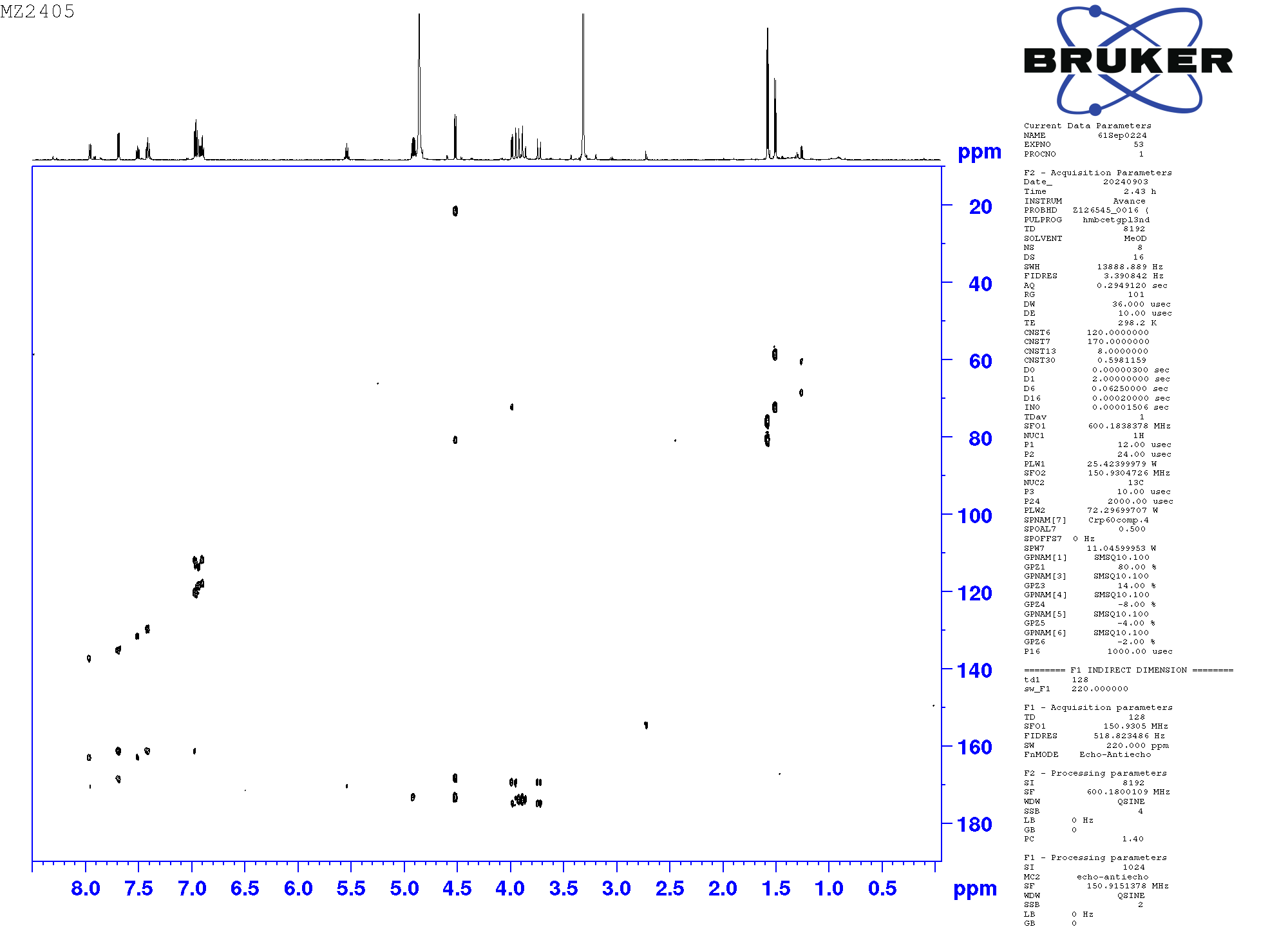


**Supplementary Figure S8.** HMBC spectrum of kineochelin E_1_ (**1**) in CD_3_OH at 600 MHz.

**Supplementary Figure S9.** Marfey’s analysis of kineochelin E_1_ and kineochelin A_1_. Extracted ion chromatograms (*m/z* 372.1150±0.0050) showing the signals for L-FDAA-derivatised free amino acids L-Thr, D-Thr, D-*allo*-Thr, and L-*allo*-Thr, as well as of L-Thr in the hydrolysed fraction F16, containing mainly kineochelin E_1_, and hydrolysed fraction F10, containing mainly kineochelin A_1_. The bottom two EICs show the standard addition experiments confirming presence of pure L-Thr in kineochelin E_1_ and kineochelin A_1_.

**Supplementary Figure S10.** Purity of isolated kineochelin A_1_. UHPLC-ELSD chromatogram of fraction 11 (5.8 mg) containing kineochelin A_1_ (2) as main compound.


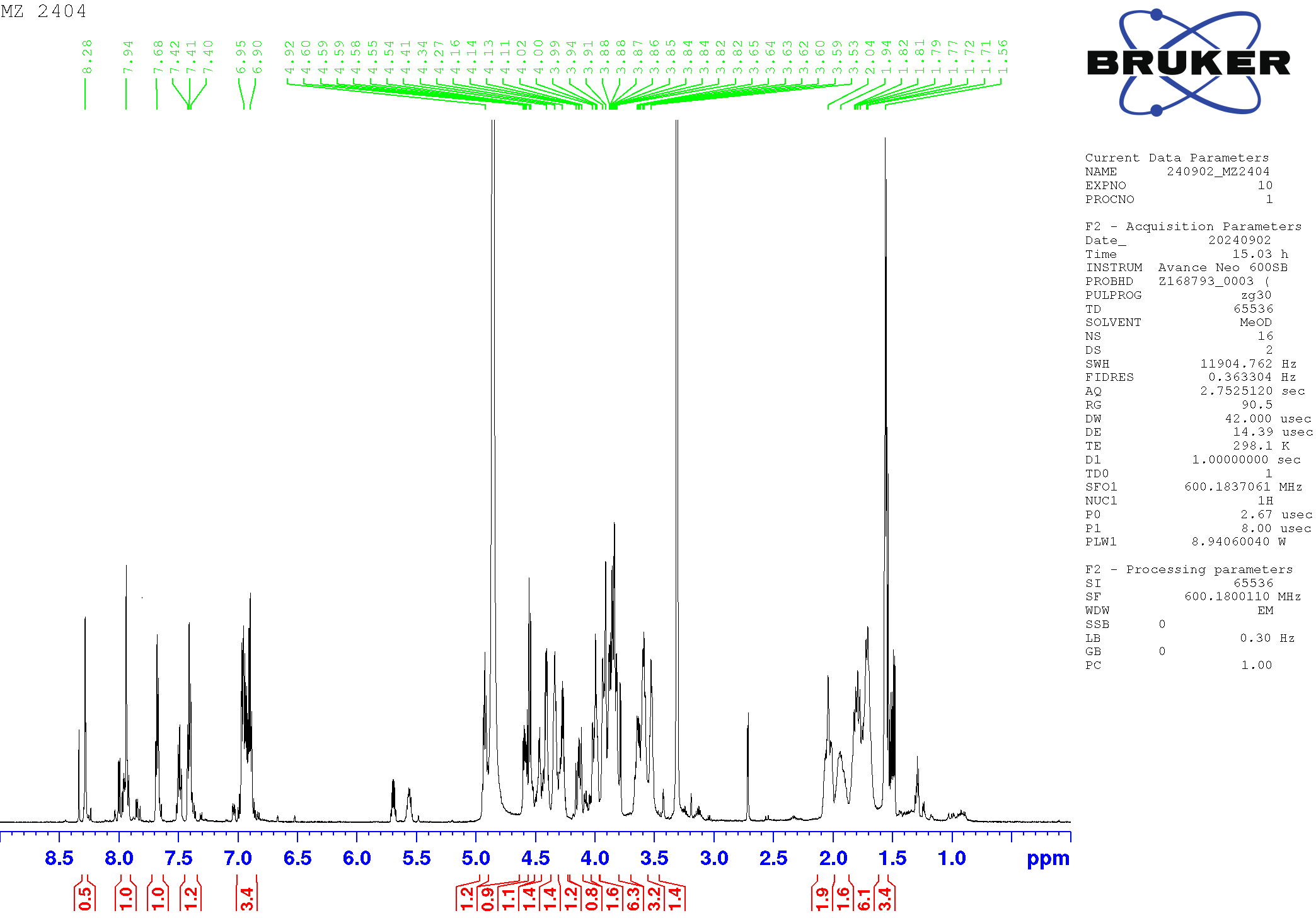


**Supplementary Figure S11.** ^1^H NMR spectrum of kineochelin A_1_ (**2**) in CD_3_OH at 600 MHz.


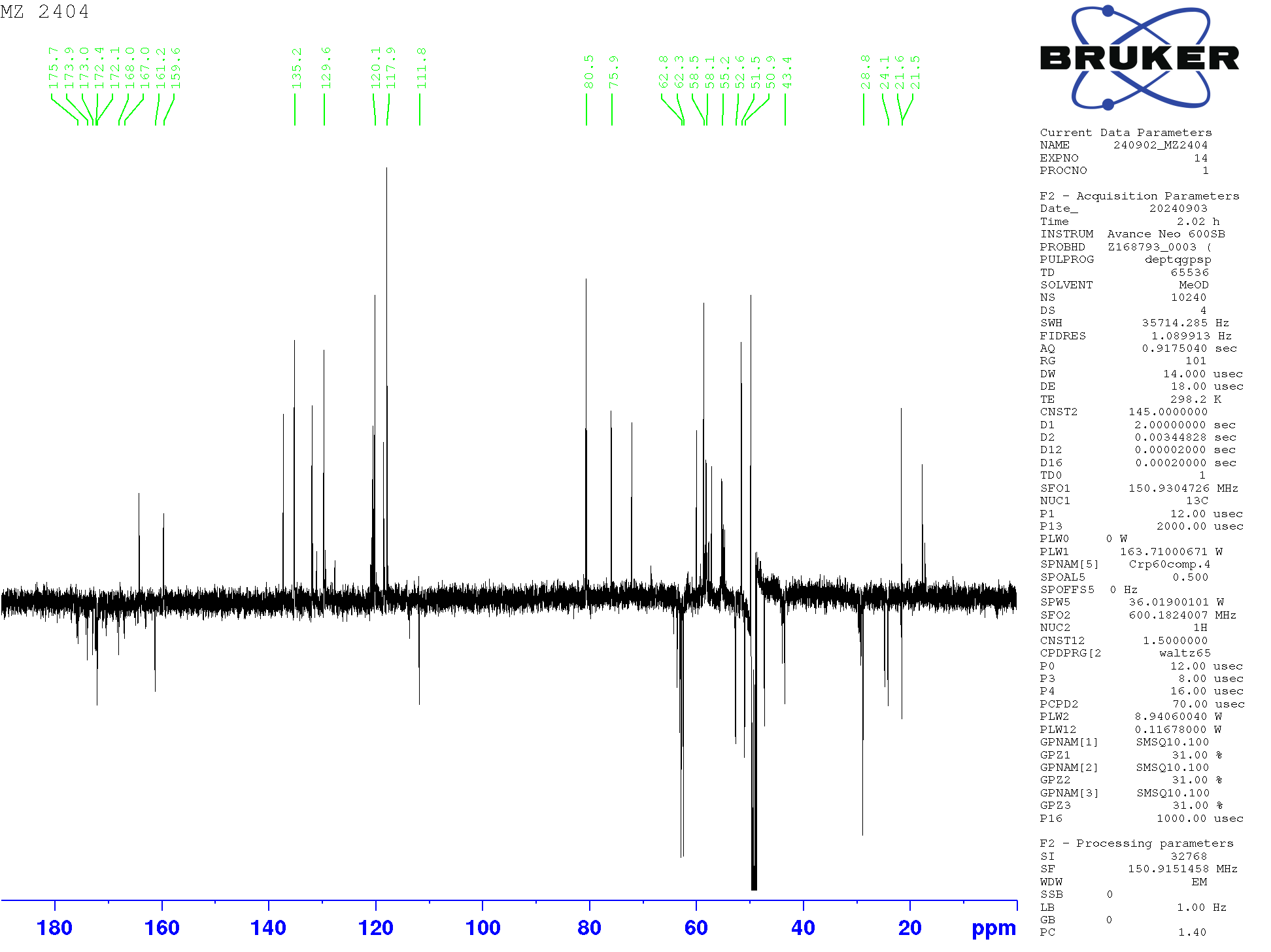


**Supplementary Figure S12.** ^13^C (DEPTq) NMR spectrum of kineochelin A_1_ (**2**) in CD_3_OH at 151 MHz.


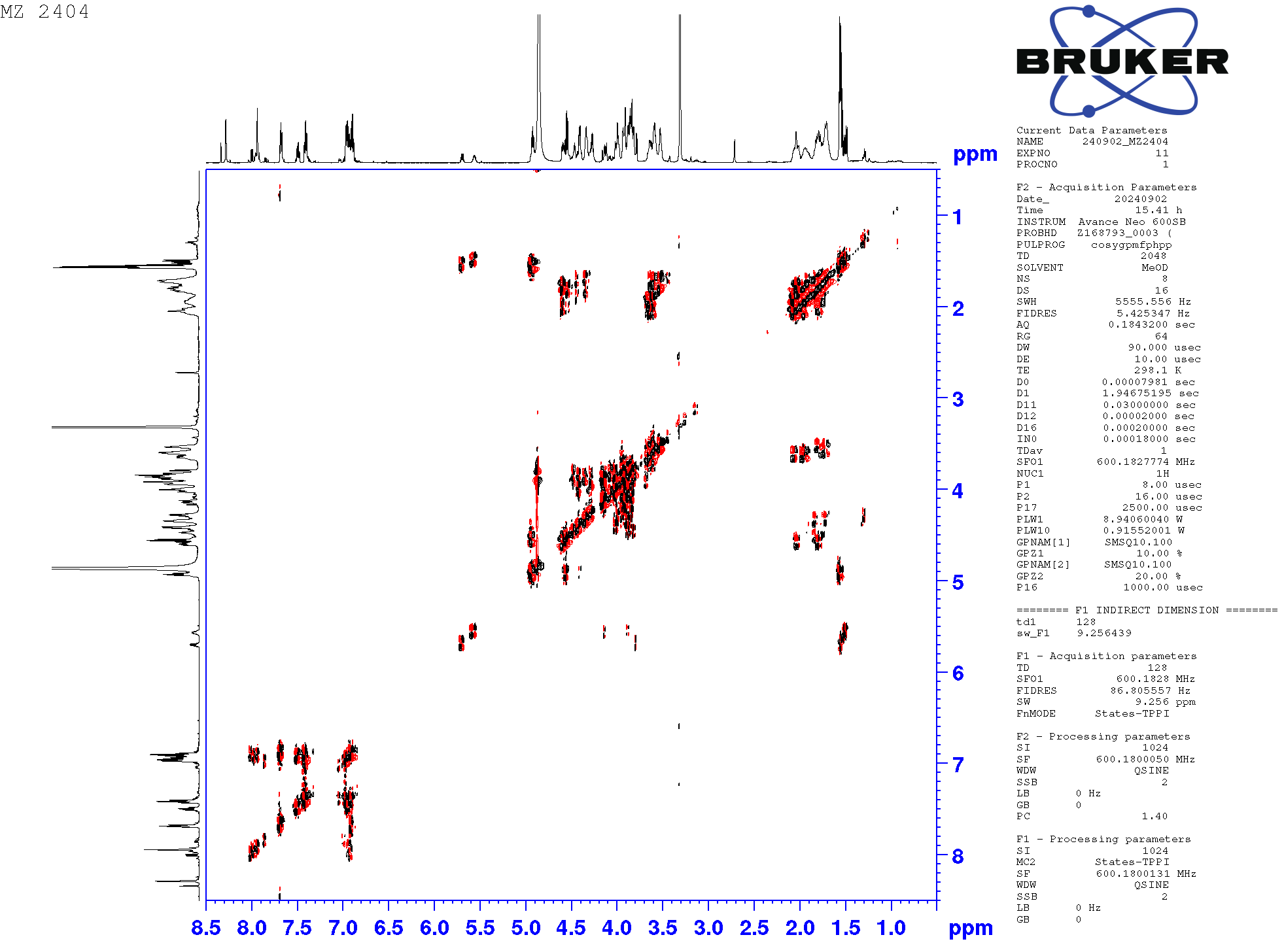


**Supplementary Figure S13.** COSY spectrum of kineochelin A_1_ (**2**) in CD_3_OH at 600 MHz.


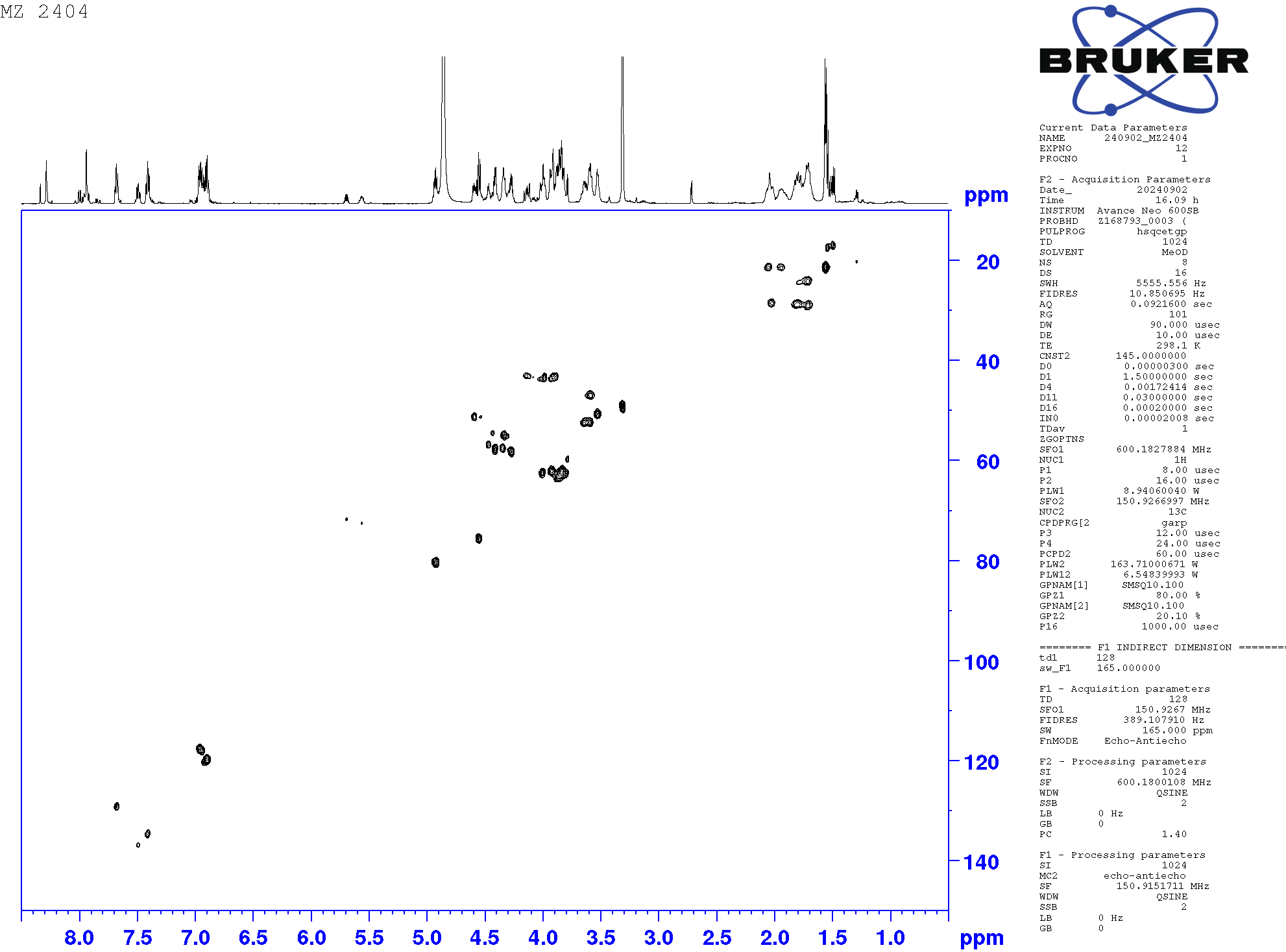


**Supplementary Figure S14.** HSQC spectrum of kineochelin A_1_ (**2**) in CD_3_OH at 600 MHz.


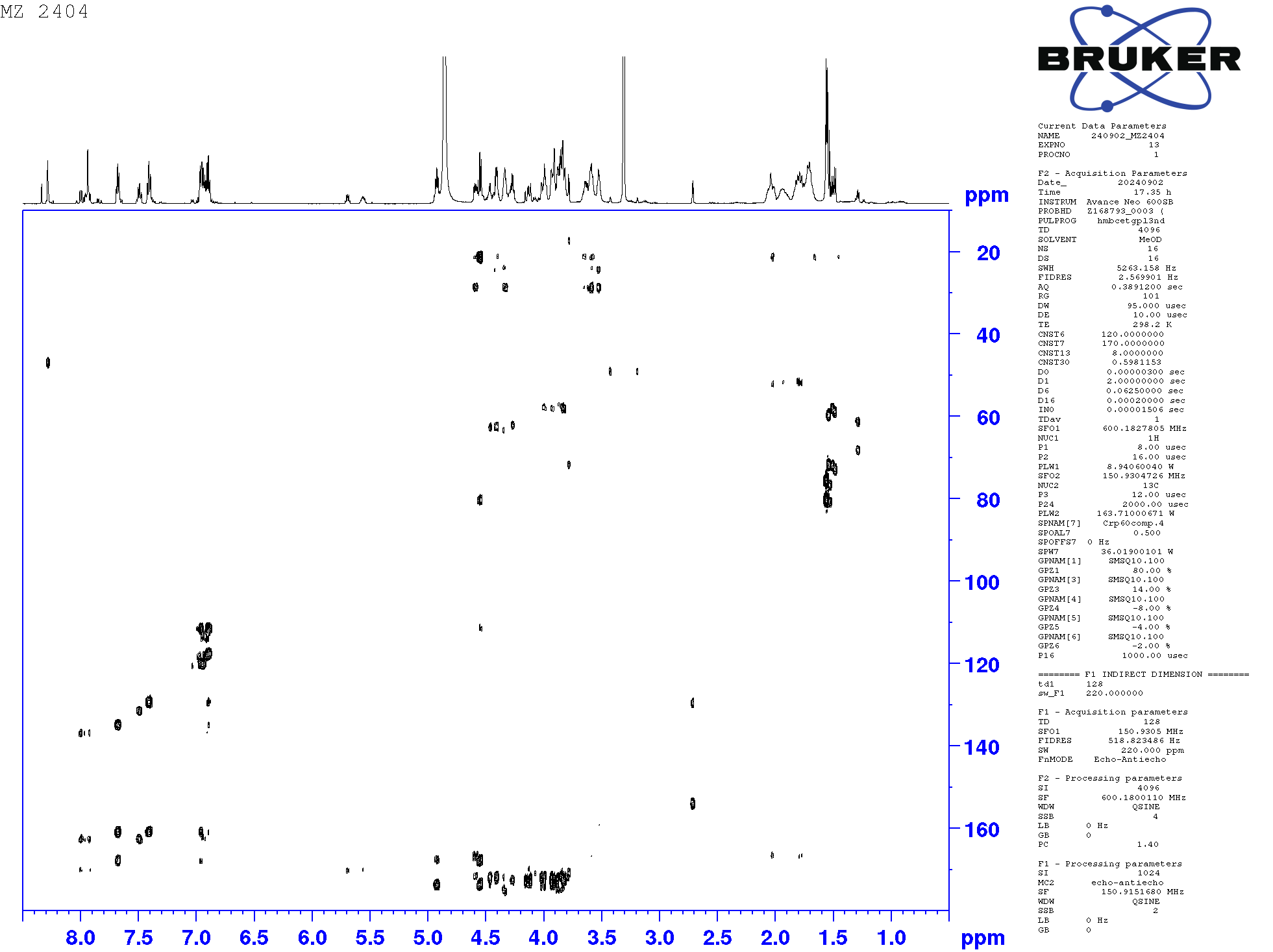


**Supplementary Figure S15.** HMBC spectrum of kineochelin A_1_ (**2**) in CD_3_OH at 600 MHz.

**Supplementary Figure S16.** High resolution ESI-Qq-TOF mass spectrum of kineochelin A_1_ (A) and high resolution MS/MS spectrum of its [M+H]^+^ ion (B).

**Supplementary Figure S17.** High resolution ESI-Qq-TOF mass spectrum of kineochelin A_2_ (A) and high resolution MS/MS spectrum of its [M+H]^+^ ion (B).

**Supplementary Figure S18.** High resolution ESI-Qq-TOF mass spectrum of kineochelin B_1_ (A) and high resolution MS/MS spectrum of its [M+H]^+^ ion (B).

**Supplementary Figure S19.** High resolution ESI-Qq-TOF mass spectrum of kineochelin B_2_ (A) and high resolution MS/MS spectrum of its [M+H]^+^ ion (B).

**Supplementary Figure S20.** High resolution ESI-Qq-TOF mass spectrum of kineochelin C_1_ (A) and high resolution MS/MS spectrum of its [M+H]^+^ ion (B).

**Supplementary Figure S21.** High resolution ESI-Qq-TOF mass spectrum of kineochelin D_1_ (A) and high resolution MS/MS spectrum of its [M+H]^+^ ion (B).

**Supplementary Figure S22.** High resolution ESI-Qq-TOF mass spectrum of kineochelin E_1_ (A) and high resolution MS/MS spectrum of its [M+H]^+^ ion (B).

**Supplementary Figure S23.** High resolution ESI-Qq-TOF mass spectrum of kineochelin E_2_ (A) and high resolution MS/MS spectrum of its [M+H]^+^ ion (B).

**Supplementary Figure S24.** High resolution ESI-Qq-TOF mass spectrum of vulnibactin 2 (A) and high resolution MS/MS spectrum of its [M+H]^+^ ion (B).

**Supplementary Figure S25.** High resolution ESI-Qq-TOF mass spectrum of pseudomobactin A (A) and high resolution MS/MS spectrum of its [M+H]^+^ ion (B).

**Supplementary Figure S26.** High resolution ESI-Qq-TOF mass spectrum of asteroidic acid (A) and high resolution MS/MS spectrum of its [M+H]^+^ ion (B).

**Supplementary Figure S27. Marfey’s analysis of kineochelin A_1_.** Extracted ion chromatograms (*m/z* 358.0993±0.0050, red; *m/z* 385.1466±0.0050, purple; *m/z* 637.1961±0.0050, blue) showing the signals for L-FDAA-derivatised free amino acids L-Ser, D-Ser, L-Orn, and D-Orn, as well as of D-Ser and L-Orn in the hydrolysed fraction F10, containing mainly kineochelin A_1_.

**Figure S28.** Planar structures of cyclo(Hyp-Leu) (**3**) and cyclo(Hyp-Phe) (**4**) with atom numbering and ^1^H-^1^H COSY and key ^1^H-^13^C HMBC correlations.


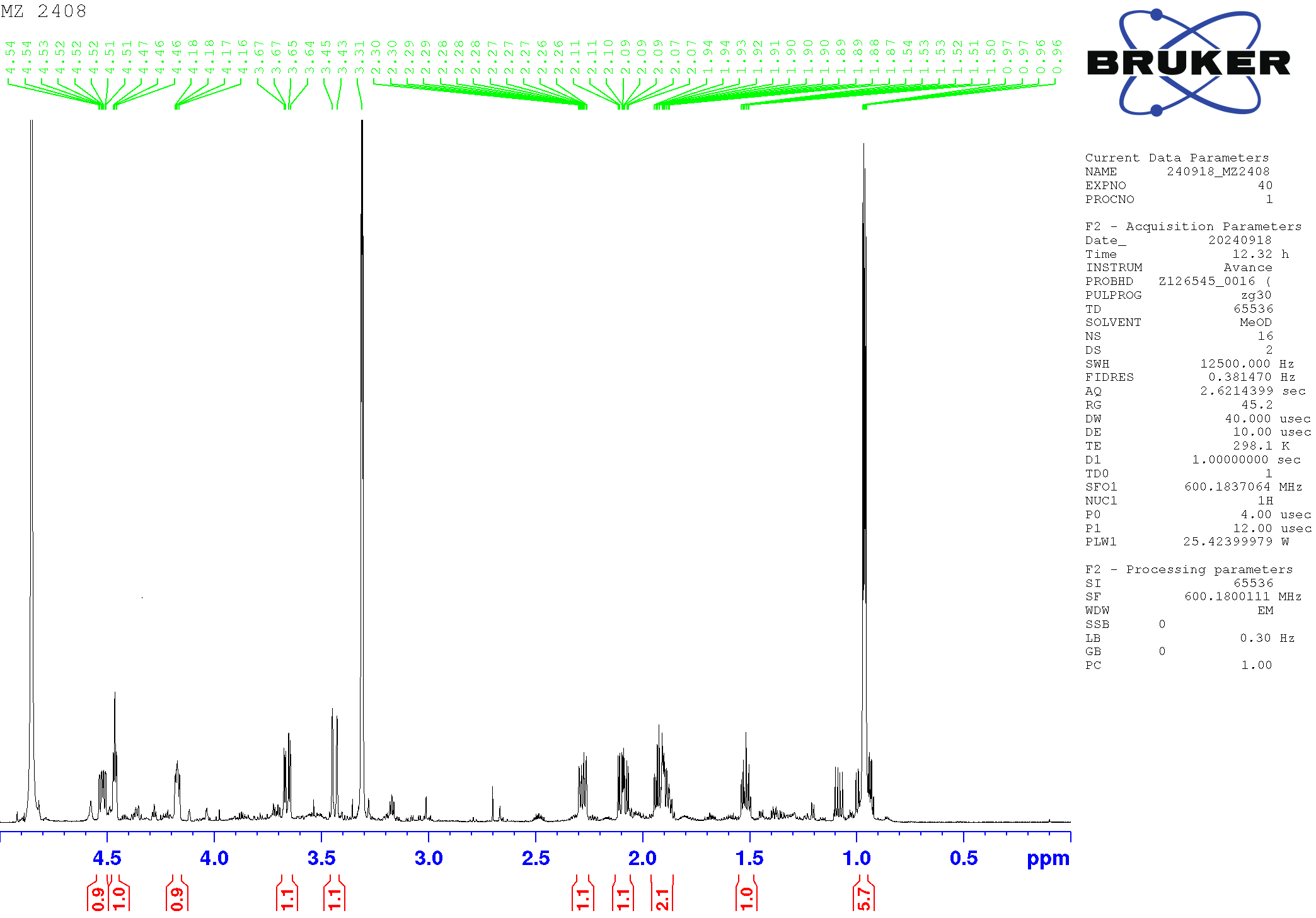


**Figure S29.** ^1^H NMR spectrum of cyclo(Hyp-Leu) (**3**) in CD_3_OH at 600 MHz.


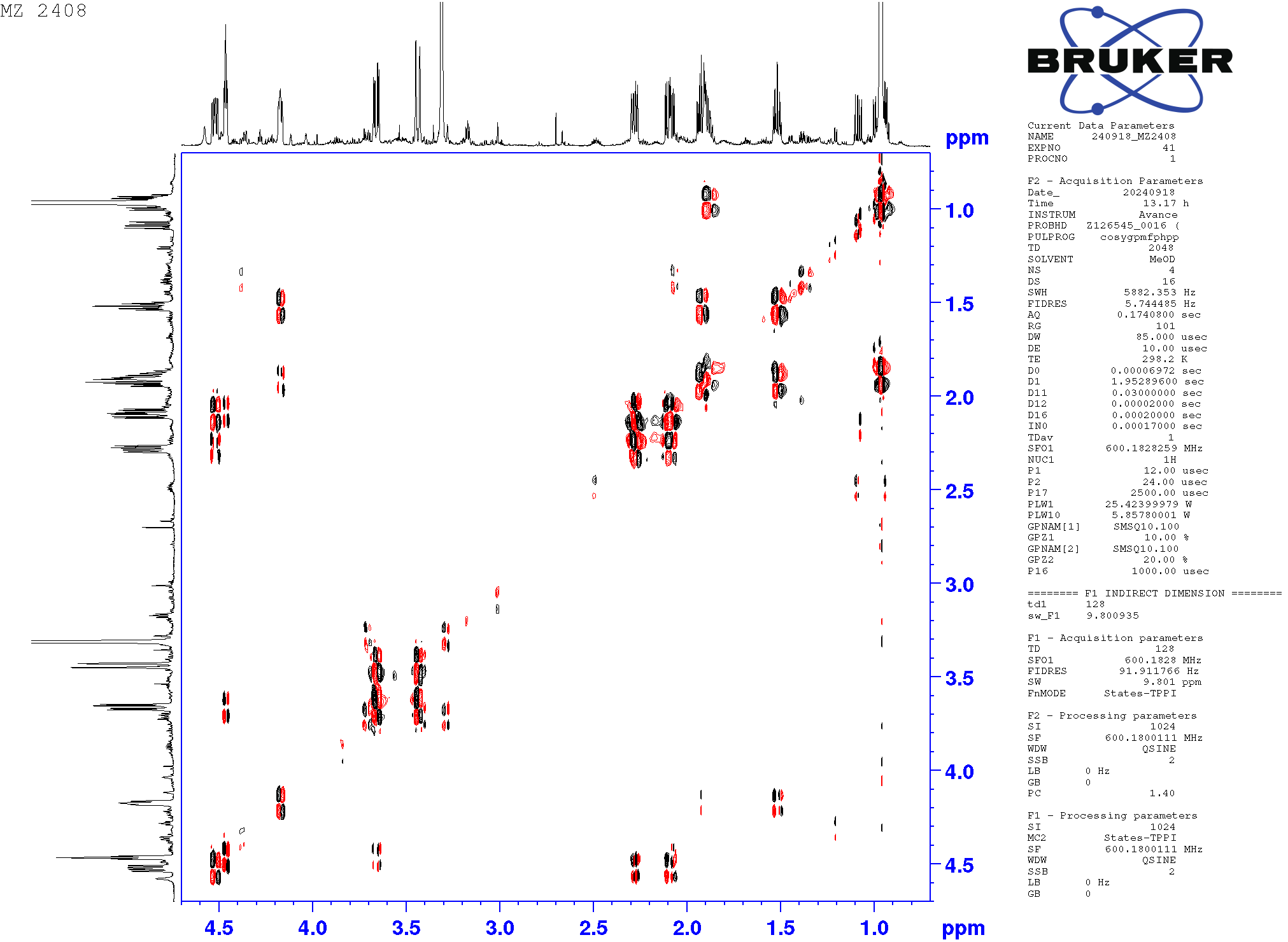


**Figure S30.** COSY spectrum of cyclo(Hyp-Leu) (**3**) in CD_3_OH at 600 MHz.


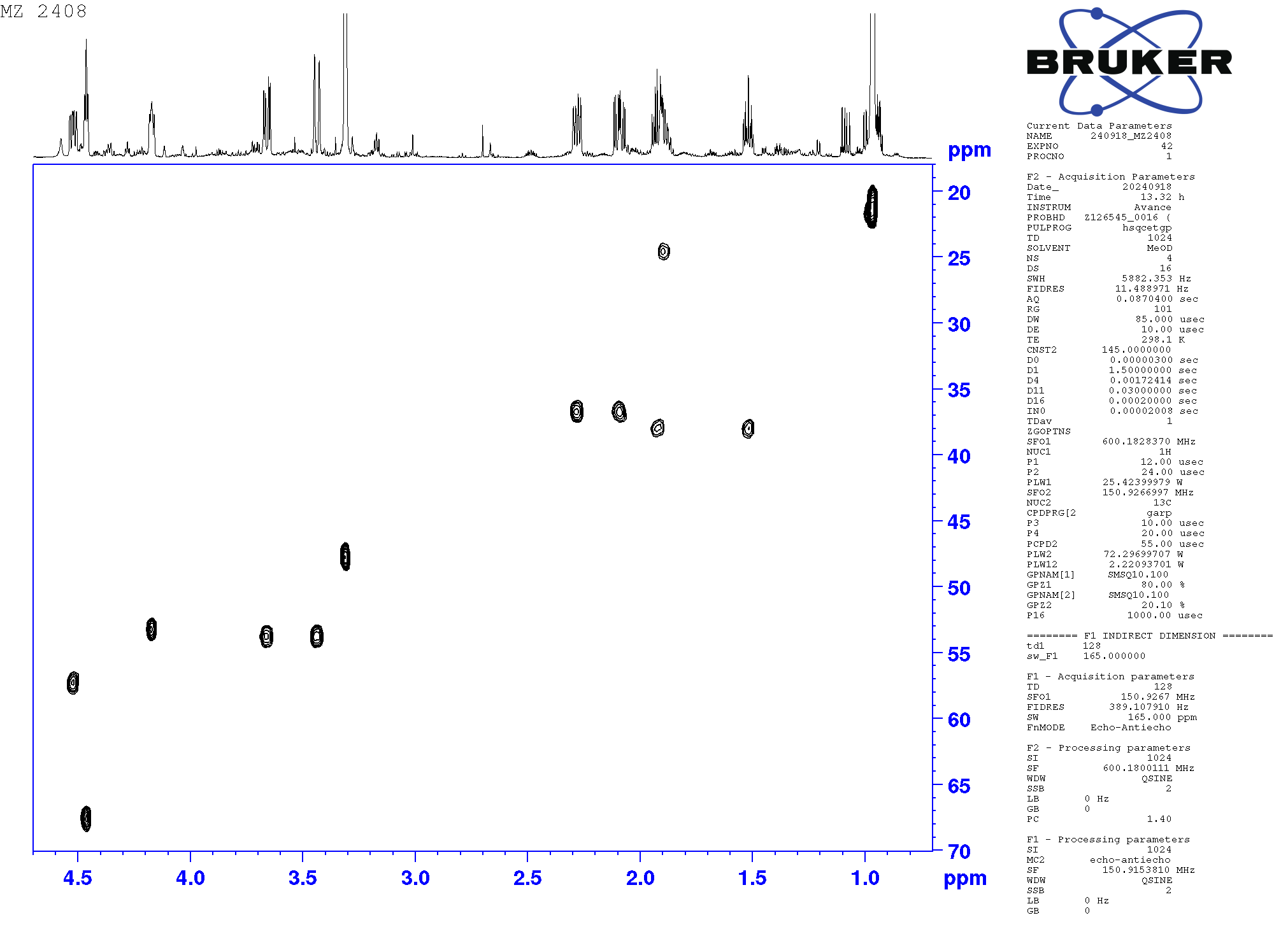


**Figure S31.** HSQC spectrum of cyclo(Hyp-Leu) (**3**) in CD_3_OH at 600 MHz.


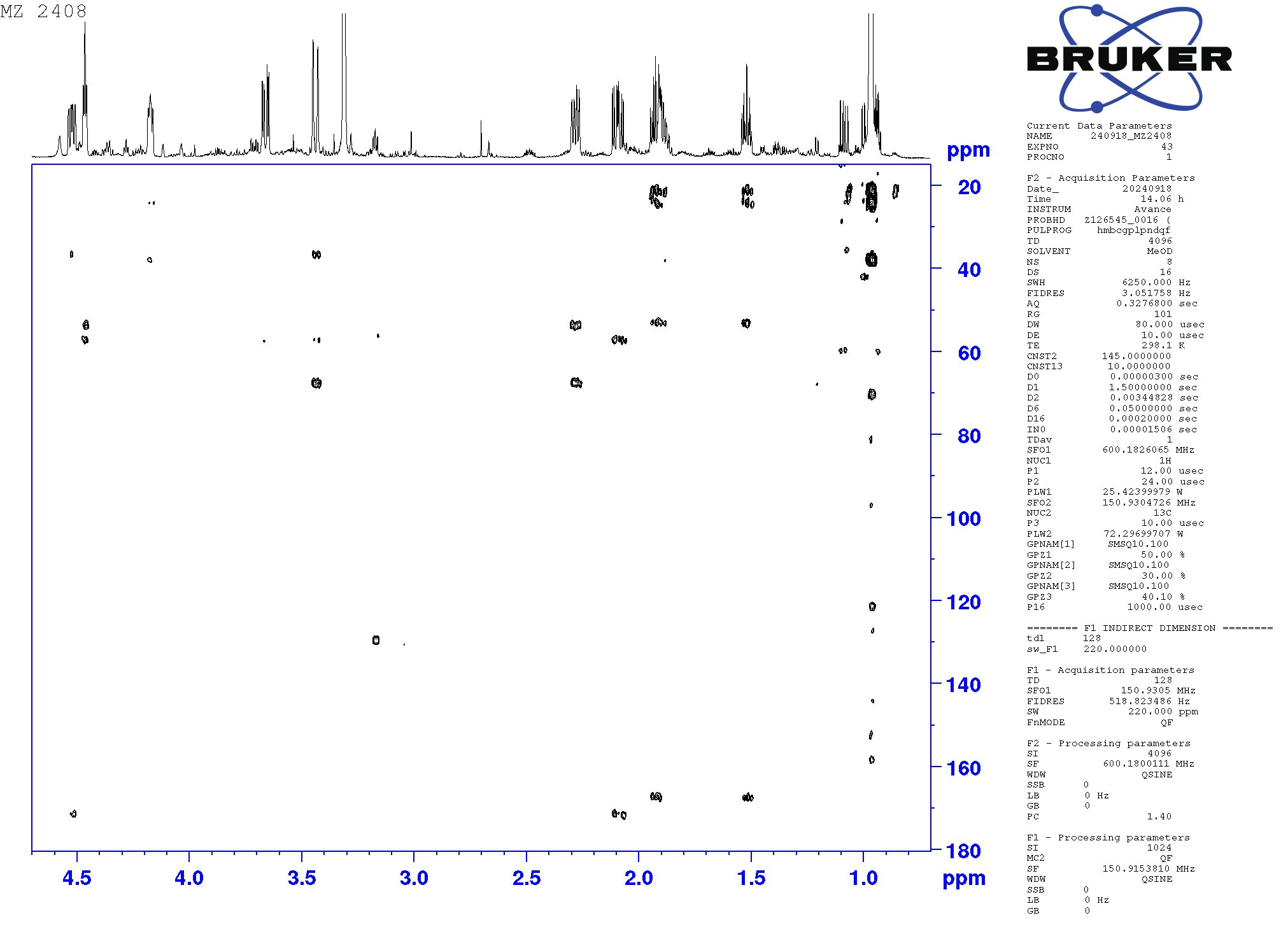


**Figure S32.** HMBC spectrum of cyclo(Hyp-Leu) (**3**) in CD_3_OH at 600 MHz.


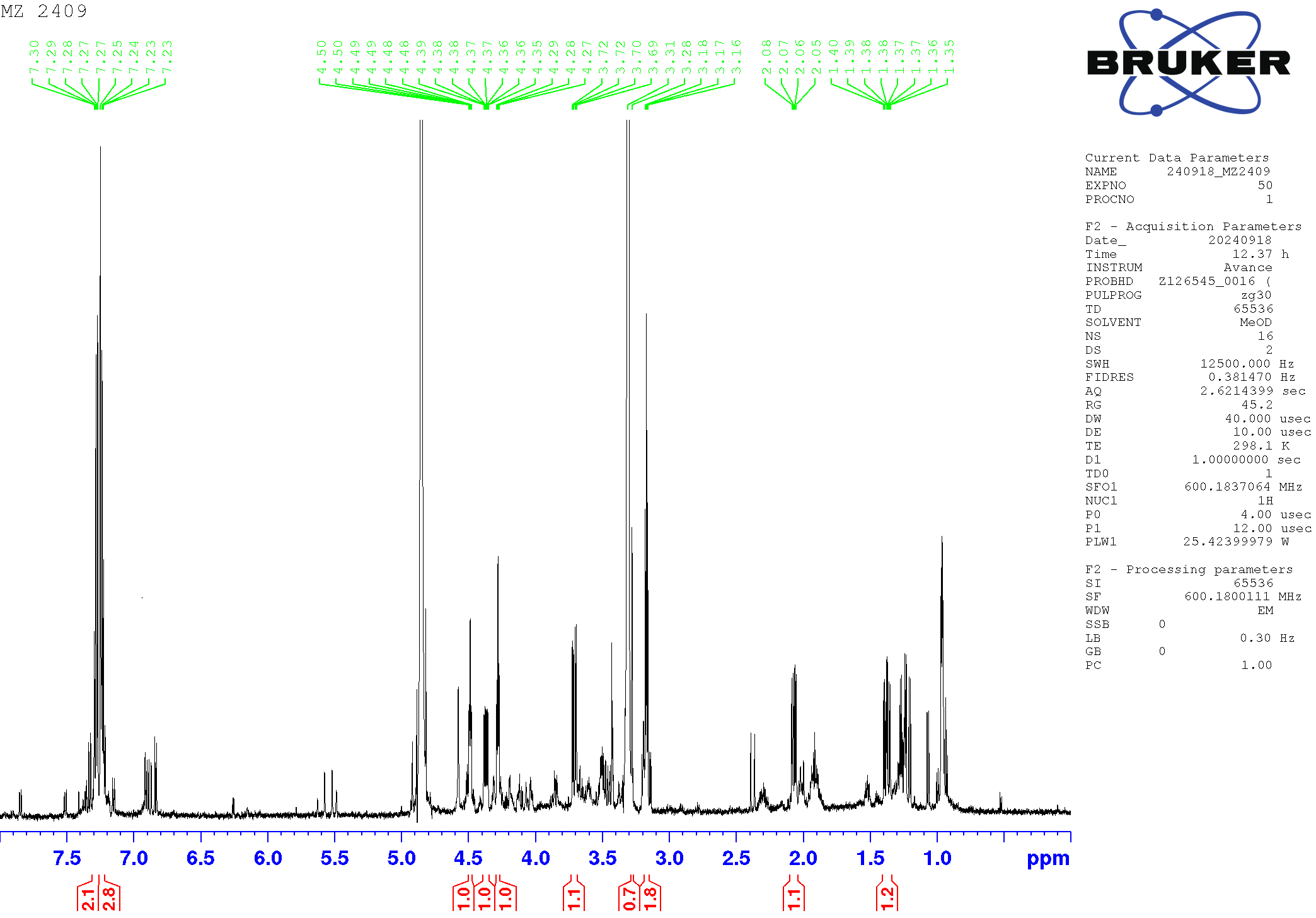


**Figure S33.** ^1^H NMR spectrum of cyclo(Hyp-Phe) (**4**) in CD_3_OH at 600 MHz.


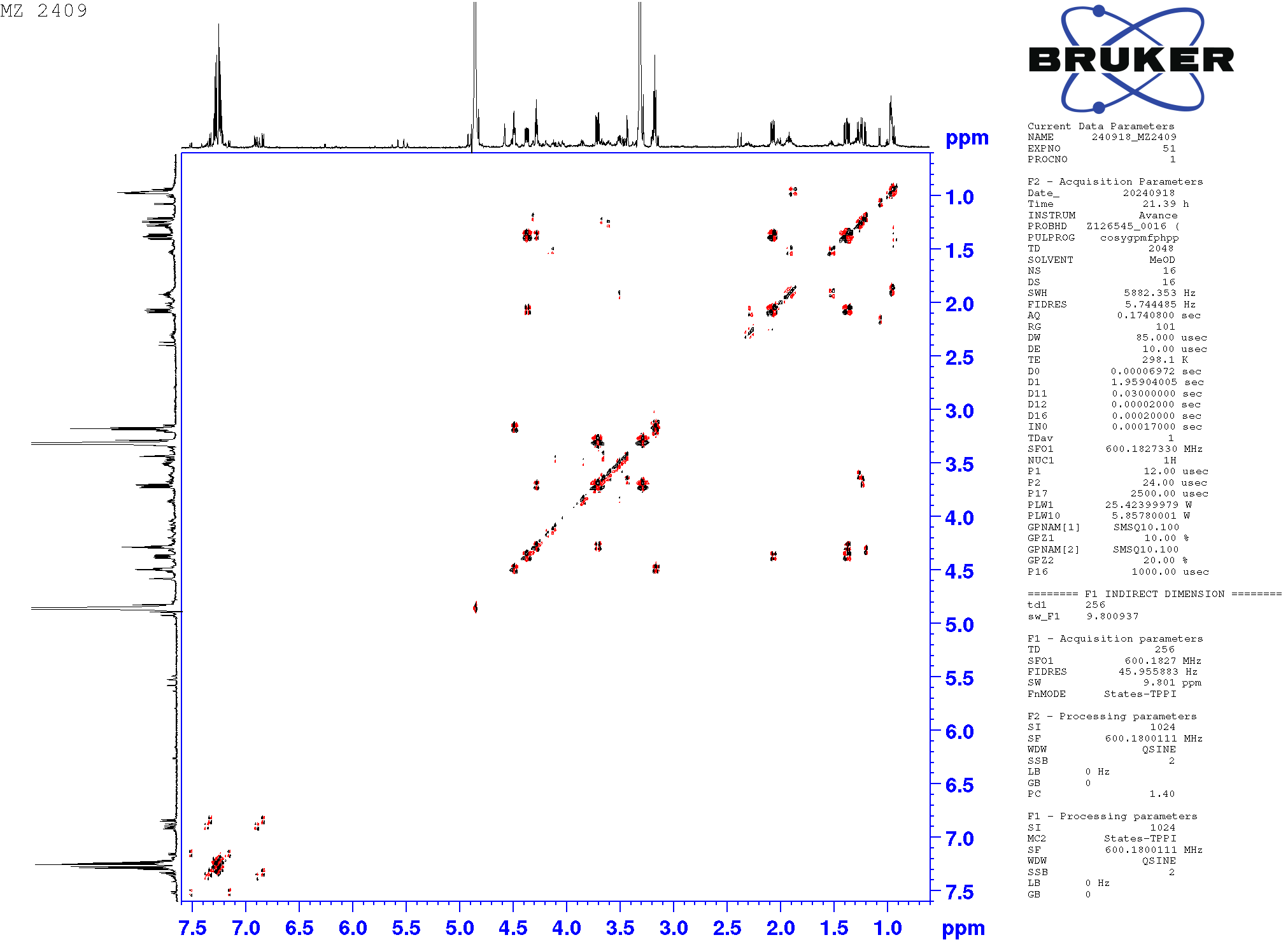


**Figure S34.** COSY spectrum of cyclo(Hyp-Phe) (**4**) in CD_3_OH at 600 MHz.


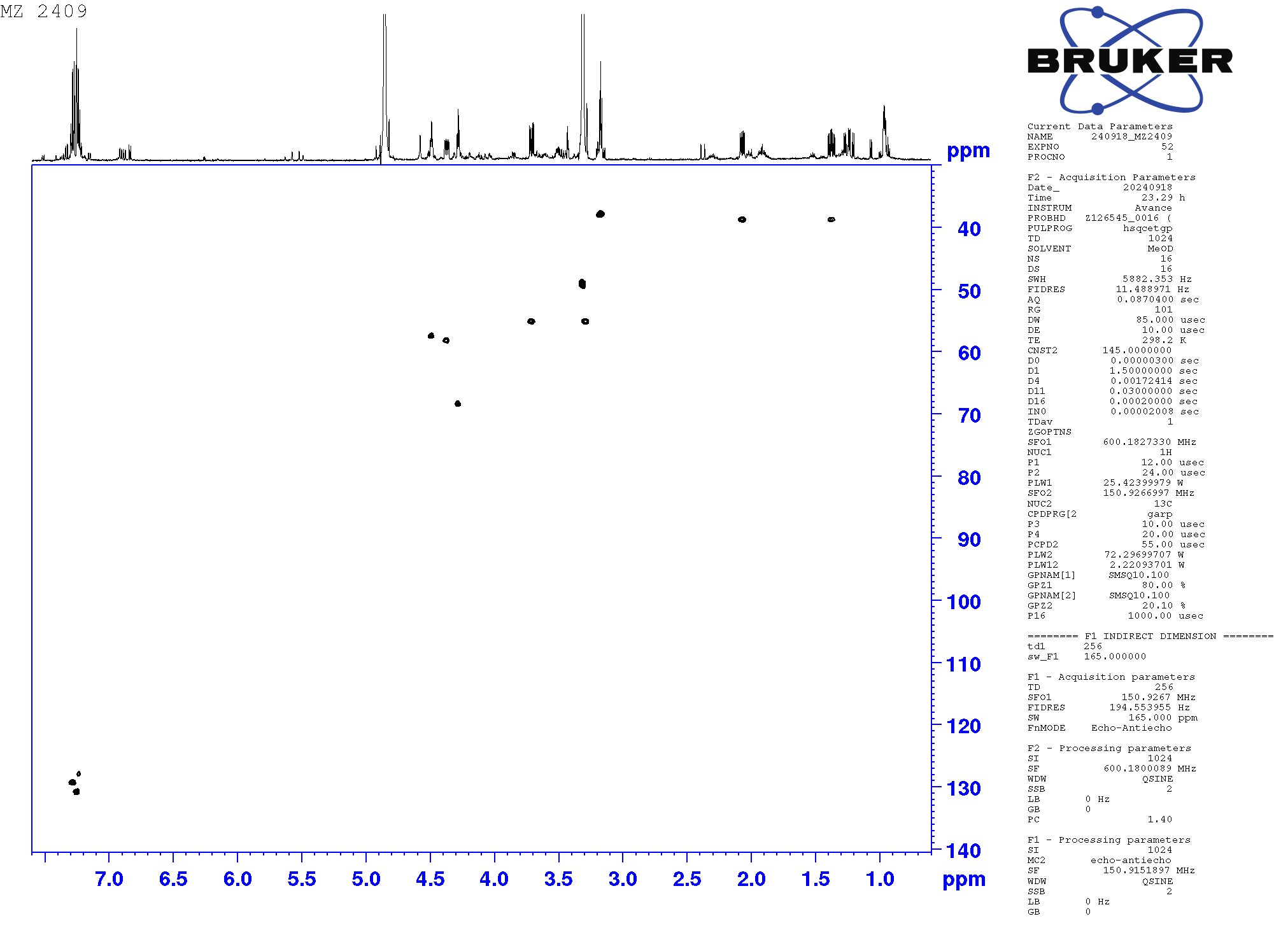


**Figure S35.** HSQC spectrum of cyclo(Hyp-Phe) (**4**) in CD_3_OH at 600 MHz.


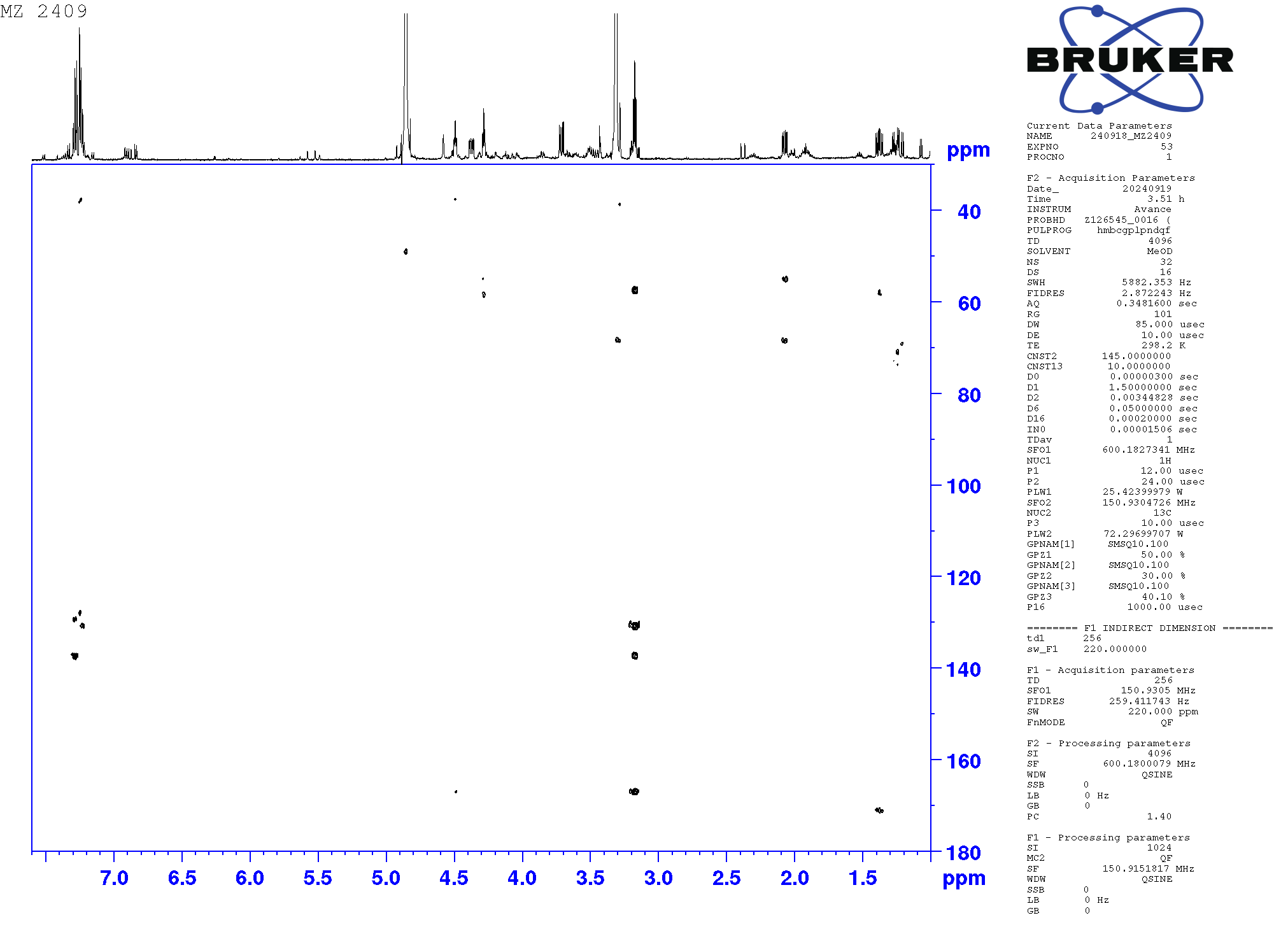


**Figure S36.** HMBC spectrum of cyclo(Hyp-Phe) (**4**) in CD_3_OH at 600 MHz.

**
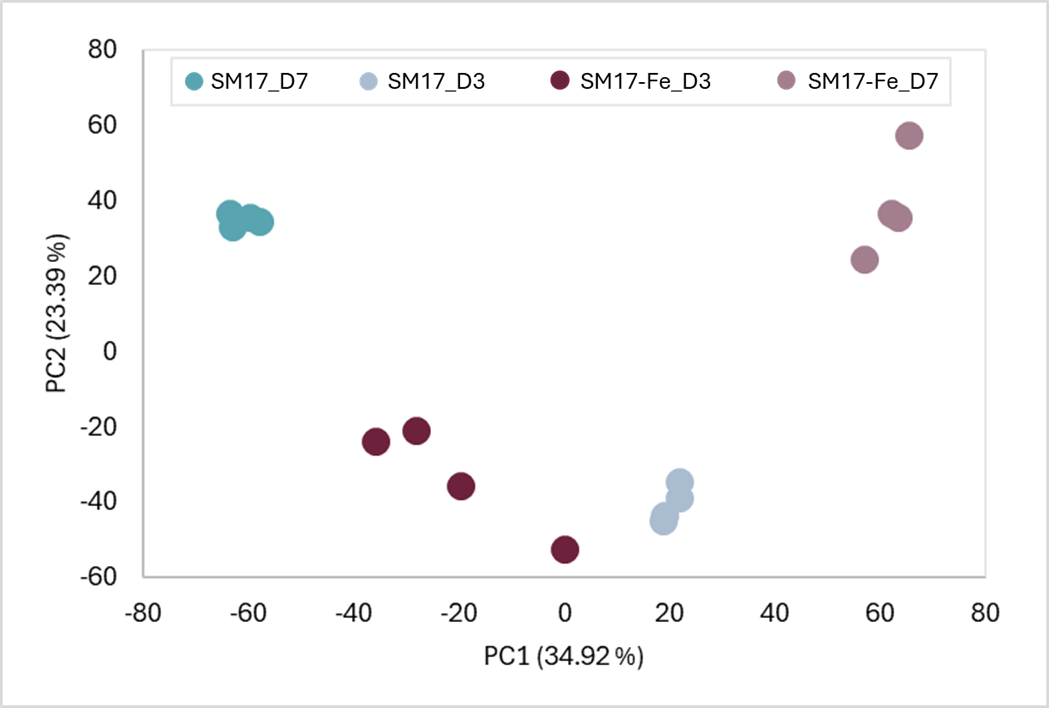
**

**Supplementary Figure S37. Principal component analysis (PCA) of RNA-seq samples.** PCA of normalized transcript counts from four conditions (SM17 vs. SM17 + 200 µM FeCl₃ at day 3 and day 7). Samples separate cleanly (PC1 = 34.9%, PC2 = 21.3%); PC1 distinguishes medium and PC2 captures the time shift. Biological replicates cluster tightly within each group.


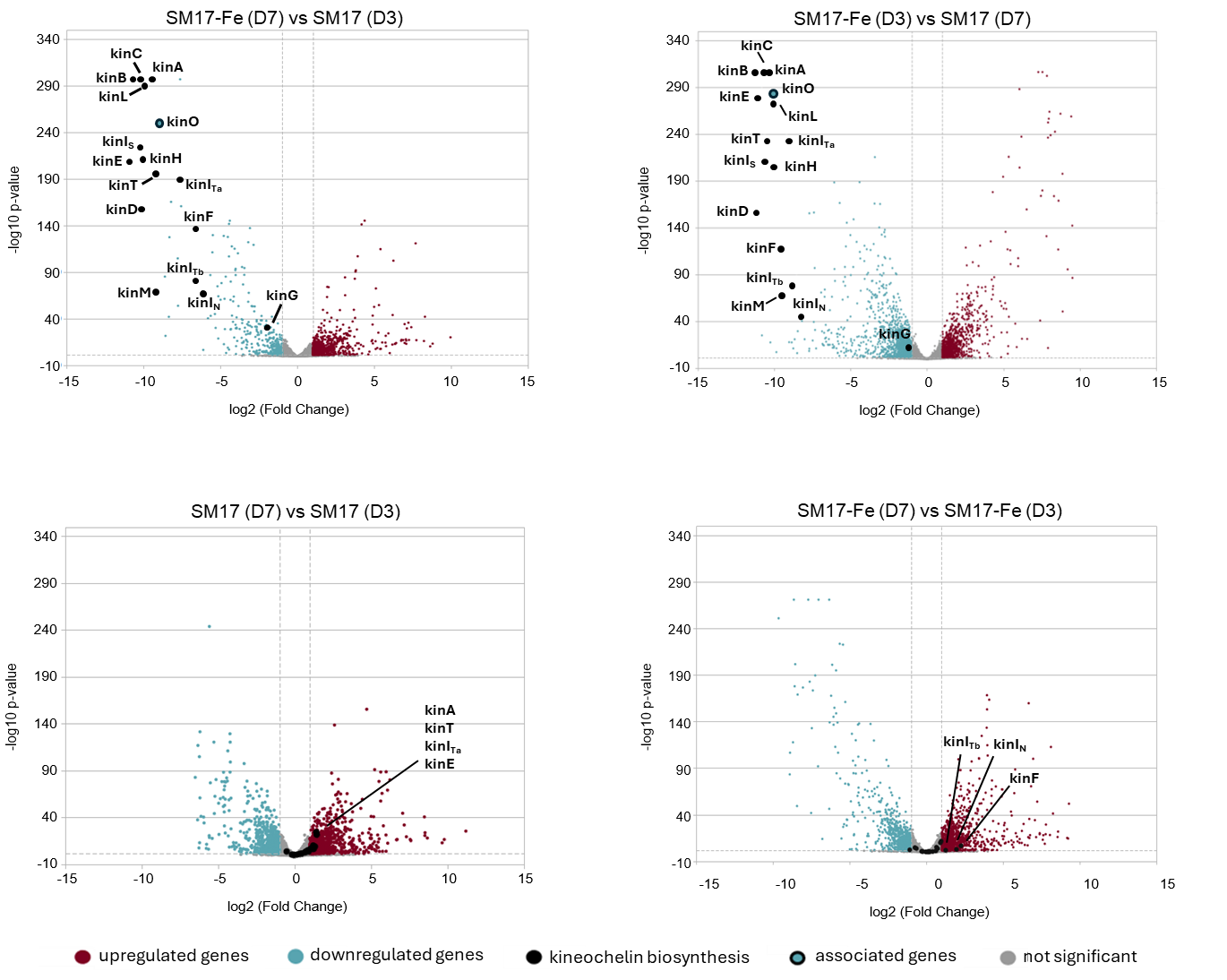


**Supplementary Figure S38. Comparative transcriptomics of *Actinokineospora* sp. UV203 under siderophore-producing and siderophore-depleted growth conditions.** Volcano plots for the indicated pairwise contrasts. Each point is a gene (x-axis: log₂ fold change; y-axis: −log₁₀(FDR)); dashed lines mark significance thresholds (|log₂FC| ≥ 1 and FDR < 0.05). PCA and volcano plots were generated based on normalized counts from DESeq.

**Supplementary Figure S39.** LC-MS base peak chromatogram of the pre-purified butanol-phase extract showing the kineochelin mixture and co-purified known shunt products.

**
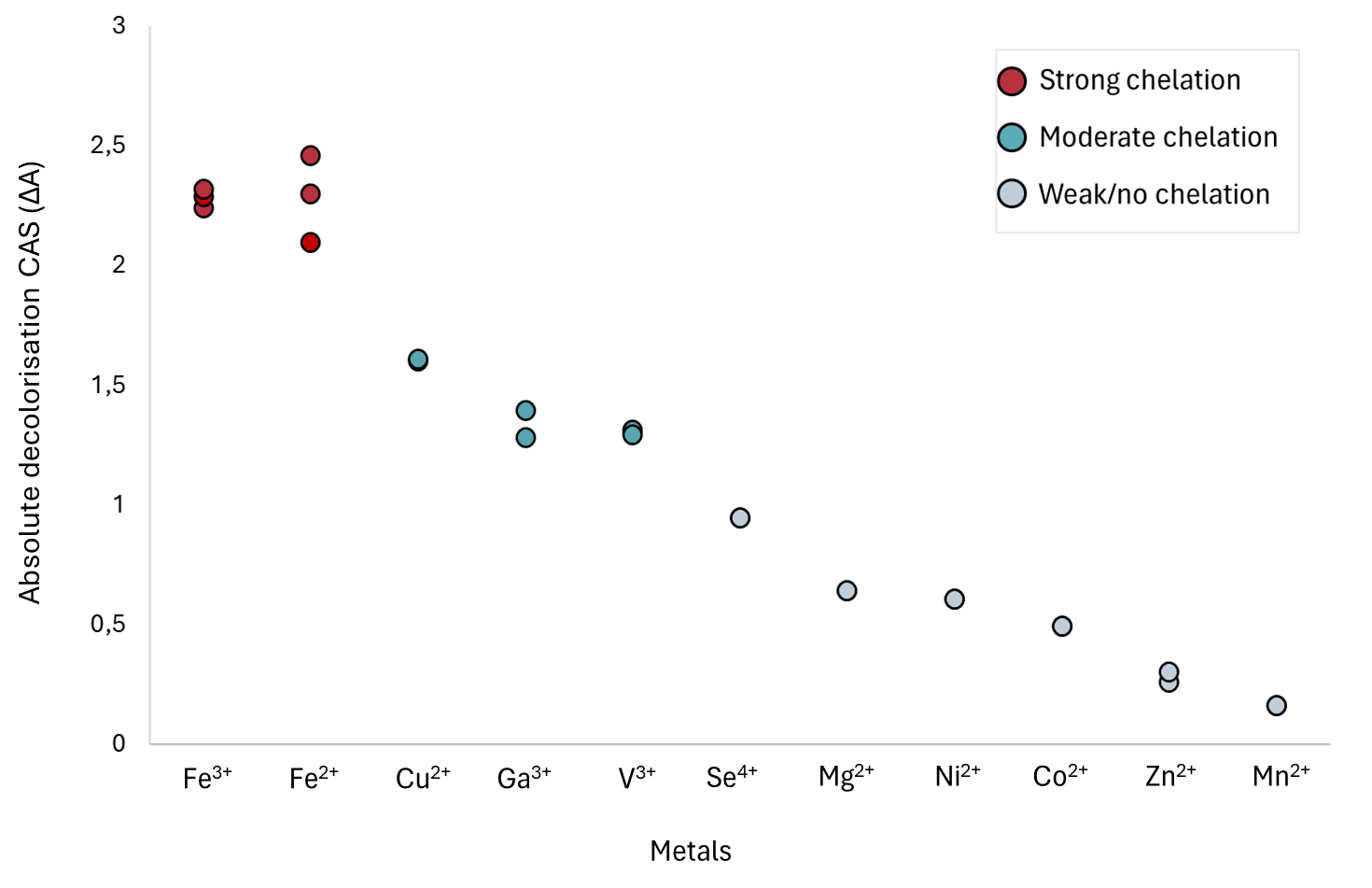
**

**Supplementary Figure S40. Metal-binding activity of UV203 SM17 culture supernatant measured by Chrome Azurol S (CAS)-shuttle assay.**Absolute CAS decolorization (ΔA) values are shown for individual metal–CAS complexes. Points represent technical replicates (n = 3), and colours indicate relative binding strength normalized to the Fe³⁺ response.

**Supplementary Table S1.** Results from the untargeted LC-MS-based secondary metabolomics analysis of *Actinokineospora* sp. UV203 grown in different media. Groups of secondary metabolites known or presumed to be biosynthetically related are highlighted by the same colour, whereby usually only the most abundant congeners are reported. The listed isoflavone derivatives were only found in cultures from soy-containing media, but not in the media controls. They are thus assumed to be biotransformation products from the soy-isoflavones present in these media.

**Supplementary Table S2.** ^1^H (600 MHz) and ^13^C NMR data (151 MHz) of kineochelin E_1_ (**1**) in CD_3_OD in comparison with literature data for pseudomobactin A (*δ* in ppm). A second set of signals (**1’**) was observed in a ratio of approximately 3:5 to the one shown in Table 2. This set of signals was initially hypothesized to belong to a stereoisomer of **1**, but after Marfey’s analysis is now assumed to belong to a stable conformer or metal ion complex of kineochelin E_1_.

|  | **Kineochelin E_1_ (1)** | | **Kineochelin E_1_ (1’)** | | **Pseudomobactin A^a^** | |
| --- | --- | --- | --- | --- | --- | --- |
| **Position** | δ_H_ (*J* in Hz) | δ_C_, type | δ_H_ (*J* in Hz) | δ_C_, type | δ_H_ (*J* in Hz) | δ_C_, type |
| **1** | — | 161.3, C | — | 163.0, C | — | 161.1, C |
| **2** | 6.96, m, ov | 117.9, CH | 6.89-6.97, m, ov | 118.5, CH | 6.96, d (8.3) | 117.7, CH |
| **3** | 7.41, m | 135.2, CH | 7.51, m | 137.4, CH | 7.41, td (8.3, 1.7) | 135.0, CH |
| **4** | 6.90, m, ov | 120.1, CH | 6.89-6.97, m, ov | 120.6, CH | 6.90, td (7.4, 1.7) | 119.9, CH |
| **5** | 7.69, dd (7.9, 1.7) | 129.7, CH | 7.96, dd (8.0, 1.6) | 131.7, CH | 7.68, dd (7.4, 1.7) | 129.5, CH |
| **6** | — | 111.8, C | — | 113.6, C | — | 111.6, C |
| **7** | — | 168.3, C | — | 170.4, C | — | 167.8, C |
| **8** | 1.57, d (6.3) | 21.6, CH_3_ | 1.50, d (6.5) | 17.1, CH3 | 1.57, d (6.3) | 21.4, CH_3_ |
| **9** | 4.91, dq (7.5, 6.3) | 80.8, CH | 5.54, qd (6.3, 6.3) | 72.3, CH | 4.90, qd (7.3, 6.3) | 80.6, CH |
| **10** | 4.51, d (7.5) | 75.9, CH | 3.98, d (6.0), ov | 58.7, CH | 4.46, d (7.3) | 75.5, CH |
| **11** | — | 173.3, C | — | 169.2, C | — | 175.6, C |
| **12a** | 3.93, d (17.0), ov | 43.0, CH_2_ | 3.96, d (17.3), ov | 43.8, CH2 | — | — |
| **12b** | 3.87, d (17.5) |  | 3.73, d (17.3) |  | — | — |
| **13** | — | 173.8^b^, C | — | 174.9^b^, C | — | — |

*^a^* Values obtained from Oluwabusola *et al.* (2021)[7] *^b^* Value determined from HMBC spectrum

Supplementary Table S3. ^1^H (600 MHz, CD_3_OD) data of cyclo(Hyp-Leu) (3) and cyclo(Hyp-Phe) (4) in comparison with literature data (*δ* in ppm).

|  | **cyclo(Hyp-Leu) (3)** | **cyclo(*L*-Hyp-*L*-Leu)^a^** | **cyclo(Hyp-Phe) (4)** | **cyclo(*L*-Hyp-*L*-Phe)^a^** |
| --- | --- | --- | --- | --- |
| **Position** | δ_H_ (*J* in Hz) | δ_H_ (*J* in Hz) | δ_H_ (*J* in Hz) | δ_H_ (*J* in Hz) |
| **1** | — | — | — | — |
| **2** | 4.52, ddd (11.1, 6.5, 1.3) | 4.55, dd (6.6, 1.7) | 4.37, ddd (11.7, 5.9, 1.7) | 4.39, dq (6.0, 1.8) |
| **3** | 2.28, ddt (13.2, 6.5, 1.3) 2.09, ddd (13.2, 11.1, 4.3) | 2.30, dd (13.2, 6.5) 2.12, m | 2.07, dd (13.0, 6.0), br 1.37, ddd (12.9, 11.8, 4.6) | 2.09, dd (13.0, 5.9) 1.41, ddd (20.8, 12.0, 0.6) |
| **4** | 4.46, dd (4.3), br | 4.48, t (4.4) | 4.28, dd (4.8), br | 4.29, t (4.8) |
| **5** | 3.66, dd (12.8, 4.5) 3.44, d (13.0), br | 3.68, dd (12.8, 4.4) 3.45, d (12.0) | 3.71, dd (13.0, 5.1) 3.29, m, ov | 3.72, dd (13.0, 5.1) 3.34, m |
| **6** | — | — | — | — |
| **7** | 4.17, m | 4.19, m | 4.49, m | 4.50, dt, (5.2, 1.8) |
| **8** | 1.92, m, ov 1.52, m | 1.92, m 1.55, m | 3.17, dd, br | 3.18, m |
| **9** | 1.89, m, ov | 1.90, m | — | — |
| **10** | 0.97, d (6.4) | 0.97, d (6.4) | 7.28, m | 7.27, m |
| **11** | 0.96, d (6.4) | 0.96, d (6.4) | 7.24, m, ov | 7.30, m |
| **12** | — | — | 7.22-7.25, m, ov | 7.23, m |

^a^ Values obtained from Xiang *et al.*(2020)

**Supplementary Table S4.** Local gene identities between *kin* cluster and related amychelin, cahuitamycin, and gobichelin clusters.

| CDS Name | Identifier  in BGC | BlastP sequence identity to  amychelin (BGC0000300.5) | Identity / Coverage (%) | BlastP sequence identity to cahuitamycin (BGC0001351.) | Identity / Coverage (%) | BlastP sequence identity to  gobichelin BGC | Identity / Coverage (%) |
| --- | --- | --- | --- | --- | --- | --- | --- |
| kinG | ctg2_2469 | - | - | - | - | - | - |
| kinX_2_ | ctg2_2470 | - | - | - | - | - | - |
| kinL | ctg2_2471 | amcL (SSMG_02545)  salicylate_synthase | 49.41 / 91.40 | cahI (AMK48233.1)  salicylate_synthase | 49.55 / 93.12 | - | - |
| kinH | ctg2_2472 | amcH (SSMG_02542)  2,3-dihydroxybenzoate-AMP_ligase | 58.46 /  95.76 | cahJ (AMK48234.1)  salicylate-AMP_ligase | 63.16 / 97.24 | gobK (AGE11892.1)  2,3-dihydroxybenzoate-AMP_ligase | 58.59 /  96.49 |
| kinI_S_ | ctg2_2473 | amcM (SSMG_02534)  iron compound ABC transporter | 27.64 /  75.44 | - | - | gobM (AGE11894.1)  iron_siderophore_uptake_ABC_system | 53.43 /  97.08 |
| kinM | ctg2_2474 | amcA (SSMG_02541)  major facilitator superfamily transporter multidrug resistance protein | 46.60 /  86.99 | cahH (AMK48232.1)  siderophore_export_protein | 46.39 / 94.75 | - | - |
| kinB | ctg2_2475 | amcF (SMG_02536)  NRPS | 44.47 / 71.71 | cahB (AMK48226.1)  NRPS | 44.39 / 71.63 | gobR (AGE11898.1)  NRPS | 46.39 /  70.97 |
| kinC | ctg2_2476 | amcE (SSMG_02537)  NRPS | 44.93 / 57.42 | cahD (AMK48228.1)  NRPS | 45.40 / 40.34 | gobR (AGE11898.1)  NRPS | 38.64 /  58.46 |
| kinD | ctg2_2477 | amcC (SSMG_02539)  mbtH_protein | 65.67 / 97.10 | cahE (AMK48229.1)  MbtH-like_protein | 68.66 / 97.10 | gobL (AGE11893.1)  MbtH | 59.38 /  92.75 |
| kinE | ctg2_2478 | - | - |  |  | - | - |
| kinA | ctg2_2479 | amcG (SSMG_02535)  NRPS | 41.21 / 99.07 | cahA (AMK48225.1)  NRPS | 41.00/ 98.23 | gobJ (AGE11891.1)  NRPS | 41.52 /  99.25 |
| kinT | ctg2_2480 | amcG (SSMG_02535)  NRPS | 39.06 / 19.16 | cahA (AMK48225.1)  NRPS | 43.06 / 21.56 | gobJ (AGE11891.1)  NRPS | 46.56 /  21.86 |
| kinI_Ta_ | ctg2_2481 | amcI (SSMG_02543)  transport system permease protein | 54.55 / 89.80 | cahT2 (AMK48235.1)  iron_ABC_transporter_permease | 49.56 / 98.83 | gobP (AGE11897.1)  iron_siderophore_transporter | 57.79 /  89.80 |
| kinI_Tb_ | ctg2_2482 | amcJ (SSMG_02544)  transport system permease protein | 42.42 / 91.22 | cahT3 (AMK48236.1)  iron_ABC_transporter_permease | 47.18 / 84.99 | gobO (AGE11896.1)  ABC_transporter | 58.91 /  98.02 |
| kinI_N_ | ctg2_2483 | - | - | cahT4 (AMK48243.1)  putative_ABC_transporter_ATPase | 41.11 / 31.90 | gobN (AGE11895.1)  ABC_transporter | 64.73 /  92.47 |
| kinF | ctg2_2484 | - | - | - | - | - | - |
| kinO* | ctg2_1302 | amcP (WP_009078944.1) lysine_N(6)-hydroxylase/L-ornithine_N(5)-oxygenase_family_protein | 67.06 /  94.78 | -^¥^ | -^¥^ | -^¥^ | -^¥^ |

(*) gene located outside of *kin* cluster; (-) no match in cluster identified; (-^¥^) no match outside of BGC identified due to genome unavailability

**Supplementary Table S5.** Closest NCBI Blast and antiSMASH database matches to the core biosynthetic enzymes KinA, KinB, and KinC in the *kin* cluster.

| CDS Name | Identifier  in BGC | Hit Record | Organisms | Amino acid Identity [%] | Database |
| --- | --- | --- | --- | --- | --- |
| kinB | ctg2_2475 | NZ_LT629701 | *Streptomyces atratus* SCSIO_ZH16 | 47.48 | AntiSMASH v4^a^ |
|  |  | NZ_CP027306 | *Alllokutzneria albata* DSM 44149 | 47.18 | AntiSMASH v4 |
|  |  | NZ_WOFH01000003 | *Actinomadura litoris* NEAU-AAG5 | 47.78 | AntiSMASH v4 |
|  |  | WP_091382965.1 | *Actinokineospora alba* | 92.67 | RefSeq non-redundant proteinsb[8] |
|  |  | WP_187220517.1 | *Actinokineospora* *xionganensis* | 92.11 | RefSeq non-redundant proteins |
|  |  | WP_431423574.1 | *Actinokineospora* sp. | 91.51 | RefSeq non-redundant proteins |
| kinC | ctg2_2476 | NZ_JANCLX010000004 | *Streptomyces* *cucumeris* | 40.31 | AntiSMASH v4 |
|  |  | NZ_WPBY01000002 | *Streptomyces* sp. | 39.99 | AntiSMASH v4 |
|  |  | NZ_CP114036 | *Streptomyces* sp. | 39.36 | AntiSMASH v4 |
|  |  | WP_431423573.1 | *Actinokineospora* sp. | 90.73 | RefSeq non-redundant proteins |
|  |  | WP_187220518.1 | *Actinokineospora xionganensis* | 89.56 | RefSeq non-redundant proteins |
|  |  | WP_091382965.1 | *Actinokineospora alba* | 60.80 | RefSeq non-redundant proteins |
| kinA | ctg2_2479 | NZQHCP01000002 | *Actinokineospora spheciospongiae* CECT 8578 | 54.87 | AntiSMASH v4 |
|  |  | NZ_CP09251 | *Streptomyce*s sp. TYQ1024 | 54.07 | AntiSMASH v4 |
|  |  | NZ_JIAI01000001 | *Pseudonocardia acaciae* DSM 45401 | 53.64 | AntiSMASH v4 |
|  |  | WP_166658022.1 | *Actinokineospora* *alba* | 94.04 | RefSeq non-redundant proteins |
|  |  | WP_431423569.1 | *Actinokineospora* sp. | 93.01 | RefSeq non-redundant proteins |
|  |  | WP_187220520.1 | *Actinokineospora* *xionganensis* | 91.61 | RefSeq non-redundant proteins |

^a^ Blin et al., 2024[9], ^b^ Pruitt et al., 2025[8]

**Supplementary Table S6.** Closest homologues of coding sequences (CDSs) within the *kin* biosynthetic gene cluster of *Actinokineospora* sp. UV203.

| CDS Name | Identifier  in BGC | Length  [AA] | Closest homologue | Organism of rigin | Identity [%] | Alignment length [AA] | Homologue accession number |
| --- | --- | --- | --- | --- | --- | --- | --- |
| kinG | ctg2_2469 | 511 | [amidohydrolase](https://blast.ncbi.nlm.nih.gov/Blast.cgi#alnHdr_WP_228770232) | *Actinokineospora* *alba* | 92.76 | 516 | [WP_228770232.1](https://www.ncbi.nlm.nih.gov/protein/WP_228770232.1?report=genbank&log$=prottop&blast_rank=1&RID=848Y7C4S015) |
| kinX_2_ | ctg2_2470 | 111 | hypothetical protein | *Actinokineospora* *xionganensis* | 87.50 | 88 | [WP_187220511.1](https://www.ncbi.nlm.nih.gov/protein/WP_187220511.1?report=genbank&log$=prottop&blast_rank=1&RID=848YATR9014) |
| kinL | ctg2_2471 | 465 | salicylate synthetase | *Actinokineospora alba* | 97.57 | 465 | WP_228770233.1 |
| kinH | ctg2_2472 | 542 | [(2,3-dihydroxybenzoyl)adenylate synthase](https://blast.ncbi.nlm.nih.gov/Blast.cgi#alnHdr_WP_091382958) | *Actinokineospora alba* | 95.94 | 542 | [WP_091382958.1](https://www.ncbi.nlm.nih.gov/protein/WP_091382958.1?report=genbank&log$=prottop&blast_rank=1&RID=848YG6U7015) |
| kinI_S_ | ctg2_2473 | 342 | ABC transporter substrate-binding protein | *Actinokineospora xionganensis* | 94.15 | 342 | [WP_187220515.1](https://www.ncbi.nlm.nih.gov/protein/WP_187220515.1?report=genbank&log$=prottop&blast_rank=1&RID=8496RSVW015) |
| kinM | ctg2_2474 | 438 | enterobactin transporter EntS | *Actinokineospora* sp. | 94.08 | 437 | [WP_431423575.1](https://www.ncbi.nlm.nih.gov/protein/WP_431423575.1?report=genbank&log$=prottop&blast_rank=1&RID=848YNSKA015) |
| kinB | ctg2_2475 | 3627 | [non-ribosomal peptide synthetase](https://blast.ncbi.nlm.nih.gov/Blast.cgi#alnHdr_WP_091382965) | *Actinokineospora* *alba* | 92.67 | 4630 | [WP_091382965.1](https://www.ncbi.nlm.nih.gov/protein/WP_091382965.1?report=genbank&log$=prottop&blast_rank=1&RID=848YTKW7015) |
| kinC | ctg2_2476 | 2506 | amino acid adenylation domain-containing protein | *Actinokineospora* sp. | 90.73 | 2507 | [WP_431423573.1](https://www.ncbi.nlm.nih.gov/protein/WP_431423573.1?report=genbank&log$=prottop&blast_rank=1&RID=8496UVB4014) |
| kinD | ctg2_2477 | 69 | [MbtH family protein](https://blast.ncbi.nlm.nih.gov/Blast.cgi#alnHdr_WP_091382967) | *Actinokineospora* sp. | 98.55 | 69 | [WP_091382967.1](https://www.ncbi.nlm.nih.gov/protein/WP_091382967.1?report=genbank&log$=prottop&blast_rank=1&RID=8496XC16014) |
| kinE | ctg2_2478 | 402 | [SagB family peptide dehydrogenase](https://blast.ncbi.nlm.nih.gov/Blast.cgi#alnHdr_WP_187220519) | *Actinokineospora xionganensis* | 95.52 | 402 | [WP_187220519.1](https://www.ncbi.nlm.nih.gov/protein/WP_187220519.1?report=genbank&log$=prottop&blast_rank=1&RID=84970RH8014) |
| kinA | ctg2_2479 | 1072 | non-ribosomal peptide synthetase | *Actinokineospora alba* | 94.04 | 1073 | [WP_166658022.1](https://www.ncbi.nlm.nih.gov/protein/WP_166658022.1?report=genbank&log$=prottop&blast_rank=1&RID=84973R23014) |
| kinT | ctg2_2480 | 334 | alpha/beta fold hydrolase | *Actinokineospora alba* | 92.22 | 334 | [WP_133794513.1](https://www.ncbi.nlm.nih.gov/protein/WP_133794513.1?report=genbank&log$=prottop&blast_rank=1&RID=84975VBP014) |
| kinI_Ta_ | ctg2_2481 | 343 | FecCD family ABC transporter permease | *Actinokineospora xionganensis* | 95.92 | 343 | WP_312880281.1 |
| kinI_Tb_ | ctg2_2482 | 353 | [FecCD family ABC transporter permease](https://blast.ncbi.nlm.nih.gov/Blast.cgi#alnHdr_WP_091382973) | *Actinokineospora alba* | 97.73 | 353 | [WP_091382973.1](https://www.ncbi.nlm.nih.gov/protein/WP_091382973.1?report=genbank&log$=prottop&blast_rank=1&RID=8497FE4B015) |
| kinI_N_ | ctg2_2483 | 279 | [ABC transporter ATP-binding protein](https://blast.ncbi.nlm.nih.gov/Blast.cgi#alnHdr_WP_431423564) | *Actinokineospora* sp. | 96.77 | 279 | [WP_431423564.1](https://www.ncbi.nlm.nih.gov/protein/WP_431423564.1?report=genbank&log$=prottop&blast_rank=1&RID=8497NZC2015) |
| kinF | ctg2_2484 | 60 | (2Fe-2S)-binding protein | *Actinokineospora* sp. | 91.67 | 60 | [WP_431423563.1](https://www.ncbi.nlm.nih.gov/protein/WP_431423563.1?report=genbank&log$=prottop&blast_rank=1&RID=849J361X014) |
| kinO* | ctg2_1302 | 441 | lysine N(6)-hydroxylase/L-ornithine N(5)-oxygenase family protein | *Actinokineospora alba* | 97.51 | 441 | WP_091382199.1 |

(*) gene located outside of *kin* cluster

**Supplementary Table S7.** Microbial strains used for preliminary antimicrobial susceptibility testing of the kineochelins.

| **Type** | **Classification** | **Taxonomy** | **Strain ID** | **Origin** | **Medium used** | **Inhibition** |
| --- | --- | --- | --- | --- | --- | --- |
| G+ bacteria | Bacillota | *Brevibacillus sp.* | UV260 | environmental, Antarctic | R2A | - |
|  |  | *Bacillus subtilis* | P12204 | environmental, Antarctic | MHA | - |
|  |  | *Paenibacillus* sp. | P12302 | environmental, Antarctic | R2A | yes |
|  |  | *Paenibacillus* sp. | UV231 | environmental, Antarctic | R2A | yes |
|  |  | *Paenisporosarcina* sp. | P12233 | environmental, Antarctic | MHA | - |
|  |  | *Staphylococcus aureus* | 708 | clinical | MHA | - |
|  |  | *Enterococcus faecalis* | 328 | clinical | MHA | - |
|  |  | *Streptococcus pyogenes* | 556 | clinical | MHA | yes |
|  | Actinomycetota | *Nocardioides* sp. | UV208 | environmental, Antarctic | GPHF | - |
|  |  | *Rhodococcus* sp. | UV292 | environmental, Antarctic | MHA | - |
|  |  | *Gordonia sp.* | P12182 | environmental, Antarctic | MHA | yes |
|  |  | *Kytococcus sedentarius* | UV219 | environmental, Antarctic | MHA | yes |
|  |  | *Cryobacterium* sp. | UV168 | environmental, Antarctic | R2A | yes |
|  |  | *Micrococcus luteus* | CCM 169^T^ | environmental | MHA | yes |
|  |  | *Arthrobacter* sp. | P12200 | environmental, Antarctic | MHA | - |
|  |  | *Pseudoarthrobacter* sp. | UV13 | environmental, Antarctic | MHA | yes |
| G- bacteria | Pseudomonadota  (Alphaproteobacteria) | *Sphingomonas* sp. | UVA17_B | environmental, Antarctic | R2A | yes |
|  |  | *Sphingorhabdus* sp. | UVA30_5 | environmental, Antarctic | R2A | yes |
|  |  | *Methylobacterium variabile* | UVA24 | environmental, Antarctic | R2A | yes |
|  |  | *Pararhizobium sp.* | UVA25 | environmental, Antarctic | MHA | yes |
|  | Pseudomonadota  (Betaproteobacteria) | *Masillia sp.* | UV278 | environmental, Antarctic | R2A | yes |
|  |  | *Burkholderia cenocepacia* | H111 | clinical | MHA | yes |
|  | Pseudomonadota  (Gammaproteobacteria) | *Pseudomonas* sp. | UV261 | environmental, Antarctic | MHA | - |
|  |  | *Escherichia coli* | 328 | clinical | MHA | - |
|  |  | *Klebsiella pneumoniae* | 927 | clinical | MHA | - |
|  |  | *Pseudomonas aeruginosa* | 25.211 | clinical | MHA | - |
|  |  | *Acinetobacter baumanii* | JCH 10.26.3 | clinical | MHA | - |
| Yeasts | Ascomycota | *Candida albicans* | ICA1 | clinical | MHA + 2% glucose + MB | yes |
|  |  | *Nakaseomyces glabratus* | 6448 | clinical | MHA + 2% glucose + MB | yes |
|  |  | *Nakaseomyces glabratus* | 8874 | clinical | MHA + 2% glucose + MB | yes |
|  |  | *Saccharomyces cerevisiae* | 5654 | clinical | MHA + 2% glucose + MB | - |
|  |  | *Saprochaete clavata* | 5788 | clinical | MHA + 2% glucose + MB | - |

R2A, Reasoner´s 2 agar; MHA, Mueller-Hinton agar; GPHF, glucose-peptone-yeast-beef medium; MB, methylene blue

**Supplementary Table S8**. Minimum inhibitory concentration (MIC) and Minimum fungicidal concentration (MFC) values recorded for tested yeasts.

| **Strain** | **Disk diffusion assays (mm)** | **MIC**  **(mg/mL)** | **MFC (mg/mL)** |
| --- | --- | --- | --- |
| *Micrococcus luteus* CCM 169^T^ | 40 **^a^** | **-** | **-** |
| *Saccharomyces* *cerevisiae* 5654 | 24 ^b^ | 2.5 | 5.0 |
| *Nakaseomyces glabratus* 6448 | 29 ^b^ | 0.5 | 2.5 |
| *Nakaseomyces* *glabratus* 8874 | 30 ^b^ | 0.5 | 2.5 |

^a^50ul of a 10mg/ml extract; 100ul of a 10mg/ml extract

**Supplementary Table S9**. Results of screening antiproliferative assays with pre-purified kineochelin-enriched fraction.

| **Type of cells** | **Cell line** | **Cancer type** | **Activity (IC_50_) - E1** | **Activity (IC_50_) - E2** |
| --- | --- | --- | --- | --- |
| Cancer cells | A549 | human alveolar adenocarcinoma | 544 ± 31 | 714 ± 15 |
|  | U-87 MG | human glioblastoma | 773 ± 53 | 742 ± 27 |
|  | PaTu 8902 | human pancreatic adenocarcinoma | 783 ± 55 | 1706  ± 96 |
|  | Jurkat | leukaemia | 857 ± 144 | 1051 ± 140 |
|  | HCT116 | human colorectal carcinoma | 899 ± 68 | 494 ± 48 |
|  | A2058 | human metastatic melanoma | 639 ± 32 | 829 ± 57 |
|  | MDA-MB-231 | human mammary gland adenocarcinoma | 1611 ± 150 | 1185 ± 125 |
| Non-cancer cells | CCD-18Co | human colon fibroblasts | 8247 ± 874 | 1881 ± 580 |

**Supplementary Table S10.** Information on genome assembly from *Actinokineospora* sp. UV203

|  | **Genome attribute** | **UV203** |
| --- | --- | --- |
| **Genome characteristics** | Genome size (bp) | 6474610 |
|  | DNA G+C content (%) | 69.4 |
|  | Protein-coding genes | 6,112 |
|  | Pseudo genes | 26 |
|  | rRNA genes (5S, 16S, 23S | 3, 3, 3 |
|  | tRNA genes | 73 |
|  | ncRNA genes | 10 |
|  | CRISPR | 0 |
|  | oriC/oriV | 0 |
|  | oriT | 0 |
| **Genome quality** | Sequencing technology | Oxford Nanopore Technologies |
|  | Number of contigs | 2 |
|  | Largest contig | 6462755 |
|  | Total length | 6474610 |
|  | Completeness | 100 |
|  | Contamination | 1.78 |
|  | Heterogeneity | 0 |
|  | N50 | 6462755 |

**Supplementary Table S11.** Clinical yeast strains used in this study and their antifungal susceptibility determined by minimum inhibitory concentrations according to EUCAST breakpoints.

| **Yeast strain** | **Source of isolation** | **Antifungal susceptibility** | | | | | | | |
| --- | --- | --- | --- | --- | --- | --- | --- | --- | --- |
|  |  | **AMB** | **CAS** | **MIC** | **AND** | **VOR** | **POS** | **FLU** | **ITR** |
| *Saccharomyces* *cerevisiae* 5654 | Invasive infection, blood | S | S | S | S | R | NB | I | I |
| *Saprochaete* *clavata* 5788 | Invasive infection, blood | S | R | R | R | I | I | R | NB |
| *Nakaseomyces glabrata* 6448 | Invasive infection, blood | S | S | S | S | R | R | R | R |
| *Nakaseomyces* *glabrata* 8874 | Invasive infection, blood | S | I | S | S | S | R | I | R |
| *Candida albicans* Ica | urinary tract infection, urine | S | S | S | S | S | S | S | S |

S, sensitive; R, resistant; NB, no breakpoint available; I, intermediate; AMB, amphotericin B; CAS, caspofungin; MIC, micafungin; AND, anidulafungin; VOR, voriconazole; POS, posaconazole; FLU, fluconazole; ITR, itraconazole

**Supplementary Table S12.** List of all primers used in this study.

| ID | Sequence 5´→3´ | Origin |
| --- | --- | --- |
| 1492r | GGTTACCTTGTTACGACTT | Kane et al., 1993[10] |
| 616v | AGAGTTTGATYMTGGCTC | Juretschko et al., 1998[11] |
| aprR_Fwd | GCAACAGTGCCGTTGATCGTGC | This study |
| aprR_Rev | TGCCCCTCCAACGTCATCTCGT | This study |
| LuxR_Fwd | GCAT**GGATCC**GTAGAATCCGGTTCCAGAGGTG* | This study |
| LuxR_Rev | GCAT**GAATTC**ACATCGTGGCGGTGAACGATC | This study |
| Reg2_Fwd | GCAT**GCGGCCGC**GTCCGCCAGAACCCGTTG | This study |
| Reg2_rev | GCAT**GAATTC**TGATGCTGCGCCTGCGCTAC | This study |
| UV203_rrnP_Fw | ACTGTCTA**GAATTC**GGATGTGCGTGTGTTGT | This study |
| UV203_rrnP_Rev1 | GACA**GGATCCC**CAGCGTTCGTCCTGAGC | This study |
| UV203_rrnP_Rev2 | GACA**GCGGCCGC**CCAGCGTTCGTCCTGAGC | This study |
| 2475_F_EcoRI | GACA**GAATTC**CCAGGAACTCCCATTCCAC | This study |
| 2475_R_HindIII | AGTG**AAGCTT**TAGCTCAGGGATTCGCCC | This study |
| 2475_R_HindIII_B | ACTG**AAGCTT**GGCGTCGCCTGCATGAC | This study |
| 2479_F_EcoRI | CTCA**GAATTC**GCGCACCAGCAGGTCA | This study |
| 2479_R_HindIII | AGTC**AAGCTT**CCAGCCCTTGTCCAGCA | This study |
| 2479_R_HindIII_C | ACTG**AAGCTT**GCAGTTGACCATGCCCG | This study |
| QC1_2475_F | CCTGTCCTACGCCGACTACT | This study |
| QC2_2475_R | CGCTGCTGGTGATCAGGTC | This study |
| QC1_2479_F | TGGACTACCTGAGCTGGCAG | This study |
| QC2_2479_R | CCGACCCGATGCCAAATGTATCG | This study |

(*) Restriction sites are highlighted in bold

**Supplementary Table S13.** List of all strains and vectors used in cloning part of this study

|  | ID | Description | Origin |
| --- | --- | --- | --- |
| Bacterial strain | UV203 | *Actinokineospora* sp. UV203, wild-type | This study |
|  | UV203_TC10 | Mutant, harbouring empty vector pSET152 | This study |
|  | UV203_TC3_9 | Mutant, harbouring pSET152-ermE*p + LuxR | This study |
|  | UV203_TC4_2 | Mutant, harbouring pSET152-ermE*p + Reg2 | This study |
|  | UV203_T1A | Mutant, harbouring pSET152- rrn*p + LuxR | This study |
|  | UV203_T2B | Mutant, harbouring pSET152- rrn*p + Reg2 | This study |
|  | *E.coli* DH5α | general cloning host | New England Biolabs |
|  | *E.coli* ET12567 (pUZ8002) | strain for intergenic conjugation; Km^R^, Cm^R^ | Flett et al., 1997[12] |
| Plasmid | pSOK201 | pSG5 minimal replicon, Am^R^, *RP4 oriT*, *ColEI* replication origin | Zotchev et al., 2000[13] |
|  | pSET152 | integrative φC31-based vector, Am^R^ | Bierman et al., 1992[14] |
|  | pSET152 + ermE*p | integrative φC31-based vector, Am^R^, ermE*p | Sioud et al., 2002[15] |
|  | pSET152-ActLuxR | pSET152 + ermE*p and LuxR from UV203 | This study |
|  | pSET152-ActReg2 | pSET152 + ermE*p and LuxR from UV203 | This study |
|  | pSET152-rrnp/LuxR | pSET152 + rrn*p and LuxR from UV203 | This study |
|  | pSET152-rrnp/Reg2 | pSET152 + rrn*p and Reg2 from UV203 | This study |
|  | pSOK201-Δ2475(600) | pSOK201 with 600bp fragment from core gene ctg2_2475 | This study |
|  | pSOK201-Δ2475(1000) | pSOK201 with 1,00bp fragment from core gene ctg2_2475 | This study |
|  | pSOK201-Δ2479(600) | pSOK201 with 600bp fragment from core gene ctg2_2475 | This study |
|  | pSOK201-Δ2479(1000) | pSOK201 with 1,000bp fragment from core gene ctg2_2475 | This study |

Am^R^, apramycine resistance; Km^R^, kanamycin resistance; Cm^R^, chloramphenicol resistance

**References:**

1. Zhao B, Moody SC, Hider RC, Lei L, Kelly SL, Waterman MR, *et al.* Structural Analysis of Cytochrome P450 105N1 Involved in the Biosynthesis of the Zincophore, Coelibactin. *Int. J. Mol. Sci.* 2012;13:8500–13. https://doi.org/10.3390/ijms13078500

2. Bibb MJ, Janssen GR, Ward JM. Cloning and analysis of the promoter region of the erythromycin resistance gene (*ermE*) of *Streptomyces erythraeus*. *Gene*. 1985;38:215–26. https://doi.org/10.1016/0378-1119(85)90220-3

3. Zhao M, Yang Z, Li X, Liu Y, Zhang Y, Zhang M, *et al.* Development of Integrated Vectors with Strong Constitutive Promoters for High-Yield Antibiotic Production in Mangrove-Derived *Streptomyces*. *Mar Drugs*. 2024;22:94. https://doi.org/10.3390/md22020094

4. Zhou X, Wu H, Li Z, Zhou X, Bai L, Deng Z. Over-expression of UDP-glucose pyrophosphorylase increases validamycin A but decreases validoxylamine A production in *Streptomyces hygroscopicus* var. *jinggangensis* 5008. *Metab Eng*. 2011;13:768–76. https://doi.org/10.1016/j.ymben.2011.10.001

5. Klumpp S, Hwa T. Growth-rate-dependent partitioning of RNA polymerases in bacteria. *PNAS.* 2008;105:20245–50. https://doi.org/10.1073/pnas.0804953105

6. Lorenzi J-N, Thibessard A, Lioy VS, Boccard F, Leblond P, Pernodet J-L, *et al.* Ribosomal RNA operons define a central functional compartment in the *Streptomyces* chromosome. *Nucleic Acids Res.* 2022;50:11654–69. https://doi.org/10.1093/nar/gkac1076

7. Oluwabusola ET, Adebisi OO, Reyes F, Acquah KS, Cruz MDL, Mweetwa LL, *et al.* Isolation and characterization of new phenolic siderophores with antimicrobial properties from *Pseudomonas* sp. UIAU-6B. *Beilstein J Org Chem.* 2021;17:2390–8. https://doi.org/10.3762/bjoc.17.156

8. Goldfarb T, Kodali VK, Pujar S, Brover V, Robbertse B, Farrell CM, *et al.* NCBI RefSeq: reference sequence standards through 25 years of curation and annotation. *Nucleic Acids Res.* 2025;53:D243–57. https://doi.org/10.1093/nar/gkae1038

9. Blin K, Shaw S, Medema MH, Weber T. The antiSMASH database version 4: additional genomes and BGCs, new sequence-based searches and more. *Nucleic Acids Res*. 2024;52:D586–9. https://doi.org/10.1093/nar/gkad984

10. Kane MD, Poulsen LK, Stahl DA. Monitoring the enrichment and isolation of sulfate-reducing bacteria by using oligonucleotide hybridization probes designed from environmentally derived 16S rRNA sequences. *Appl Environ Microbiol*. 1993;59:682–6. https://doi.org/10.1128/aem.59.3.682-686.1993

11. Juretschko S, Timmermann G, Schmid M, Schleifer K-H, Pommerening-Röser A, Koops H-P, *et al.* Combined Molecular and Conventional Analyses of Nitrifying Bacterium Diversity in Activated Sludge: Nitrosococcus mobilis and Nitrospira-Like Bacteria as Dominant Populations. A*ppl Environ Microbiol*; 1998;64:3042–51. https://doi.org/10.1128/AEM.64.8.3042-3051.1998

12. Flett F, Mersinias V, Smith CP. High efficiency intergeneric conjugal transfer of plasmid DNA from Escherichia coli to methyl DNA-restricting streptomycetes. *FEMS Microbiol Lett*. 1997;155:223–9. https://doi.org/10.1111/j.1574-6968.1997.tb13882.x

13. Zotchev S, Haugan K, Sekurova O, Sletta H, Ellingsen TE, Valla S. Identification of a gene cluster for antibacterial polyketide-derived antibiotic biosynthesis in the nystatin producer *Streptomyces noursei* ATCC 11455. *Microbiology*. 2000;146 ( Pt 3):611–9. https://doi.org/10.1099/00221287-146-3-611

14. Bierman M, Logan R, O’Brien K, Seno ET, Nagaraja Rao R, Schoner BE. Plasmid cloning vectors for the conjugal transfer of DNA from *Escherichia coli* to *Streptomyces* spp. *Gene.* 1992;116:43–9. https://doi.org/10.1016/0378-1119(92)90627-2

15. Sioud S, Aigle B, Karray-Rebai I, Smaoui S, Bejar S, Mellouli L. Integrative Gene Cloning and Expression System for Streptomyces sp. US 24 and Streptomyces sp. TN 58 Bioactive Molecule Producing Strains. *J Biomed Biotechnol.* 2009;2009:464986. https://doi.org/10.1155/2009/464986
